# Comparative Effectiveness and Safety of Once-Weekly Injectable Semaglutide Versus Dulaglutide in Individuals with Type 2 Diabetes Managed in UK Primary Care: A Population-Based Cohort Study

**DOI:** 10.1016/j.lanepe.2026.101738

**Published:** 2026-06-08

**Authors:** Franziska S. Ulrich, Morten Frost Nielsen, Nicola Napoli, Andrea M. Burden

**Affiliations:** a Institute of Pharmaceutical Sciences, ETH Zurich, Zurich, Switzerland; b Department of Mathematics, ETH Zurich, Zurich, Switzerland; c Department of Endocrinology, Odense University Hospital, Odense, Denmark; d Steno Diabetes Centre Odense, Odense University Hospital, Odense, Denmark; e Unit of Endocrinology and Diabetes, Campus Bio-Medico University of Rome, Rome, Italy; f Unit of Metabolic Bone and Thyroid Disorders, Fondazione Policlinico Universitario Campus Bio-Medico University of Rome, Rome, Italy; g Division of Bone and Mineral Diseases, Washington University, St Louis, MO, USA; h Division of Clinical Immunology and Rheumatology, Heersink School of Medicine, University of Alabama at Birmingham, Birmingham, AL, USA

**Keywords:** Type 2 diabetes, GLP-1 receptor agonist, Semaglutide, Dulaglutide, HbA1c, Bodyweight, Blood pressure, eGFR, Comparative effectiveness and safety, Causal inference, Epidemiology, United Kingdom, Primary care, Electronic medical records, Target trial emulation, Cohort study, Real-world evidence, Cardio-renal-metabolic, Treatment heterogeneity

## Abstract

**Background:**

The SUSTAIN-7 trial demonstrated greater HbA1c and bodyweight reductions with once-weekly semaglutide versus dulaglutide in type 2 diabetes, but strict eligibility criteria limit external validity. We evaluated the comparative real-world effectiveness and safety of these agents in UK primary care.

**Methods:**

Using the IQVIA Medical Research Data (IMRD) incorporating data from THIN, a Cegedim Database, we included adults with type 2 diabetes initiating semaglutide or dulaglutide between 01-Jan-2019 and 01-Dec-2022, followed through 30-Jun-2023. Co-primary outcomes were 1-year changes in HbA1c and bodyweight, estimated using a new-user, active-comparator cohort design with marginal structural models and inverse probability of treatment weighting. Treatment heterogeneity was assessed in the primary per-protocol analysis by SUSTAIN-7 trial-eligibility. Safety events were assessed while-on-treatment.

**Findings:**

Among 6616 new users, 4636 (70.1%) remained on-treatment and 1980 (29.9%) discontinued early. Semaglutide new users (n = 1901) achieved greater 1-year reductions than dulaglutide new users (n = 2735) in HbA1c (estimated treatment difference [ETD] −0.22 percentage points [95% CI −0.30, −0.15]) and bodyweight (ETD −1.92 kg [95% CI −2.91, −0.93]). While semaglutide's benefits were preserved across the 87.7% (n = 4064) not meeting SUSTAIN-7 trial-eligibility (HbA1c: ETD −0.23 percentage points [95% CI −0.31, −0.15]; bodyweight: ETD −2.01 kg [95% CI −3.07, −0.95]), reductions in HbA1c and bodyweight were greater among trial-eligible individuals (12.3%; n = 572) for both agents (trial-eligible [semaglutide/dulaglutide] versus non-eligible [semaglutide/dulaglutide] individuals, HbA1c: −1.10/−1.02 versus −0.83/−0.60 percentage points; bodyweight: −5.72/−4.18 versus −5.50/−3.49 kg). Early discontinuers showed attenuated treatment effects and higher gastrointestinal event rates than those remaining on-treatment (23.5–26.1 versus 10.6–13.8 per 100 person-years).

**Interpretation:**

New users of semaglutide achieved greater reductions in HbA1c and bodyweight than new users of dulaglutide with comparable safety, with benefits preserved across SUSTAIN-7 trial-eligible and non-eligible individuals, supporting preferential use of semaglutide in UK clinical practice.

**Funding:**

10.13039/100000001Swiss National Science Foundation.

**Research in context**

**Evidence before this study**

To identify studies investigating the comparative effectiveness and safety of once-weekly injectable semaglutide and dulaglutide for type 2 diabetes in routine clinical practice, we searched Embase on November 19, 2025, for articles published after January 1, 2018, without language restrictions. Using the following search terms: ‘semaglutide’ AND ‘dulaglutide’ AND (‘t2d’ OR ‘non insulin dependent diabetes mellitus’ OR ‘t2dm’) AND (‘cohort’ OR ‘real world’ OR ‘observational’ OR ‘primary care’ OR ‘general practic∗’ OR ‘clinical practice’ OR ‘effectiveness’ OR ‘efficacy’ OR ‘comparative’), we identified 386 articles.

The SUSTAIN-7 head-to-head trial demonstrated that once-weekly injectable semaglutide is superior to dulaglutide in reducing HbA1c and bodyweight among patients with type 2 diabetes on metformin monotherapy. However, the SUSTAIN-7 trial excluded patients receiving additional glucose-lowering agents (e.g., SGLT-2 inhibitors or insulin) common in routine care and thus, external validity of the findings is limited. Sparse real-world analyses suggest that semaglutide may reduce long-term cardiovascular risk compared with dulaglutide in high-risk cohorts, yet a critical evidence gap remains regarding intermediate cardio-renal-metabolic outcomes such as HbA1c, bodyweight, blood pressure, and eGFR. Bridging this gap is essential, as one-third to one-half of patients discontinue GLP-1 receptor agonist therapy within the first year, making robust evidence on short-to mid-term effectiveness and safety crucial for informed therapeutic decision-making.

**Added value of this study**

Leveraging a UK electronic medical record database, this study provides the first comprehensive real-world, head-to-head comparison of once-weekly injectable semaglutide versus dulaglutide across key cardio-renal-metabolic risk factors in adults with type 2 diabetes. Informed by the SUSTAIN-7 trial protocol and applying an active-comparator, new-user cohort design with marginal structural models and inverse probability weighting, we showed that new users of semaglutide achieved consistently greater reductions in HbA1c, bodyweight, and systolic/diastolic blood pressure than dulaglutide, with similar safety and eGFR profiles.

Treatment effects were directionally consistent across clinical subgroups, including older adults, those with obesity, high baseline HbA1c, or cardiovascular disease. Effects were greater among individuals meeting SUSTAIN-7 trial eligibility criteria. Notably, only 12.3% of real-world initiators would have met the eligibility criteria of the SUSTAIN-7 trial, yet semaglutide's benefits were preserved across the large, heterogeneous population (87.7%) who did not meet eligibility criteria. We additionally provide one of the first real-world assessments of early discontinuation, showing attenuation of treatment benefits among early discontinuers alongside higher gastrointestinal event rates, linking tolerability to treatment discontinuation.

**Implications of all the available evidence**

This study delivers timely evidence confirming that initiation of once-weekly injectable semaglutide offers multi-system benefits sustained across diverse real-world clinical characteristics and populations, including people not represented in clinical trials. The evidence suggests the preferential consideration of semaglutide as a high-efficacy GLP-1 receptor agonist. In a rapidly evolving landscape of expanding GLP-1 receptor agonist indications, such comparative insights are critical for refining treatment algorithms and optimizing care.

## Introduction

Despite effective therapeutic options, many individuals with type 2 diabetes do not meet glycemic targets, increasing the risk for micro- and macrovascular complications, including cardiovascular disease (CVD).^1^ Overweight and obesity, present in more than 85% of individuals with type 2 diabetes, further exacerbate cardio-renal-metabolic risk.^2^^,^^3^ Recognizing this complex interplay, guidelines from the American Diabetes Association (ADA) and the European Association for the Study of Diabetes (EASD) on hyperglycemia management recommend early initiation of a glucagon-like peptide-1 receptor agonist (GLP-1RA) or a sodium-glucose co-transporter-2 inhibitor (SGLT-2i) among individuals with type 2 diabetes at high risk of or with established CVD, heart failure, chronic kidney disease, or obesity.^4^^,^^5^

Within the GLP-1RA class, head-to-head randomized controlled trials (RCTs) are crucial for guiding therapeutic decision-making. The SUSTAIN-7 head-to-head trial demonstrated that once-weekly injectable semaglutide led to greater reductions in HbA1c and bodyweight than dulaglutide at both lower (0.5 mg versus 0.75 mg) and higher (1.0 mg versus 1.5 mg) doses, with comparable safety.^6^ By enrolling only participants treated with metformin monotherapy, the SUSTAIN-7 trial excluded individuals using other glucose-lowering agents (e.g., SGLT-2is, insulin). This limits the generalizability of these findings to routine care, particularly in the UK. Until its update on 18 February 2026, the UK National Institute for Health and Care Excellence (NICE) guidance (NG28) restricted GLP-1RA therapy to individuals for whom triple oral therapy was ineffective or inappropriate and who either had a body mass index (BMI) ≥35 kg/m^2^ or had a BMI <35 kg/m^2^ alongside obesity-related complications or occupational risks associated with insulin use.^7^ Accordingly, real-world evidence from UK primary care showed that GLP-1RA initiation typically occurred at third-line or later stages of therapy, contrasting with contemporary ADA-EASD recommendations that support earlier use.^4^^,^^5^^,^^7^^,^^8^ The updated NG28 guidance moves GLP-1RAs earlier in the treatment pathway, particularly for people with atherosclerotic CVD.^9^ In this evolving policy landscape, comparative real-world evidence between GLP-1RA agents becomes increasingly important to inform individualized therapeutic choice as eligibility expands. This need is further underscored by high real-world discontinuation rates, with approximately one-third to one-half of patients stopping GLP-1RA therapy within one-year, highlighting the relevance of short-to intermediate-term real-world effects.^10^

Against this backdrop, using a comprehensive UK electronic medical record (EMR) database, this study sought to bridge the gap between SUSTAIN-7 trial^6^ efficacy and real-world effectiveness. Preserving core SUSTAIN-7 trial^6^ protocol elements while accounting for the UK clinical and policy setting, our objectives were four-fold: First, to estimate the multi-domain comparative effectiveness (glycemia, weight, blood pressure, estimated glomerular filtration rate [eGFR]) and safety of semaglutide versus dulaglutide in individuals with type 2 diabetes eligible for GLP-1RA therapy under contemporary UK prescribing guidance (NG28, the version in effect before the 18 February 2026 update),^7^ thereby capturing clinical heterogeneity and real-world adherence. Second, to quantify attenuation from RCT efficacy to real-world effectiveness by stratifying analyses according to eligibility for key type 2 diabetes trials: the SUSTAIN-7 trial (adults on metformin monotherapy),^6^ the STEP-2 trial (weight management in adults with overweight or obesity),^11^ and the SUSTAIN-6 trial (CVD outcomes in individuals at high CVD risk).^12^ Third, to assess treatment effect heterogeneity across predefined subgroups (e.g., age, sex assigned at birth, HbA1c, BMI, CVD history).^13^ Finally, to characterize the outcomes and reasons for early discontinuation as direct measure of tolerability and treatment response under UK policy.

## Methods

### Data source

This UK population-based study used the IQVIA Medical Research Data (IMRD) incorporating data from THIN, a Cegedim Database. As of November 2022, the IMRD included UK primary care EMRs encompassing over 25 million patients registered with general practitioner (GP) practices.^14^ The IMRD is comparable to other UK primary care databases (e.g., the Clinical Practice Research Datalink [CPRD] GOLD, which is also based on the Vision clinical system),^15^ and contains longitudinal information on patient demographics, clinical diagnoses and procedures recorded using the Read code classification, laboratory measurements, vital signs, medication prescriptions, and lifestyle factors.^14^

### Study design and eligibility

We designed an active-comparator, new-user cohort study, informed by key components of the SUSTAIN-7 trial protocol (as specified in Supplementary Table S1).^16^^,^^17^ Cohort entry (time-zero) was defined as the date of the first-ever prescription for a GLP-1RA, either semaglutide or dulaglutide, between 01 January 2019 (the year after semaglutide became available)^18^ and 30 June 2023 (Supplementary Table S2 for exposure definitions). Individuals who met any of the following criteria were excluded: age <18-years; <1-year of continuous registration with the GP practice prior to cohort entry; no diagnosis of type 2 diabetes before or at cohort entry; diagnosis of type 1 diabetes before or at cohort entry; prescriptions of more than one GLP-1RA agent at cohort entry; or GLP-1RA initiation after 1 December 2022. Moreover, in line with contemporary NICE guidance (NG28; the version in effect before the 18 February 2026 update), that recommended continuation of GLP-1RA therapy only for patients achieving a dual response at 6-months (HbA1c reduction of at least 1% and bodyweight reduction of at least 3%), we excluded individuals with <210-days of possible follow-up to ensure sufficient time for outcome ascertainment, consistent with the 6-month (180-day) treatment review period plus a 30-day buffer for delayed recording.^7^

All eligible individuals initiating semaglutide or dulaglutide were included in the full cohort (full analysis set). Within the full analysis set, two cohorts were defined: (1) individuals on-treatment for at least 210-days after cohort entry (an observational analog to the SUSTAIN-7 trial per-protocol analysis set)^6^ and (2) individuals with early attrition (i.e., therapy discontinuation within 210-days). The 210-day cut-off was based on the per-protocol definition used in the SUSTAIN-7 trial and aligned with contemporary NICE guidelines (NG28), recommending GLP-1RA therapy continuation beyond 6-months (180-days) conditional on treatment response, with a 30-day buffer for delayed recording.^6^^,^^7^

The per-protocol analysis set (the observational analog to the SUSTAIN-7 trial per-protocol analysis)^7^ was used as the primary analysis cohort to estimate the effects achieved if the medications are taken as intended (i.e., when fully adhering to the treatment; Supplementary Tables S1 and S3).^19^ The full cohort and individuals with early attrition were analyzed as secondary cohorts (Supplementary Tables S1 and S3). Within the full cohort, we assessed the population-level on-treatment effects, while the early-attrition analysis estimated the effects over the first 6-months among individuals who discontinued therapy for various reasons, including the NICE (NG28) 6-month treatment response review,^7^ side effects, or death. As these different analysis sets were designed to provide complementary perspectives on real-world effects, no statistical comparisons between sets were performed.^19^

### Treatment strategies and follow-up

We used an on-treatment approach, with individuals followed while continuously exposed to the treatment strategy assigned at cohort entry. Follow-up began at cohort entry and continued until the earliest occurrence of: (1) the end of coverage of the last prescription for the assigned treatment strategy, if not followed by a subsequent prescription within 90-days (assuming a standard 30-day supply per prescription and a maximum permissible gap of 60-days); (2) initiation of a GLP-1RA agent other than the one assigned at cohort entry; or (3) death, transfer out of the GP practice, end of data availability (30 June 2023), or the end of the predefined study period (510-days after cohort entry). Individuals who switched between GLP-1RA agents did not re-enter the analysis after switching.

### Outcomes

The co-primary outcomes were the absolute change in HbA1c (%), as well as absolute and percentage changes in bodyweight (kg) from baseline to 1-year. Secondary outcomes included absolute changes in BMI (kg/m^2^), systolic/diastolic blood pressure (mmHg), and estimated glomerular filtration rate (eGFR; ml/min/1.73 m^2^). Additional secondary outcomes included the number of individuals achieving an HbA1c reduction of ≥1%, weight loss of ≥3%, ≥5%, and ≥10%, as well as a composite endpoint of absolute HbA1c reduction ≥1% and percentage weight loss ≥3%. In the primary analysis using the per-protocol approach, outcomes were assessed at 1-year to align with trial time frames. In secondary analyses, outcomes were assessed at 6-months to align with the NICE (NG28) treatment response review period.^7^ Consistently, predefined safety events were summarized descriptively (per 100 person-years) among individuals who experienced at least one on-treatment safety event within 6-months. Outcomes were assessed using pre-specified time windows to accommodate irregular timing of real-world clinical assessments. Complete specifications of measurement windows, selection algorithms for individuals with multiple eligible measurements, and handling of missing data are detailed in Supplementary Tables S3 and S8.

### Covariates

We considered a comprehensive set of demographic and clinical characteristics measured at or prior to cohort entry and updated every 30-days throughout follow-up in a time-varying fashion. A complete overview of all covariates, their definitions, timing (time-fixed versus time-varying), and roles in the statistical analysis is provided in Supplementary Table S4. Covariates encompassed demographics (e.g., age, cohort entry year), lifestyle factors (e.g., smoking status), laboratory and vital sign measurements (e.g., eGFR), co-prescribed glucose-lowering therapies (e.g., SGLT-2is), further proxies for type 2 diabetes severity (e.g., type 2 diabetes duration), microvascular complications (e.g., retinopathy), macrovascular complications (e.g., myocardial infarction), other diabetes- and obesity-related comorbidities (e.g., sleep apnea), and concomitant medications (e.g., antihypertensives).

### Statistical analysis

We used marginal structural models (MSMs) with stabilized inverse probability of treatment weights (IPTWs) to estimate the while-on-treatment average treatment effect (ATE), corresponding to the comparative effectiveness of once-weekly injectable semaglutide versus dulaglutide.^20^ Stabilized IPTWs were designed to emulate randomization of treatment strategies at time zero and were derived from propensity scores calculated using multivariable logistic regression to predict the probability of receiving semaglutide versus dulaglutide conditional on covariates measured at cohort entry (Supplementary Table S4). Continuous variables were modeled using cubic splines with five knots (at the 5th, 27.5th, 50th, 72.5th, and 95th centiles). For categorical variables that contained missing data (i.e., smoking status, alcohol use), we included an “unknown” category. We used descriptive statistics to assess covariate balance between cohorts using standardized mean differences (SMDs) before and after weighting, with SMDs <0.10 considered indicative of negligible imbalance.^21^

Missing data in continuous baseline covariates and longitudinal outcomes were addressed using multiple imputation^22^ with chained equations,^23^ assuming a missing-at-random mechanism and performing imputation separately within each treatment arm (Supplementary Tables S5 and S6 for details on imputation and missing data).^24^ Each continuous baseline covariate and longitudinal outcome was imputed using relevant auxiliary information, including repeated measurements of the same outcome at earlier time points.^24^ We generated 100 imputed datasets per treatment arm using Bayesian linear regression, reflecting a balance between computational efficiency and improved inference when increasing numbers of imputations.^23^^,^^25^ Convergence of imputation models was evaluated through visual inspection of trace plots showing means and standard deviations (SDs) of imputed values across iterations. Distributions of observed and imputed values were compared to assess biological plausibility. Treatment effect estimation models were fitted separately within each imputed dataset and results were pooled using Rubin's rules.^22^ The MSMs were fitted using generalized linear models (GLMs) and stabilized IPTWs, with treatment assignment as the exposure (covariates specified in Supplementary Table S4 entered the analysis indirectly through the weights and were not additionally adjusted for in the outcome models). For continuous endpoints (e.g., change in HbA1c), GLMs were used with an identity-link and assumed normally distributed errors. For binary outcomes (e.g., weight loss ≥5%), GLMs were used with a logit-link and binomial error distribution. Robust (Huber-White sandwich) variance estimators were employed to obtain robust standard errors.^26^ Treatment effects were estimated with two-sided 95% confidence intervals (CIs), while p-values were determined according to the null hypothesis of no difference between treatment strategies (α=0.05). Analyses were done with R (version 4.2.2) and SAS (version 9.4; SAS Institute Inc., 2016).

### Subgroup analyses based on the per-protocol set

Co-primary outcomes were assessed separately within subgroups meeting eligibility criteria of the SUSTAIN-7,^6^ STEP-2,^11^ and SUSTAIN-6^12^ trials, as well as their non-eligible counterparts (Supplementary Table S7).

To further assess treatment effect heterogeneity, we evaluated co-primary outcomes separately within predefined subgroups (e.g., age, sex assigned at birth, baseline HbA1c and BMI, history of CVD; Supplementary Table S9).^13^ Moreover, we conducted dose comparisons corresponding to those assessed in the SUSTAIN-7 trial (once-weekly injectable semaglutide versus dulaglutide: low-dose 0.5 mg versus 0.75 mg; high-dose 1.0 mg versus 1.5 mg) and those not formally tested in the SUSTAIN-7 trial (0.5 mg versus 1.5 mg; 1.0 mg versus 0.75 mg; Supplementary Table S9).^6^ For each subgroup and dose-specific analysis, we re-applied the entire analytical framework independently within each stratum: stabilized IPTWs were re-estimated, and covariate balance was re-assessed before fitting the MSMs.

### Exploratory post-hoc subgroup analyses based on the per-protocol set

Post-hoc analyses were conducted within the per-protocol set to evaluate treatment effects according to concomitant use of SGLT-2is and insulin at baseline. Additionally, we compared semaglutide (0.5 mg and 1.0 mg) versus high-dose dulaglutide (3.0 mg and 4.5 mg; Supplementary Table S9). Analytical procedures, including IPTW re-estimation, covariate balance assessment, and MSM fitting were repeated for each post-hoc stratum.

### Sensitivity analyses

Twelve sensitivity analyses (detailed in Supplementary Table S1) were performed on co-primary outcomes to assess the robustness of our findings to: (1) an alternative approach for handling missing data (i.e., complete case analysis); (2) informative censoring (e.g., intention-to-treat analysis or using stabilized time-varying inverse probability of censoring weights [IPCWs; Supplementary Table S4]); (3) different exposure definitions (varying permissible gaps between consecutive prescriptions from 90-days to 60-, 120-, and 180-days); and (4) the breadth of the measurement window for endpoint assessment (utilizing narrower intervals of ±90, ±60, and ±30 days around the 1-year outcome timing).

### Ethics approval

This study was reviewed and approved by the IQVIA Scientific Review Committee (reference 23SRC017; 18 November 2025). Ethical approval for using the IMRD was granted by the NHS Health Research Authority, East Midlands–Derby Research Ethics Committee (REC; reference 23/EM/0151; 10 August 2023).^14^ Informed consent was not required, because the study used anonymized EMRs with no direct patient identifiers and was conducted in accordance with UK regulatory requirements.^14^

### Role of the funding source

The funder of the study had no role in study design, data collection, data analysis, data interpretation, or writing of the report.

## Results

### Study cohort characteristics

**Fig. 1:**
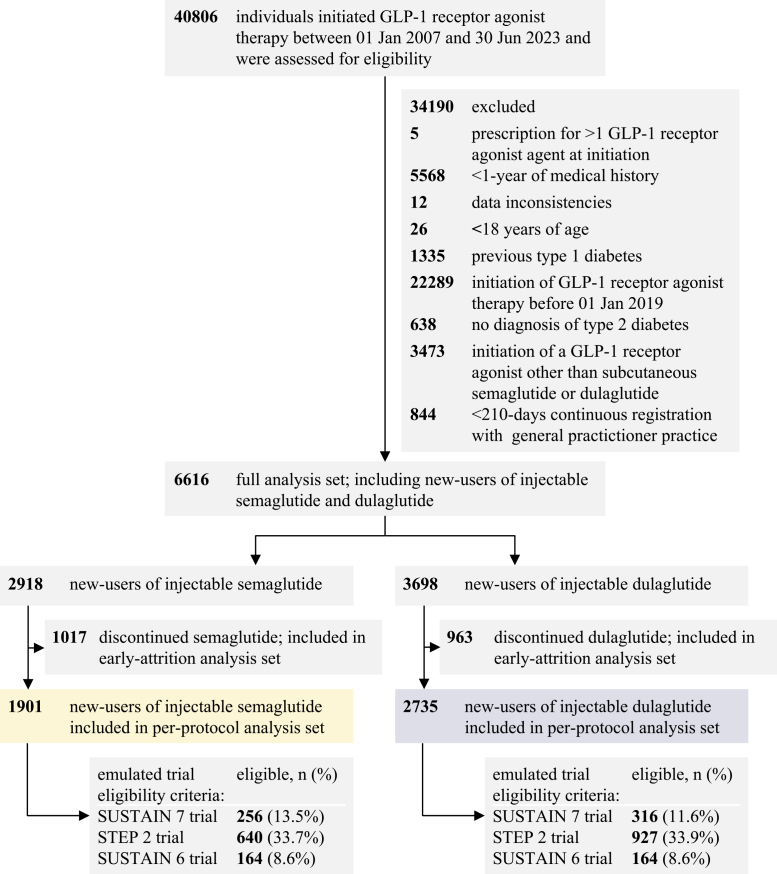
**Study cohort profile of individuals with type 2 diabetes initiating once-weekly injectable semaglutide or dulaglutide in UK primary care between 2019 and 2022.** To provide different perspectives on the treatment effects across real-world adherence trajectories and time frames, we defined several analysis sets: the (1) full analysis set included eligible individuals who received at least one prescription for once-weekly semaglutide or dulaglutide. Within the full analysis set, individuals were divided into: the (2) per-protocol analysis set (an observational analog to the SUSTAIN-7 trial per-protocol analysis set,^6^ including individuals who stayed on their assigned treatment strategy for at least 210-days after cohort entry) and the (3) early-attrition analysis set (including those who discontinued their treatment strategy within 210-days). Among individuals in the per-protocol analysis set, we estimated treatment effects under the assumption that medications were taken as intended. To further assess the efficacy-effectiveness gap, we additionally estimated effects by subgroups who met eligibility criteria of three major trials (SUSTAIN-7,^6^ STEP-2,^11^ and SUSTAIN-6^12^). Abbreviations: GLP-1 glucagon-like peptide-1, SUSTAIN Semaglutide Unabated Sustainability in Treatment of Type 2 Diabetes, STEP Semaglutide Treatment Effect in People With Obesity.

Between 2019 and 2022, 6616 eligible adults with type 2 diabetes initiated once-weekly injectable semaglutide (n = 2918; 44.1%) or dulaglutide (n = 3698; 55.9%) as their first GLP-1RA in UK primary care (Fig. 1). Of these, 4636 (70.1%) adhered to their treatment strategy (per-protocol analysis set) and 1980 (29.9%) discontinued earlier (early-attrition analysis set).

The distributions of baseline demographic and clinical characteristics were similar before and after weighting across all three analysis sets (full cohort, per-protocol, and early-attrition; Supplementary Tables S10–S12). After weighting, covariates were deemed well balanced (SMDs <0.05), and we observed good overlap between the propensity score distributions (Supplementary Figures S1–S3). Table 1 shows selected baseline characteristics of individuals in the full cohort and the per-protocol analysis set after weighting, stratified by treatment strategy.

**Table 1:** Selected baseline demographic and clinical characteristics of once-weekly injectable semaglutide versus dulaglutide initiators after weighting, across the full analysis set and the per-protocol (primary) analysis set.^a^

Characteristics	Full analysis set			Per-protocol analysis set		
	Semaglutide (n = 2918)	Dulaglutide (n = 3698)	SMD	Semaglutide (n = 1901)	Dulaglutide (n = 2735)	SMD
**Demographic characteristics and lifestyle factors**						
Age, years	59.3 (11.7)	59.3 (11.8)	0.001	59.1 (11.5)	59.1 (11.5)	0.001
Sex assigned at birth: Female	1393 (47.8%)	1777 (48.0%)	0.006	896 (47.1%)	1302 (47.6%)	0.011
Type 2 diabetes duration, years	6.4 (6.0)	6.4 (6.0)	0.004	6.3 (5.9)	6.4 (6.0)	0.008
Smoking status: Current	423 (14.5%)	538 (14.6%)	0.002	278 (14.6%)	402 (14.7%)	0.003
Alcohol consumption status: Current	1872 (64.2%)	2369 (64.1%)	0.002	1235 (64.9%)	1767 (64.6%)	0.006
**Co-prescribed glucose-lowering therapies**						
Metformin	2471 (84.7%)	3132 (84.7%)	0.001	1654 (86.9%)	2371 (86.7%)	0.007
Sulfonylureas	1098 (37.6%)	1389 (37.6%)	0.002	726 (38.2%)	1040 (38.0%)	0.003
Sodium-glucose co-transporter-2 inhibitors	1426 (48.9%)	1799 (48.6%)	0.005	957 (50.3%)	1375 (50.3%)	0.001
Dipeptidyl peptidase-4 inhibitors	1254 (43.0%)	1586 (42.9%)	0.002	862 (45.3%)	1233 (45.1%)	0.004
Thiazolidinediones	122 (4.2%)	155 (4.2%)	0.001	77 (4.0%)	112 (4.1%)	0.003
Other glucose-lowering therapies	8 (0.3%)	10 (0.3%)	0.002	<7	<7	0.006
Insulin	441 (15.1%)	561 (15.2%)	0.002	249 (13.1%)	360 (13.2%)	0.003
Number of co-prescribed glucose-lowering therapies ≥3	1323 (45.4%)	1674 (45.3%)	0.002	910 (47.8%)	1301 (47.6%)	0.005
**Laboratory and vital sign measurements**						
Bodyweight, kg	100.8 (19.0)	100.8 (19.1)	0.002	101.4 (18.7)	101.4 (18.8)	0.003
BMI, kg/m^2^	35.1 (5.5)	35.1 (5.6)	0.001	35.2 (5.5)	35.2 (5.5)	0.002
BMI 25–<30 kg/m^2^	480 (16.5%)	608 (16.4%)	0.001	300 (15.8%)	431 (15.8%)	0.002
BMI 30–<35 kg/m^2^	979 (33.6%)	1239 (33.5%)	0.002	642 (33.8%)	923 (33.7%)	0.002
BMI 35–<40 kg/m^2^	823 (28.2%)	1041 (28.1%)	0.001	540 (28.4%)	776 (28.4%)	0.002
BMI ≥40 kg/m^2^	574 (19.7%)	730 (19.7%)	0.002	388 (20.4%)	557 (20.4%)	0.002
HbA1c, %	8.0 (0.7)	8.0 (0.7)	0.001	8.0 (0.7)	8.0 (0.7)	0.002
HbA1c <8%	1242 (42.6%)	1572 (42.5%)	0.001	801 (42.1%)	1149 (42.0%)	0.003
HbA1c ≥ 8%	1676 (57.4%)	2126 (57.5%)	0.001	1101 (57.9%)	1586 (58.0%)	0.003
Systolic blood pressure, mmHg	133.4 (15.0)	133.4 (15.1)	0.002	133.4 (15.1)	133.4 (15.1)	0.004
Diastolic blood pressure, mmHg	78.6 (9.7)	78.6 (9.7)	0.002	78.6 (9.6)	78.7 (9.6)	0.003
eGFR, ml/min/1.73 m^2^	94.2 (22.2)	94.3 (22.2)	0.003	94.4 (22.0)	94.5 (21.9)	0.003
**Type 2 diabetes- and obesity-related comorbidities**						
Cardiovascular disease	555 (19.0%)	702 (19.0%)	0.001	344 (18.1%)	497 (18.2%)	0.003
Myocardial infarction	208 (7.1%)	265 (7.2%)	0.002	128 (6.7%)	184 (6.7%)	0.001
Unstable angina	43 (1.5%)	52 (1.4%)	0.005	24 (1.3%)	30 (1.1%)	0.015
Stroke	175 (6.0%)	224 (6.1%)	0.003	113 (5.9%)	162 (5.9%)	0.002
Stenosis of coronary, carotid, or lower extremity arteries	97 (3.3%)	121 (3.3%)	0.003	63 (3.3%)	88 (3.2%)	0.004
Coronary, carotid, or peripheral arterial revascularization	170 (5.8%)	215 (5.8%)	0.001	102 (5.4%)	147 (5.4%)	0.001
Heart failure	145 (5.0%)	185 (5.0%)	0.002	87 (4.6%)	130 (4.7%)	0.008
Hypertension	1643 (56.3%)	2083 (56.3%)	0.001	1084 (57.0%)	1562 (57.1%)	0.003
Dyslipidemia	566 (19.4%)	717 (19.4%)	0.001	377 (19.8%)	534 (19.5%)	0.008
Non-alcoholic fatty liver disease	265 (9.1%)	337 (9.1%)	0.001	175 (9.2%)	252 (9.2%)	0.001
Chronic kidney disease	461 (15.8%)	587 (15.9%)	0.002	295 (15.5%)	425 (15.6%)	0.001
Retinopathy	552 (18.9%)	703 (19.0%)	0.002	371 (19.5%)	523 (19.1%)	0.009
Neuropathy	418 (14.3%)	531 (14.4%)	0.001	269 (14.1%)	385 (14.1%)	0.002
Cancer	246 (8.4%)	312 (8.4%)	0.001	157 (8.3%)	223 (8.2%)	0.004
Chronic obstructive pulmonary disease	770 (26.4%)	979 (26.5%)	0.001	490 (25.8%)	712 (26%)	0.006
Sleep apnea	136 (4.7%)	173 (4.7%)	0.001	93 (4.9%)	135 (4.9%)	0.002
Osteoarthritis	679 (23.3%)	863 (23.3%)	0.002	436 (22.9%)	631 (23.1%)	0.004
**Comedications**						
Statins	2189 (75.0%)	2767 (74.8%)	0.005	1434 (75.4%)	2053 (75.1%)	0.007
Angiotensin converting enzyme inhibitors	1275 (43.7%)	1614 (43.7%)	0.001	843 (44.3%)	1217 (44.5%)	0.004
Angiotensin II receptor blockers	532 (18.2%)	673 (18.2%)	0.001	358 (18.8%)	505 (18.5%)	0.009
Diuretics	749 (25.7%)	948 (25.6%)	0.001	498 (26.1%)	717 (26.2%)	0.002
Beta blockers	769 (26.3%)	973 (26.3%)	0.001	503 (26.4%)	725 (26.5%)	0.002
Acetylsalicylic acid	535 (18.3%)	684 (18.5%)	0.004	335 (17.6%)	493 (18.0%)	0.011
Selective serotonin reuptake inhibitors	630 (21.6%)	796 (21.5%)	0.002	419 (22.0%)	598 (21.9%)	0.004
Gabapentinoids	323 (11.1%)	413 (11.2%)	0.003	222 (11.7%)	323 (11.8%)	0.004
Opioids	980 (33.6%)	1241 (33.6%)	0.001	646 (34.0%)	934 (34.2%)	0.004
Nonsteroidal anti-inflammatory drugs	536 (18.4%)	677 (18.3%)	0.002	359 (18.9%)	513 (18.8%)	0.003
Proton-pump inhibitors	1334 (45.7%)	1690 (45.7%)	0.001	858 (45.1%)	1233 (45.1%)	0.001
Thyroid hormone replacements	326 (11.2%)	411 (11.1%)	0.002	208 (11%)	301 (11%)	0.002

Data presented in n (%) or mean (SD). Counts <7 are suppressed to prevent person identification. Complete baseline demographic and clinical characteristics before and after weighting are shown in Supplementary Tables S10–S12. Abbreviations: SMD standardized mean difference, BMI body mass index, HbA1c glycated hemoglobin, eGFR estimated glomerular filtration rate.

a Covariate balance was assessed within each imputed dataset using SMDs, which were then averaged across imputations (a pragmatic approach given the lack of consensus on how to evaluate balance after multiple imputation), while descriptive summaries were combined across imputations using Rubin's rules.^22^

Baseline characteristics were highly comparable among semaglutide (n = 1901) and dulaglutide (n = 2735) initiators (SMDs <0.02; Table 1) in the weighted per-protocol analysis set. Specifically, 47.1% versus 47.6% were female, and 47.8% versus 47.6% were co-prescribed ≥3 glucose-lowering therapies (e.g., metformin [86.9% versus 86.7%], SGLT-2is [50.3% in both]). Means for age (59.1 years [SD 11.5]), HbA1c (8.0% [SD 0.7]), and BMI (35.2 kg/m^2^ [SD 5.5]) were identical for both treatment strategies, while type 2 diabetes duration was 6.3 (SD 5.9) versus 6.4 (SD 6.0) years.

### Primary per-protocol analysis

**Fig. 2:**
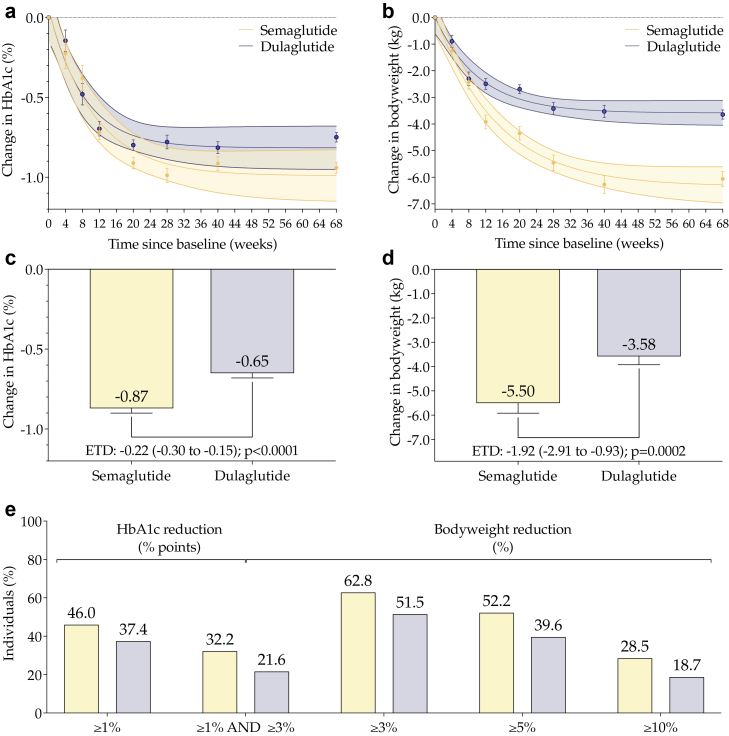
**Glycated hemoglobin (HbA1c) and bodyweight outcomes for once-weekly injectable semaglutide versus dulaglutide measured as change from baseline over 1-year.** Observed changes from baseline in HbA1c (A; percentage points) and bodyweight (B; kg) over time (by week). Changes in HbA1c (C; percentage points) and bodyweight (D; kg) from baseline to 1-year. Observed proportions of individuals achieving an HbA1c reduction of at least 1 percentage point, a composite of HbA1c reduction of at least 1 percentage point and weight loss of at least 3%, as well as bodyweight reductions of at least 3%, 5%, and 10% from baseline to 1-year (E). Values are observed means (SEs; A and B) or estimated means with associated estimated treatment differences (ETDs) and corresponding 95% CIs (C and D) using data from the per-protocol analysis set obtained while on-treatment. Fit (A and B) was estimated using exponential decay (one-phase) with 95% CI. Effectiveness outcomes from baseline over 1-year are further detailed in Table 2. Graphs were created using GraphPad Prism (version 10.2.0 [335]).

Table 2 presents the results of the co-primary and secondary outcome analyses measured from baseline to 1-year in the per-protocol (primary) analysis set, with corresponding changes in mean HbA1c and bodyweight shown in Fig. 2A–E, respectively.

**Table 2:** Effectiveness outcomes measured as change from baseline over 1-year.

Endpoints	Semaglutide (n = 1901)	Dulaglutide (n = 2735)	Treatment comparison (95% CI)	p value
**Primary endpoints measured as absolute and percentage changes from baseline to 1-year**				
Change from baseline HbA1c, percentage points	−0.87 (0.03)	−0.65 (0.03)	ETD −0.22 (−0.30, −0.15)	<0.0001
Change from baseline bodyweight, kg	−5.50 (0.42)	−3.58 (0.34)	ETD −1.92 (−2.91, −0.93)	0.0002
Percentage change from baseline bodyweight, %	−4.76 (0.45)	−2.92 (0.35)	ETD −1.85 (−2.90, −0.79)	0.0006
**Secondary endpoints measured as absolute changesfrom baseline to 1-year**				
BMI, kg/m^2^	−1.97 (0.12)	−1.27 (0.09)	ETD −0.70 (−0.98, −0.42)	<0.0001
Systolic blood pressure, mmHg	−4.25 (0.48)	−2.22 (0.40)	ETD −2.03 (−3.23, −0.82)	0.001
Diastolic blood pressure, mmHg	−1.43 (0.30)	−0.58 (0.25)	ETD −0.85 (−1.61, −0.09)	0.028
eGFR, ml/min/1.73 m^2^	−0.49 (0.27)	−0.64 (0.20)	ETD 0.15 (−0.50, 0.79)	0.66
**Secondary endpoints measured as proportionsof individuals achieving glycemic and weight management targets at 1-year**				
HbA1c reduction ≥1%	874 (46.0%, SE = 1.5)	1023 (37.4%, SE = 1.2)	OR 1.43 (1.22, 1.66)	<0.0001
Percentage bodyweight reduction ≥3%	1194 (62.8%, SE = 1.6)	1409 (51.5%, SE = 1.3)	OR 1.59 (1.34, 1.87)	<0.0001
Percentage bodyweight reduction ≥5%	992 (52.2%, SE = 1.6)	1083 (39.6%, SE = 1.3)	OR 1.66 (1.42, 1.96)	<0.0001
Percentage bodyweight reduction ≥10%	542 (28.5%, SE = 1.4)	511 (18.7%, SE = 1.0)	OR 1.73 (1.43, 2.08)	<0.0001
Composite of HbA1c reduction ≥1% and percentage bodyweight reduction ≥3%	612 (32.2%, SE = 1.5)	591 (21.6%, SE = 1.0)	OR 1.73 (1.44, 2.08)	<0.0001

Data are presented as estimated mean (SE) change from baseline with mean estimated treatment difference (ETD) and corresponding 95% CI, or as n (% and corresponding SE in %) with estimated odds ratio (OR) and corresponding 95% CI; using data from individuals in the per-protocol analysis set (i.e., those who remained on their assigned treatment strategy for more than 210-days after cohort entry), while on-treatment. Abbreviations: HbA1c glycated hemoglobin, BMI body mass index, eGFR estimated glomerular filtration rate.

Semaglutide was associated with greater reductions than dulaglutide from baseline to 1-year in both HbA1c (estimated treatment difference [ETD] −0.22 percentage points [95% CI −0.30, −0.15]; p < 0.0001) and bodyweight (ETD −1.92 kg [95% CI −2.91, −0.93]; p < 0.001). At 1-year, absolute change in mean HbA1c was −0.87 percentage points (SE 0.03) with semaglutide versus −0.65 percentage points (SE 0.03) with dulaglutide, while mean bodyweight was decreased by −5.50 kg (SE 0.42) with semaglutide versus −3.58 kg (SE 0.34) with dulaglutide. Consistently, semaglutide was associated with greater percentage reductions than dulaglutide in bodyweight (ETD −1.85% [95% CI −2.9, −0.79]; p < 0.001), with percentage changes in mean bodyweight of −4.76% (SE 0.45) and −2.92% (SE 0.35), respectively.

At 1-year, greater proportions of patients receiving semaglutide than dulaglutide achieved weight loss of ≥5% (52.2% versus 39.6%) and ≥10% (28.5% versus 18.7%), as well as the composite endpoint of HbA1c reduction ≥1% and weight reduction ≥3% (32.2% versus 21.6%) (all p < 0.0001). Similarly, mean reductions from baseline in BMI were greater with semaglutide than with dulaglutide (ETD −0.70 kg/m^2^ [95% CI −0.98, −0.42]; p < 0.0001).

For systolic blood pressure, the absolute change was −4.25 mmHg (SE 0.48) with semaglutide and −2.22 mmHg (SE 0.40) with dulaglutide (ETD −2.03 mmHg [95% CI −3.23, −0.82]; p = 0.001). For diastolic blood pressure, the corresponding changes were −1.43 mmHg (SE 0.30) and −0.58 mmHg (SE 0.25), respectively (ETD −0.85 mmHg [95% CI −1.61, −0.09]; p = 0.028). For eGFR, the changes were −0.49 ml/min/1.73 m^2^ (SE 0.27) and −0.64 ml/min/1.73 m^2^ (SE 0.20), respectively (ETD 0.15 ml/min/1.73 m^2^ [95% CI −0.50, 0.79]; p = 0.66).

Diagnostics of our multiple imputation approach demonstrated convergence across iterations and alignment between observed and imputed value distributions for both treatment strategies (Supplementary Figures S4–S7). All prespecified sensitivity analyses, including the complete-case analysis as an alternative approach for handling missing data, were consistent with the findings of the primary analysis (Supplementary Table S13).

### SUSTAIN-7,^6^ STEP-2,^11^ and SUSTAIN-6^12^ trial eligibility

Among individuals in the per-protocol analysis set (n = 4636; semaglutide n = 1901, dulaglutide n = 2735), 13.5% receiving semaglutide and 11.6% receiving dulaglutide met eligibility criteria for the SUSTAIN-7 trial,^6^ 33.7% and 33.9% for the STEP-2 trial,^11^ and 8.8% and 8.6% for the SUSTAIN-6 trial,^12^ respectively (Fig. 1 and Supplementary Table S14). Supplementary Table S15 shows the primary outcome analyses stratified by trial eligibility. Semaglutide was associated with greater reductions in HbA1c and bodyweight compared to dulaglutide, while ETDs were largely similar across strata. Reductions in HbA1c and bodyweight were greater for SUSTAIN-7^6^ and STEP-2^11^ trial-eligible individuals for both semaglutide and dulaglutide compared with their non-eligible counterparts but were similar across SUSTAIN-6^12^ trial-eligibility strata.

### Clinical subgroup analyses

Supplementary Table S9 presents the primary analyses stratified by predefined clinical subgroups, with treatment effects of semaglutide versus dulaglutide being directionally consistent across all subgroups. Greater HbA1c reductions were observed among those with baseline HbA1c ≥ 8% and greater weight loss among those with BMI ≥35 kg/m^2^ or those aged ≥65-years across both treatment strategies. We did not observe differences in treatment effects across CVD history strata. Across dose comparisons (Supplementary Table S9), semaglutide was associated with greater reductions in HbA1c and bodyweight than dulaglutide, even when comparing semaglutide 0.5 mg to dulaglutide 1.5 mg. Exploratory post-hoc analyses remained directionally consistent with the primary findings (Supplementary Table S9).

### Secondary analyses in expanded populations

Supplementary Tables S16–S18 present the primary and secondary outcomes for the full cohort at the 1-year and 6-month assessments, and for the per-protocol and early-attrition sets at the 6-month assessment, respectively. Results across all analysis sets were directionally consistent with the primary 1-year per-protocol analysis (Table 2), although treatment effects were slightly attenuated at the 6-month assessment and among individuals with early-attrition.

### Safety analysis

The results of the safety analysis are presented in Supplementary Table S8. Overall, fewer than seven deaths resulted in treatment discontinuation, while proportions and rates (per 100-person-years) of safety events were similar across treatment strategies. Gastrointestinal disorders were common, with event rates being higher in the early-attrition (23.5/100 person-years with semaglutide and 26.1/100 person-years with dulaglutide) compared with the per-protocol analysis set (13.8/100 person-years with semaglutide and 10.6/100 person-years with dulaglutide). Event rates ranged from 1.3 to 2.6 events per 100-person-years for cardiovascular events and from 0.2 to 0.8 events per 100-person-years for hypoglycemia across strata.

## Discussion

Building on the SUSTAIN-7 trial,^6^ we compared once-weekly semaglutide versus dulaglutide to assess the extent to which clinical trial-based estimates translate into real-world comparative effectiveness and safety in routine UK primary care. Semaglutide was associated with greater reductions than dulaglutide across HbA1c, bodyweight, and systolic/diastolic blood pressure, with comparable safety and eGFR effects observed for both agents. Semaglutide's benefits were consistent across complementary analysis sets (per-protocol, early-attrition, full cohort), strata based on SUSTAIN-7,^6^ STEP-2,^11^ and SUSTAIN-6^12^ trial-eligibility, and predefined clinical subgroups (e.g., age, CVD history). While the magnitude of effect was attenuated among non-trial-eligible individuals, the greater effect seen upon restricting the analysis to trial-eligible individuals confirms the utility of benchmarking real-world effectiveness against trial-eligible subpopulations in bridging the efficacy-effectiveness gap. This gap is further highlighted by the low proportions of semaglutide or dulaglutide initiators meeting eligibility criteria for SUSTAIN-7 (12.3%),^6^ STEP-2 (33.8%),^11^ and SUSTAIN-6^12^ (9.4%) trials, reflecting the substantial clinical heterogeneity characteristic of routine practice relative to RCTs.

Collectively, these findings validate and extend the greater efficacy of semaglutide observed in the SUSTAIN-7 trial^6^ to UK clinical practice, demonstrating that the treatment effects are sustained across both trial-eligible and broader, clinically heterogeneous (non-trial-eligible) populations, with particular relevance for high-risk patient groups underrepresented in RCTs.

To facilitate interpretation of our results compared to RCTs, relevant estimates and baseline characteristics are summarized in Supplementary Tables S19 and S20. Our findings were consistent with the SUSTAIN-7 trial, where semaglutide led to greater reductions in HbA1c and bodyweight compared to dulaglutide at both lower (0.5/0.75 mg) and higher (1.0/1.5 mg) doses.^6^ Notably, semaglutide was even associated with greater reductions in HbA1c and bodyweight when comparing its lowest maintenance dose (0.5 mg) to dulaglutide 1.5 mg, consistent with a post-hoc SUSTAIN-7 trial analysis.^27^ Compared to the SUSTAIN-7 trial, HbA1c reductions were modestly attenuated for both agents, while semaglutide's weight loss (−5.50 kg) fell between the trial's lower- (−4.6 kg) and higher-dose (−6.5 kg) arms, and dulaglutide's weight loss (−3.58 kg) exceeded the trial's higher-dose arm (−3.0 kg).^6^

When restricting to individuals meeting SUSTAIN-7 trial eligibility criteria, HbA1c (−1.10 versus −1.02 percentage points) and bodyweight (−5.72 versus −4.18 kg) reductions increased and aligned more closely with trial estimates,^6^ with similar patterns observed among individuals meeting STEP-2 trial eligibility (i.e., those with overweight or obesity).^11^ Restricting to individuals meeting SUSTAIN-6^12^ trial eligibility criteria (i.e., those with high risk for, or established, CVD) did not alter treatment effects, with HbA1c and bodyweight changes consistent with findings from the SUSTAIN-6 (semaglutide) and REWIND (dulaglutide) cardiovascular outcomes trials.^12^^,^^13^

By stratifying analyses by trial-eligibility, we revealed that few individuals with type 2 diabetes initiating semaglutide or dulaglutide in UK primary care would have met eligibility criteria for the SUSTAIN-7 (12.8%),^6^ STEP-2 (33.8%),^11^ and SUSTAIN-6 (9.4%) trials,^12^ primarily due to concomitant glucose-lowering therapy use. Treatment effects observed among trial-eligible individuals more closely approximated RCT estimates, particularly for glycemic control, whereas bodyweight reductions were already comparable prior to stratification, suggesting a narrower efficacy-effectiveness gap for bodyweight than for glycemic endpoints. One potential explanation might be that weight loss is directly observable to patients and clinicians, which may enhance perceived benefits and promote adherence, even in the presence of side effects. Moreover, while comparative effects of semaglutide versus dulaglutide were not influenced largely by predefined clinical subgroups, consistent with prior evidence, we observed greater HbA1c reductions at higher baseline HbA1c levels and more pronounced weight loss at higher baseline BMI for both semaglutide and dulaglutide.^28^, ^29^, ^30^ Greater weight loss was also observed among individuals aged ≥65-years across both treatment strategies, which is concerning given the potential to accelerate sarcopenia and physical frailty in older adults.^31^^,^^32^ These findings suggest that baseline clinical characteristics (e.g., age) may meaningfully modify treatment response, reinforcing the need for further investigation to guide individualized, safe, and effective prescribing strategies.

Consistent with RCTs, gastrointestinal disorders were the most common adverse events and occurred at similar rates across treatment strategies, while higher event rates among individuals with early-attrition suggest gastrointestinal tolerability as a main reason for treatment cessation in routine care.^6^^,^^11^, ^12^, ^13^ Nonetheless, gastrointestinal event frequencies observed in our study were lower (5.5–7.6%) than in the SUSTAIN-7 trial (43–48%), likely reflecting under-reporting of mild events in routine care.^6^ Indeed, in the SUSTAIN-7 trial, 28–42% of participants reported mild gastrointestinal events, whereas the frequencies of moderate (7–16%) and severe (1–3%) events more closely align with those observed in UK primary care.^6^

This UK population-based study demonstrates that rigorously designed real-world evidence studies can both align with trial estimates in RCT-eligible individuals and extend findings to the broader non-trial-eligible population, bridging the efficacy-effectiveness gap. This was achieved by employing robust analytic methods, including an active-comparator, new-user design with MSMs weighted by IPTWs and IPCWs.^16^^,^^17^^,^^19^^,^^20^ Our findings are further supported by several methodological strengths. Leveraging the extensive information captured in the IMRD enabled assessment of cardio-renal-metabolic risk factors typically evaluated in RCTs but often unavailable in administrative databases, while adjusting for relevant confounders (e.g., smoking). Moreover, by aligning follow-up with both clinical trial (1-year) and UK policy (NG28)-relevant (6-month) time frames,^6^^,^^7^ and classifying individuals into complementary analysis sets (per-protocol, early-attrition, full cohort), we captured both expected effects under intended use and pragmatic effects across heterogeneous real-world treatment trajectories. We further assessed treatment effect heterogeneity across eligibility criteria for key trials, clinically relevant subgroups, and dose strata, quantifying the extent of attenuation from trial efficacy to real-world effectiveness and identifying subpopulations in whom treatment responses may be most pronounced. Given the rapid evolution of type 2 diabetes therapeutics, real-world evidence is vital for generating comparative insights that complement and extend RCT findings to diverse populations, supporting timely clinical, regulatory, and policy decisions in the UK and beyond.

This study has several limitations. First, although we used IPTW to emulate randomization, residual confounding cannot be excluded, particularly due to unmeasured information, including ethnicity, race, as well as patient and physician preferences (e.g., preferred agent or injection device). Second, the IMRD captures prescriptions issued by GPs, but not whether medications were taken, and it does not include prescriptions issued in secondary care, which both may have led to exposure misclassifications; however, results of the sensitivity analyses using alternative exposure definitions supported our findings. Third, baseline continuous covariates and longitudinal outcome data were missing for a proportion of individuals. Consistent with prior analyses of UK primary care databases (e.g., Bidulka et al., 2024), we addressed missing data using treatment-arm-specific multiple imputation under a missing-at-random assumption, incorporating an extensive set of auxiliary variables (Supplementary Table S5), including prior measurements of the same outcome, to mitigate the risk of informative missingness (missing-not-at-random) after conditioning on observed data.^24^ While missing-not-at-random mechanisms cannot be entirely ruled out, findings were consistent across complementary analysis sets (including individuals who discontinued early, e.g., due to tolerability or 6-month GLP-1RA response criteria under contemporary NICE guidance^7^), predefined subgroups, and extensive sensitivity analyses. This consistency suggests that bias due to missing data is unlikely to materially affect our conclusions, although residual informative missingness remains an inherent limitation of real-world data. Fourth, using a 1-year follow-up period consistent with RCT protocols,^6^^,^^11^ this study was not designed to detect differences in (longer-term) disease modification or clinical outcomes (e.g., major adverse cardiovascular events, chronic kidney disease [CKD]). Nevertheless, changes in HbA1c, bodyweight, blood pressure, and eGFR over this period remain clinically meaningful for characterizing the early cardio-renal-metabolic response to GLP-1RA initiation. Fifth, limited sample sizes in some subgroups, particularly trial-eligible strata, led to wide confidence intervals, and these estimates should be interpreted as exploratory. Finally, as with all UK primary care EMR databases, the IMRD is subject to variability in data completeness, accuracy, coding practices, as well as heterogeneity in the timing and frequency of clinically recorded measurements collected for routine care rather than research, which may introduce measurement error. During our study period, the IMRD captured data from practices using the Vision clinical system, which has declined in England and has largely been replaced by EMIS.^14^^,^^15^^,^^33^ As recently documented in the CPRD GOLD Data Resource Profile Update (Sanchez-Santos et al., 2025), Vision held only 0–5% market share in England in 2022, compared with 50–60% in Wales, 40–50% in Scotland, and 30–40% in Northern Ireland.^15^ However, prescribing patterns of glucose-lowering therapies in UK primary care are predominantly determined by contemporary NICE guidance (NG28; pre-February 2026) and the structure of the UK healthcare system; consequently, clinical system representativeness across the UK is more relevant for health services research than for within-class comparative effectiveness and safety analyses.^7^, ^8^, ^9^, ^10^^,^^14^^,^^15^^,^^33^

Given the clinical heterogeneity of type 2 diabetes and the imperative to individualize treatment, semaglutide initiators achieved greater reductions in HbA1c, bodyweight, as well as systolic and diastolic blood pressure compared to dulaglutide in UK primary care. Safety profiles (e.g., cardiovascular or gastrointestinal events) were similar between treatment strategies, and no differences were observed in eGFR changes. These findings were directionally consistent across clinical subgroups (age, sex assigned at birth, type 2 diabetes duration, CVD history) and dose comparisons. Greater treatment effects were observed among individuals meeting RCT eligibility criteria, whereas effects were attenuated among those who discontinued early and experienced higher rates of gastrointestinal events. Beyond gastrointestinal tolerability, suboptimal adherence and early discontinuation of GLP-1RA therapy may reflect contemporary NICE guidance (NG28; pre-February 2026), recommending treatment continuation beyond 6-months only if both glycemic and weight loss targets are met.^7^^,^^10^ However, our findings suggest that this dual-target rule excludes individuals deriving clinically meaningful benefit in a single domain or through additional cardio-renal-metabolic effects (e.g., blood pressure reduction). The updated NICE guidance (NG28 published on 18 February 2026) addresses this in part by supporting earlier access to GLP-1RAs for individuals with atherosclerotic CVD and placing greater emphasis on cardio-renal risk modification alongside glycemic control.^9^ As GLP-1RA therapy moves earlier in the treatment pathway, agent choice, rather than class choice alone, becomes progressively more important.^9^ In this setting, our head-to-head comparative estimates across HbA1c, bodyweight, blood pressure, and eGFR provide actionable evidence to inform selection between once-weekly injectable semaglutide and dulaglutide. Emerging precision medicine approaches, including machine learning-derived treatment response phenotypes, may further refine the identification of individuals most likely to benefit from a specific therapy.

Collectively, these findings support a flexible, patient-centered approach to GLP-1RA initiation and continuation that incorporates multidimensional benefit assessment and tolerability. Structured adherence support and proactive communication regarding the benefit–risk profile, particularly in managing early gastrointestinal events, may further improve real-world effectiveness. While this study provides robust real-world evidence extending prior RCT findings to a broader, more heterogeneous population, future research, encompassing newer agents and longer follow-up durations will be important for clarifying the clinical relevance of between-agent differences for cardio-renal-metabolic risk reduction.

## Contributors

AMB and FSU conceived and designed the study. FSU conducted the statistical analyses. AMB, FSU, MFN, and NN contributed to data interpretation. AMB and FSU drafted the manuscript. AMB, FSU, MFN, and NN critically revised the manuscript and approved the final version. AMB and FSU accessed and verified the underlying data and take responsibility for the accuracy of the analysis. AMB, FSU, MFN, and NN were responsible for the decision to submit the manuscript. AMB and FSU are the guarantors of the study.

## Data sharing statement

Access to the database used in this study (IQVIA Medical Research Data [IMRD] incorporating data from THIN, a Cegedim Database) was obtained under license from IQVIA. These data are not publicly available and cannot be shared. Access may be granted to eligible researchers by IQVIA, subject to review and approval.

## Use of artificial intelligence

During the course of preparing this work, the author(s) used OpenAI GPT-5 and GPT-4o for the purpose of improving readability and language of the manuscript, as well as supporting statistical coding. Following the use of this tool/service, the author(s) formally reviewed the content for its accuracy and edited it as necessary. The author(s) take full responsibility for all the content of this publication.

## Declaration of interests

All authors acknowledge support from the Swiss National Science Foundation for the submitted work. AMB and FSU do not declare any conflict of interest. MFN reports consultancy work for Novo Nordisk A/S; employment at Novo Nordisk A/S from June 2024 to February 2025; receipt of drugs for an investigator-initiated trial from Novo Nordisk A/S; and holds stock in Novo Nordisk A/S. NN reports research funding to their institution (UCBM) from Novo Nordisk A/S; honoraria for lectures, presentations, or educational activities from Novo Nordisk A/S and Eli Lilly and Company; and holds stock in Novo Nordisk A/S.
