# Supplementary material for: Comparative Effectiveness and Safety of Once-Weekly Injectable Semaglutide Versus Dulaglutide in Individuals with Type 2 Diabetes Managed in UK Primary Care: A Population-Based Cohort Study
Source: Lancet Reg Health Eur. 2026 Jun 8;67:101738. doi: 10.1016/j.lanepe.2026.101738 (PMC13262276; doi:10.1016/j.lanepe.2026.101738)
Supplement: Supplementary Material [file mmc1.docx]

Online-Only Supplementary Material

Table of Contents

[SUPPLEMENTARY TABLES 3](#_Toc231288478)

[Table S1: Protocol components of the SUSTAIN 7 trial^1^ versus the active-comparator, new-user cohort study. 3](#_Toc231288479)

[Table S2: ATC codes and generic names to define usage and dosage of once-weekly injectable semaglutide and dulaglutide. 9](#_Toc231288480)

[Table S3: Outcome definitions and timing. 10](#_Toc231288481)

[Table S4: Covariate definitions and assessment periods. 12](#_Toc231288482)

[Table S5: Imputed variables and covariates included in imputation models. 15](#_Toc231288483)

[Table S6: Descriptive summary of missing data in continuous baseline covariates and longitudinal outcomes, by analysis set and treatment strategy. 16](#_Toc231288484)

[Table S7: Application of SUSTAIN 7,^1^ STEP 2,^2^ and SUSTAIN 6^3^ trial eligibility criteria to UK primary care data. 17](#_Toc231288485)

[Table S8: On-treatment safety events between treatment initiation and 210-days of follow-up, stratified by analysis set. 28](#_Toc231288486)

[Table S9: Subgroup analyses of 1-year primary endpoints in the per-protocol analysis set, based on REWIND trial subgroup definitions^4^ and SUSTAIN 7 trial dose comparisons.^1^ 29](#_Toc231288487)

[Table S10: Baseline demographic and clinical characteristics of semaglutide versus dulaglutide initiators in the full analysis set, before and after weighting.* 36](#_Toc231288488)

[Table S11: Baseline demographic and clinical characteristics of semaglutide versus dulaglutide initiators in the per-protocol analysis set, before and after weighting.* 39](#_Toc231288489)

[Table S12: Baseline demographic and clinical characteristics of semaglutide versus dulaglutide initiators in the early-attrition analysis set, before and after weighting.* 42](#_Toc231288490)

[Table S13: Sensitivity analyses on primary outcomes (changes in glycated hemoglobin [HbA1c] and bodyweight from baseline to 1-year) conducted among individuals in the per-protocol analysis set. 45](#_Toc231288491)

[Table S14: Individuals considered eligible for the SUSTAIN 7,^1^ STEP 2,^2^ and SUSTAIN 6^3^ trials based on eligibility criteria specified in Supplementary Table S7, stratified by analysis set and treatment strategy. 47](#_Toc231288492)

[Table S15: Subgroup analyses based on eligibility for the SUSTAIN 7,^1^ STEP 2,^2^ and SUSTAIN 6^3^ trials in the per-protocol analysis set for primary endpoints. 51](#_Toc231288493)

[Table S16: Effectiveness outcomes measured over 6-months and 1-year in the full analysis set. 53](#_Toc231288494)

[Table S17: Effectiveness outcomes measured over 6-months in the per-protocol analysis set. 55](#_Toc231288495)

[Table S18: Effectiveness outcomes measured over 6-months in the early-attrition analysis set. 56](#_Toc231288496)

[Table S19: Summary of endpoint estimates from the SUSTAIN 7,^1^ STEP 2,^2^ and SUSTAIN 6^3^ trials. 57](#_Toc231288497)

[Table S20: Selected baseline characteristics of participants (randomized to once-weekly subcutaneous semaglutide 1.0 mg) in the SUSTAIN 7,^1^ STEP 2,^2^ and SUSTAIN 6^3^ trials, along with those of individuals (assigned to once-weekly subcutaneous semaglutide at cohort entry) in the per-protocol analysis set who were considered eligible for the corresponding trials (Supplementary Table S14). 59](#_Toc231288498)

[SUPPLEMENTARY FIGURES 60](#_Toc231288499)

[Figure S1: Propensity score distributions of semaglutide versus dulaglutide initiators in the full analysis set, before and after weighting. 60](#_Toc231288500)

[Figure S2: Propensity score distributions of semaglutide versus dulaglutide initiators in the per-protocol analysis set, before and after weighting. 60](#_Toc231288501)

[Figure S3: Propensity score distributions of semaglutide versus dulaglutide initiators in the early-attrition analysis set, before and after weighting. 61](#_Toc231288502)

[Figure S4: Convergence diagnostics for multiple imputation by chained equations among once-weekly injectable semaglutide initiators. 62](#_Toc231288503)

[Figure S5: Convergence diagnostics for multiple imputation by chained equations among once-weekly injectable dulaglutide initiators. 63](#_Toc231288504)

[Figure S6: Distribution comparison of observed versus imputed outcome values among once-weekly injectable semaglutide initiators. 64](#_Toc231288505)

[Figure S7: Distribution comparison of observed versus imputed outcome values among once-weekly injectable dulaglutide initiators. 65](#_Toc231288506)

[SUPPLEMENTARY REFERENCES 66](#_Toc231288507)

SUPPLEMENTARY TABLES

| **Table S1: Protocol components of the SUSTAIN 7 trial**^1^ **versus the active-comparator, new-user cohort study.** | | |
| --- | --- | --- |
| **Protocol components** | **Key SUSTAIN 7 trial protocol components** (a randomized clinical trial investigating the efficacy and safety of semaglutide versus dulaglutide in adults with type 2 diabetes) | **Active-comparator, new-user cohort study protocol components implemented using a UK primary care electronic medical record database** (i.e., the IQVIA Medical Research Data (IMRD) incorporating data from THIN, a Cegedim Database) |
| **Objectives** | 1. **Primary objective:** To compare the effect of once-weekly dosing of two dose levels of subcutaneous semaglutide (0.5 mg and 1.0 mg) versus once-weekly dosing of two dose levels of subcutaneous dulaglutide (0.75 mg and 1.5 mg) on glycaemic control in subjects with type 2 diabetes on a background treatment with metformin. 2. **Secondary objective:** To compare the effect of once-weekly dosing of two dose levels of subcutaneous semaglutide (0.5 mg and 1.0 mg) versus once-weekly dosing of two dose levels of subcutaneous dulaglutide (0.75 mg and 1.5 mg) in subjects with type 2 diabetes on a background treatment with metformin with regards to body weight control, blood pressure, patient reported outcomes, and safety and tolerability. | 1. **Primary objective:** To estimate the comparative effectiveness of subcutaneous semaglutide versus subcutaneous dulaglutide on glycemic and bodyweight control among individuals with type 2 diabetes in routine UK clinical practice. 2. **Secondary objective:** To assess the effects of using subcutaneous semaglutide and subcutaneous dulaglutide in individuals with type 2 diabetes on additional clinical measures, including blood pressure, kidney function (eGFR), and safety profile. 3. **Exploratory analyses beyond the primary objective:** 4. To evaluate treatment effects across different subgroups defined by treatment persistence (see analysis set definitions). 5. To stratify analyses by eligibility criteria of the SUSTAIN 7,^1^ STEP 2,^2^ and SUSTAIN 6^3^ trials with different outcome priorities, to examine treatment effect heterogeneity among included and excluded individuals (see Supplementary Table S7 for corresponding eligibility criteria). 6. To assess heterogeneity of treatment effects across clinically relevant subgroups (age, sex, BMI, diabetes duration, baseline HbA1c, CVD history, and number of co-prescribed glucose-lowering therapies), reflecting subgroup definitions from the REWIND trial.^4^ 7. To assess the effects of semaglutide (0.5 mg and 1.0 mg) and dulaglutide (0.75 mg and 1.5 mg) at specific dose levels. |
| **Eligibility criteria** | Eligible patients were aged 18 years or older and had type 2 diabetes with HbA1c 7.0-10.5% on metformin monotherapy. Detailed eligibility criteria are outlined in Supplementary Table S7. | Eligible individuals were aged ≥18 years and initiated either subcutaneous semaglutide or subcutaneous dulaglutide as their first GLP-1RA between 01-Jan-2019 and 30-Jun-2023.  Additionally, to be eligible, individuals had a diagnosis of type 2 diabetes before or at the date of the first GLP-1RA prescription (cohort entry) at least one year of continuous registration with their GP practice prior to cohort entry and a minimum of 210-days of follow-up thereafter. We excluded individuals with diagnosed type 1 diabetes prior to or at cohort entry and those who received more than one GLP-1RA agent at cohort entry. |
| **Treatment strategies** | **Semaglutide treatment:** Subjects randomised to semaglutide will follow a fixed dose escalation. The maintenance dose of 0.5 mg will be reached after 4 doses (4 weeks) of 0.25 mg. The maintenance dose of 1.0 mg will be reached after 4 doses (4 weeks) of 0.25 mg, followed by 4 doses (4 weeks) of 0.5 mg. After the maintenance dose is reached, the dose must not be changed during the course of the trial unless due to safety reasons.  **Dulaglutide treatment:** Subjects randomised to dulaglutide will receive a dose of either 0.75 mg or 1.5 mg dulaglutide once-weekly without dose escalation. The randomisation dose must not be changed during the course of the trial unless due to safety reasons. | Individuals entered the respective new-user cohort at the date of the first prescription of subcutaneous semaglutide or dulaglutide. |
| **Treatment assignment** | Subjects with T2D inadequately controlled with metformin alone will after approximately 2 weeks screening period be randomised in a 1:1:1:1 manner to receive either a dose of 0.5 mg or 1.0 mg of semaglutide once-weekly or 0.75 mg or 1.5 mg of dulaglutide once-weekly. | To emulate randomization between treatment strategies, we used IPTWs based on covariates measured at cohort entry to adjust for confounding between individuals initiating subcutaneous semaglutide or dulaglutide in UK primary care. |
| **Analysis sets** | **Full analysis set (FAS):** Includes all randomised subjects exposed to at least one dose of trial product. Subjects in the FAS will contribute to the evaluation “as randomised”.  **Per-protocol analysis set:** Includes all subjects in the FAS who fulfil the following criteria:   - Have not violated any inclusion criteria - Have not fulfilled any exclusion criteria - Have a non-missing HbA1c measurement at screening and/or randomisation - Is on trial product at week 28 and have at least one non-missing HbA1c measurement at or after week 28   Subjects in the per-protocol analysis set will contribute to the analysis “as treated”. | **Full analysis set (FAS; secondary analysis set):** Included eligible individuals who received at least one prescription for either subcutaneous semaglutide or dulaglutide and met all inclusion and exclusion criteria at cohort entry (see **Figure 1** for detailed study cohort assembly).  **Per-protocol analysis set (primary analysis set; the persistent users):** Included all individuals in the FAS who remained on their assigned treatment strategy for more than 210-days (an observational analogue to 28-weeks, also considering the 6-month NICE treatment response review period (180 days) plus a 30-day buffer for delayed outcome recording).^5^  **Early-attrition analysis set (secondary analysis set):** Included all individuals in the FAS who discontinued their assigned treatment strategy before or at 210-days. |
| **Study design, follow-up, and observation periods (i.e., data point selection)** | The trial is a 40-week randomised, open-label, active-controlled, parallel group, multicentre, multinational, four-armed trial. After the treatment period of approximately 40 weeks in total, all subjects enter a follow-up period of 5 weeks ended by a follow-up phone contact. Total trial duration for the individual subjects will be approximately 47 weeks.   - The **“in-trial” observation period** includes observations recorded at or after randomization and not after the last subject-investigator contact, which is the follow-up phone contact 5 weeks after planned last dose of trial product. For subjects who withdraw their informed consent, the “in-trial” observation period ends at their date of withdrawal. In the case a subject dies during the trial, the date of death will be the end-date of the “in-trial” observation period. If a subject is lost to follow-up, the end of his/her “in-trial” period is defined as the date of the last subject investigator contact (site or phone visit). - The **primary observation period** **(on-treatment without rescue)** is a subset of the “in-trial” observation period and represents the time period where subjects are considered treated with trial product and do not receive any non-investigational antidiabetic medication (rescue medication).   Specifically it includes observations recorded at or after date of first dose of trial product and not after the first occurrence of the following:   - The last dose of trial product plus the dosing interval - Initiation of rescue medication | This UK population-based study used an active comparator, new-user cohort design.^6^ Follow-up began at cohort entry (i.e.., at initiation of injectable semaglutide or dulaglutide) and continued until the earliest occurrence of: death, transfer out of the GP practice, end of data availability (30 June 2023), or the end of the predefined study period (510-days after cohort entry). Individuals who switched between GLP-1RA agents did not re-enter the cohort after switching.   - The “**intention-to-treat” observation period** included observations assessed at or after cohort entry, but not after end of follow-up (i.e., regardless of treatment strategy persistence after cohort entry). - The **primary observation period** **(“on-treatment with the assigned strategy at cohort entry”)** is a subset of the “intention-to-treat” observation period and represents the time window where individuals were considered continuously treated with their treatment strategy assigned at cohort entry.   Specifically, this included observations recorded at or after cohort entry, but not after the occurrence of the following:   - The end of coverage of the last prescription for the assigned treatment strategy, if not followed by a subsequent prescription within 90-days (assuming a standard 30-day supply per prescription and a maximum permissible gap of 60-days) - Initiation of a GLP-1RA agent other than the one assigned at cohort entry - The **secondary observation period** **(“on-treatment with the assigned strategy at cohort entry up to 6-months”)** is a subset of the primary observation period. Specifically, this included all observations considered by the primary observation period that occurred before or at 210-days of follow-up. |
| **Outcome timing** | **Primary endpoint timing:** Changes in endpoint measurements at week 40 in relation to baseline measurements. | **Primary endpoint timing:** Changes in endpoint measurements over the one-year following treatment strategy initiation in relation to measurements at cohort entry (see Supplementary Table S3 for outcome definition details); using data from the primary observation period.  **Secondary endpoint timing:** Changes in endpoint measurements in relation to measurements at cohort entry and safety profiles over the 6-months following treatment strategy initiation (see Supplementary Table S3 for outcome definition details); using data from the secondary observation period. |
| **Outcome assessment** | 1. **Primary endpoint:** Change from baseline to week 40 in HbA1c 2. **Secondary endpoints:**    1. **Confirmatory secondary endpoint:** Change from baseline to week 40 in body weight (kg)    2. **Supportive secondary endpoints measured at 40 weeks** (relevant selected by authors of this study):       1. Change from baseline in BMI       2. Systolic and diastolic blood pressure       3. HbA1c reduction ≥1% (yes/no)       4. Weight loss ≥3% (yes/no)       5. Weight loss ≥5% (yes/no)       6. Weight loss ≥10% (yes/no)       7. HbA1c reduction ≥1% and weight loss ≥3% (yes/no) | 1. **Primary endpoint:** Absolute and relative changes from HbA1c and bodyweight at cohort entry 2. **Secondary endpoints:**    - 1. Absolute change from BMI at cohort entry      2. Absolute changes systolic and diastolic blood pressure      3. Absolute change from eGFR at cohort entry      4. Absolute HbA1c reduction ≥1% (yes/no)      5. Percentage weight loss ≥3% (yes/no)      6. Percentage weight loss ≥5% (yes/no)      7. Percentage weight loss ≥10% (yes/no)      8. Absolute HbA1c reduction ≥1% and percentage weight loss ≥3% (yes/no) |
| **Causal contrast** | **The two principal comparisons presented from a statistical analysis are:**   - s.c. semaglutide 0.5 mg versus dulaglutide 0.75 mg - s.c. semaglutide 1.0 mg versus dulaglutide 1.5 mg   The **primary estimand** will be: de-jure treatment difference at week 40 for all randomised subjects if all subjects adhered to treatment and did not initiate antidiabetic rescue medication. | **The comparisons made were:**   - **Primary contrast:** Subcutaneous semaglutide versus subcutaneous dulaglutide - **Exploratory contrasts:** Treatment effect heterogeneity across pre-defined clinical subgroups and effects of semaglutide (0.5 mg and 1.0 mg) and dulaglutide (0.75 mg and 1.5 mg) at specific dose levels.   The **primary estimand** was: the while-on-treatment average treatment effect (ATE), defined as the comparative effectiveness of initiating semaglutide versus dulaglutide among patients who remained on-treatment. |
| **Statistical analysis plan** | The primary analysis used to estimate the primary estimand will be based on FAS using data from the on-treatment without rescue observation period in a Mixed Model for Repeated Measures (MMRM). A restricted maximum likelihood (REML) will be used. The model will include all post baseline HbA1c measurements collected at scheduled visits up to and including week 40 data as dependent variables. The independent effects included in the model will be treatment and country as fixed effects and baseline response as covariate, all nested within visit. An unstructured covariance matrix will be employed for measurements within the same subject, assuming that measurements across subjects are independent. From this model, the two by dose level estimated treatment differences between s.c. semaglutide versus dulaglutide at week 40 will be presented together with associated two-sided 95% confidence intervals and p-values corresponding to two-sided tests of no difference if not otherwise specified. | The primary analysis used to estimate the primary estimand was based on the per-protocol analysis set using data from the primary (“on-treatment with the assigned strategy at cohort entry”) observation period.  To estimate treatment effects while adjusting for baseline confounding, we used MSMs with stabilized IPTWs based on logistic regression-derived propensity scores.  To address missing data in continuous baseline covariates and longitudinal outcomes, we used multiple imputation^7^ by chained equations with 100 imputed datasets,^8,9^ assuming data were missing at random.^10^ Propensity scores and outcome models were estimated separately in each imputed dataset, while treatment effect estimates were combined using Rubin’s rules, yielding pooled estimates and associated 95% confidence intervals.^7^  For continuous outcomes (e.g., percentage change in HbA1c or bodyweight), we fitted IPTW-weighted GLMs with an identity link function. For binary outcomes (e.g., achieving ≥1% absolute HbA1c reduction or ≥5% percentage weight loss), we estimated IPTW-weighted GLMs with a logit link. Robust standard errors (sandwich estimators) were used. |
| **Missing data considerations** | Since both semaglutide and dulaglutide are GLP-1 RA, it is reasonable to assume that missing data in both arms will be similar in timing, extent and reason. The MMRM is a well-established method that accounts for the uncertainty pertaining to missing data. This analysis assumes that the missing data mechanism is missing at random (MAR). Thus, for a subject who has missing data, MAR assumes a value for the endpoint based on observed data of subjects whose baseline explanatory variables and response up to withdrawal are similar to that of the discontinued subject.  For subjects who have no post-baseline scheduled assessments available in the on-treatment without rescue period, the baseline value will be carried forward to the first scheduled visit for the associated endpoint to ensure that all randomised subjects will contribute to the statistical analysis.  Descriptive summaries and graphical representation of extent, reason(s) for and pattern of missing data will be presented by treatment arm. | Missing baseline and endpoint measurements were handled using multiple imputation by chained equations, under a missing-at-random assumption.^7–10^ Imputation models included all baseline covariates, treatment group, and outcome variables, as well as auxiliary variables considered to be associated with missingness (see Supplementary Table S5).^10^ The extent and distribution of missing data were summarized descriptively (see Supplementary Table S6), and results were compared with complete case analyses to evaluate sensitivity to imputation assumptions. |
| **Sensitivity analyses** | **Per-protocol analysis** - the statistical analysis will be the same as the primary MMRM based analysis but it will be based on the per-protocol analysis set and the on-treatment without rescue observation period.  **Complete case analysis** - includes subjects in the FAS who do not have their endpoint imputed in the primary analysis. The change from baseline in HbA1c at week 40 will be analysed by a linear normal model (analysis of covariance (ANCOVA)) with treatment and country as fixed effects and baseline HbA1c as a covariate. | Sensitivity analyses were performed on the primary outcomes (i.e., changes in HbA1c and bodyweight at 1-year; see Supplementary Table S3) among those in the primary (i.e., per-protocol) analysis set. Within the per-protocol analysis set, using data from the primary (“on-treatment with the assigned strategy at cohort entry”) observation period, we repeated the primary analysis:   - Using stabilized IPTWs trimmed at the 1st and 99th percentiles to reduce the influence of extreme weights. - Without using IPTWs to assess robustness to removal of weighting. - Using joint weights calculated as the product of stabilized IPTWs and IPCWs, with IPCWs estimated from the conditional probabilities of censoring to assess the impact of informative censoring.   Additionally, we defined a **complete case analysis set**, which included all individuals in the per-protocol analysis set who: (1) had a non-missing baseline endpoint measurement (i.e., HbA1c or bodyweight, respectively) at cohort entry; and (2) had at least one non-missing post-baseline endpoint measurement (i.e., HbA1c or bodyweight, respectively). Within the complete case analysis set, we repeated the primary analysis (for continuous baseline covariates with missing values; we used the categories specified in Supplementary Table S4, including an included an "unknown" category):   - Using data from the primary (“on-treatment with the assigned strategy at cohort entry”) observation period. - Using data from the “intention-to-treat” observation period. - Using propensity score matching (nearest-neighbor matching using a 1:1 ratio, with a caliper width of 0.2 standard deviations on the logit of the propensity score, and without replacement). Propensity scores were estimated using the same logistic regression model as in the primary inverse probability of treatment weighting analysis using data from the primary (“on-treatment with the assigned strategy at cohort entry”) observation period. - Changing the primary endpoint assessment window to progressively narrower windows of ±90, ±60, and ±30 days around the primary 1-year (365-days) outcome timing using data from the primary (“on-treatment with the assigned strategy at cohort entry”) observation period. - Varying the primary definition of permissible gap between consecutive prescriptions from 90-days to: 60, 120, and 180-days using data from the primary (“on-treatment with the assigned strategy at cohort entry”) observation period. |
| Abbreviations: SUSTAIN Semaglutide Unabated Sustainability in Treatment of Type 2 Diabetes, UK United Kingdom, eGFR estimated glomerular filtration rate, STEP Semaglutide Treatment Effect in People With Obesity, BMI body-mass index, HbA1c glycated hemoglobin, CVD cardiovascular disease, REWIND Researching cardiovascular Events with a Weekly INcretin in Diabetes, GLP-1RA glucagon-like peptide-1 receptor agonist, GP general practitioner, IPTW inverse probability of treatment weight, FAS full analysis set, ATE average treatment effect, MSM marginal structural model, GLM generalized linear model, IPCWs inverse probability of censoring weights. | | |

| **Table S2: ATC codes and generic names to define usage and dosage of once-weekly injectable semaglutide and dulaglutide.** | | | |
| --- | --- | --- | --- |
| **GLP-1 receptor agonist agent**  **(ATC code)** | **Generic name** | **Mode of administration: subcutaneous injection (sc) or oral tablet (po)** | **Dose per administration*** |
| **Semaglutide (A10BJ06)** | Ozempic 1mg/0.74ml solution for injection 3ml pre-filled pen | sc | 1mg |
|  | Semaglutide 1mg/0.74ml solution for injection 3ml pre-filled disposable device | sc | 1mg |
|  | Ozempic 0.5mg/0.37ml solution for injection 1.5ml pre-filled pen | sc | 0.5mg |
|  | Semaglutide 0.5mg/0.37ml solution for injection 1.5ml pre-filled disposable device | sc | 0.5mg |
|  | Ozempic 0.25mg/0.19ml solution for injection 1.5ml pre-filled pen | sc | 0.25mg |
|  | Semaglutide 0.25mg/0.19ml solution for injection 1.5ml pre-filled disposable device | sc | 0.25mg |
| **Dulaglutide (A10BJ05)** | Trulicity 3mg/0.5ml solution for injection pre-filled pens | sc | 3mg |
|  | Dulaglutide 3mg/0.5ml solution for injection pre-filled disposable devices | sc | 3mg |
|  | Trulicity 4.5mg/0.5ml solution for injection pre-filled pens | sc | 4.5mg |
|  | Dulaglutide 4.5mg/0.5ml solution for injection pre-filled disposable devices | sc | 4.5mg |
|  | Dulaglutide 1.5mg/0.5ml solution for injection pre-filled disposable devices | sc | 1.5mg |
|  | Dulaglutide 0.75mg/0.5ml solution for injection pre-filled disposable devices | sc | 0.5mg |
| *Dose per administration is based on the information provided by the generic name. | | | |

| **Table S3: Outcome definitions and timing.** | | | |
| --- | --- | --- | --- |
| **Type of outcome** | **Outcome** | **Primary endpoint timing (1-year) by analysis set^c^** | **Secondary endpoint timing (6-months) by analysis set^c^** |
| **Continuous** | **Primary endpoints:**   - Absolute (percentage points) and percentage (%) change in HbA1c (measured in %) from cohort entry - Absolute (kg) and percentage (%) change in bodyweight (measured in kg) from cohort entry   **Secondary endpoints:**   - Absolute change (kg/m^2^) in BMI (measured in kg/m^2^) from cohort entry; calculated from the corresponding weight outcome measurement and the most recent height measurement captured prior to (when ≥18 years old) or at the index date - Absolute change (mmHg) in systolic blood pressure (measured in mmHg) from cohort entry - Absolute change (mmHg) in diastolic blood pressure (measured in mmHg) from cohort entry - Absolute change ml/min/1.73m^2^) in eGFR (measured in ml/min/1.73m^2^ and calculated from serum creatinine using the 2021 CKD-EPI equation^11^) from cohort entry | **Per-protocol (i.e., primary) analysis set:**  We used the last on-treatment endpoint measurement closest to 510 days, accepting values between 211 and 510 days to account for the irregular timing of clinical assessments in real-world clinical practice.  If no measurement was available within this period, the outcome was designated as missing and imputed according to Supplementary Table S5.  This window is supported by evidence from the SUSTAIN 7 trial^1^ indicating that most of the treatment effect of injectable semaglutide and dulaglutide occurs within the first 28-weeks, with minimal additional change observable thereafter. Consequently, carrying forward the last available on-treatment measurement within this period is unlikely to introduce meaningful bias and thus, provides a valid approach for assessing the 1-year outcome using real-world data.  **Secondary analysis sets:**  The **full analysis set** comprised all individuals in both the per-protocol and early-attrition analysis sets. By definition, individuals in the **early-attrition set** could not contribute to the 1-year primary endpoint timing defined for the per-protocol set; therefore, the population-level on-treatment effect of injectable semaglutide versus dulaglutide in the full analysis set reflects the aggregated effect of the 1-year outcome from the per-protocol set and the 6-month outcome from the early-attrition set. | **Per-protocol (i.e., primary) analysis set:**  We used the measurement closest to 210 days, accepting values between 1 and 210 days to account for the irregular timing of clinical assessments in real-world clinical practice.  If no measurement was available within this period, the outcome was designated as missing and imputed according to Supplementary Table S5.  This window aligns with UK NICE guidance for type 2 diabetes management, which recommends discontinuing GLP-1RA therapy after 6-months (approximately 180-days) if a composite target of absolute HbA1c reduction ≥1%^a^ and percentage weight loss ≥3%^b^ is not achieved,^5^ while allowing an additional 30-days to capture delayed follow-up assessments.  Additionally, evidence from the SUSTAIN 7 trial^1^ indicates that the treatment effects of injectable semaglutide and dulaglutide occur rapidly and approach their maximum within the first 28-weeks.  **Secondary analysis sets:**  The same 6-month endpoint timing definition was used for both the **full analysis set** and the **early-attrition analysis set**. |
| **Binary** | **Secondary endpoints measured as proportion of individuals achieving glycemic and weight management targets at the respective outcome assessment timepoint:**   - HbA1c reduction ≥1%^a^ - Weight loss ≥3%^b^ - Weight loss ≥5%^b^ - Weight loss ≥10%^b^ - Composite of HbA1c reduction ≥1%^a^ and bodyweight reduction ≥3%^b^ |  |  |
| Abbreviations: HbA1c glycated hemoglobin, BMI body-mass index, eGFR estimated glomerular filtration rate, CKD-EPI Chronic Kidney Disease Epidemiology Collaboration, UK United Kingdom, NICE National Institute for Health and Care Excellence. ^a^Based on absolute change in HbA1c (percentage points). ^b^Based on percentage change in bodyweight (%). ^c^The full analysis set included eligible individuals who received at least one prescription for injectable semaglutide or dulaglutide. The per-protocol (i.e., primary) analysis set (an observational analogue to the SUSTAIN 7 trial per-protocol analysis set)^1^ included individuals from the full analysis set who remained on their assigned treatment strategy for more than 210 days after cohort entry. All individuals in the full analysis set who were ineligible for the per-protocol analysis set (i.e., those who remained on their assigned treatment strategy for 210 days or less) were added to the early-attrition analysis set. See details on analysis set definitions and observation periods in Supplementary Table S1 and Figure 1. | | | |

| **Table S4: Covariate definitions and assessment periods.** | | | |
| --- | --- | --- | --- |
| **Covariate**  **(type)** | **Covariate assessment timing (time-fixed^a^ or time-varying^b^)** | **Details** | **Analysis covariate^c^** |
| Age  (continuous)  (categorical, levels: 18-34, 35-49, 50-59, 60-60, ≥70) | Time-varying | Time in years between covariate assessment date and year of birth | x |
| Sex  (binary, levels: female, male) | Time-fixed | Sex assigned at birth | x |
| Smoking status  (categorical, levels: current, former, never, unknown) | Time-varying | Most recent clinical code for smoking status recorded before or at the covariate assessment date. | x |
| Alcohol status  (categorical, levels: current, former, never, unknown) | Time-varying | Most recent clinical code for smoking status recorded before or at the covariate assessment date. | x |
| Calendar year at cohort entry  (categorical, levels: 2019, 2020, 2021, 2022) | Time-fixed | Calendar year at the date of the first prescription for injectable semaglutide or dulaglutide | x |
| Type 2 diabetes duration  (continuous)  (categorical, levels: <5, 5-<10, ≥10) | Time-varying | Time in years between the first diagnosis code (Read code) for type 2 diabetes and the covariate assessment date | x |
| Co-prescribed glucose-lowering therapies  (binary, levels: drug classes specified below used yes/no)   - - Metformin   - Sulfonylureas   - Sodium-glucose co-transporter-2 inhibitors   - Dipeptidyl peptidase-4 inhibitors   - Thiazolidinediones   - Insulin   - Other glucose-lowering drugs   (categorical, levels: number of co-prescribed glucose-lowering therapies being 0,1, 2, or ≥3) | Time-varying | At least one prescription within the drug class of interest within the 180-days prior to or at the covariate assessment date | x |
| Comedications   - Diuretics - Beta-blockers - Calcium channel blockers - Angiotensin converting enzyme inhibitors - Angiotensin II receptor blockers - Acetylsalicylic acid - Other antiplatelets - Anticoagulants - Statins - Antipsychotics - Selective serotonin reuptake inhibitor - Tricyclic antidepressants - Other antidepressants - Anxiolytics - Antiepileptics - Gabapentinoids - Opioids - Nonsteroidal anti-inflammatory drugs - Proton-pump inhibitors - Anti-obesity medications - Systemic corticosteroids - Thyroid hormone replacements | Time-varying | At least one prescription within the drug class of interest within the 180-days prior to or at the covariate assessment date | x |
| Comorbidities  assessment definition 1  (binary, levels: yes/no):   - Cardiovascular disease - Cardiovascular event (myocardial infarction, stroke) - Stroke - Myocardial infarction - Unstable angina - Coronary, carotid or peripheral arterial revascularization - Stenosis of coronary or carotid arteries, or lower extremity arteries - Heart failure - Hypertension - Dyslipidemia - Obesity treatment with surgery or a weight loss device - Asthma - Chronic obstructive pulmonary disease - Sleep apnea - Osteoarthritis - Non-alcoholic fatty liver disease - Pancreatitis - Gallbladder disorders - Hyperthyroidism - Hypothyroidism - Chronic kidney disease - Retinopathy - Neuropathy - Polycystic ovarian syndrome - Gestational diabetes - Depression - Cancer   Comorbidities  assessment definition 2  (binary, levels: yes/no):   - Drug abuse   Comorbidities  assessment definition 3  (binary, levels: yes/no):   - Gastrointestinal disorders - Acute renal failure - Abdominoplasty/liposuction - Urinary tract infection - Hypoglycemia - Bone fracture - Pregnancy - Diabetic ketoacidosis | Time-varying | Comorbidities  assessment definition 1:  Diagnosis code for comorbidity of interest (Read code) prior to or at the covariate assessment date  Comorbidities  assessment definition 2:  Diagnosis code for comorbidity of interest (Read code) within the 2-years prior to or at the covariate assessment date  Comorbidities  assessment definition 3:  Diagnosis code for comorbidity of interest (Read code) within the 1-year prior to or at the covariate assessment date | x  (except for drug abuse, pregnancy, diabetic ketoacidosis, abdominoplasty or liposuction) |
| HbA1c (%)  (continuous)  (categorical, levels: <8, ≥8) | Time-varying | Most recent HbA1c laboratory test recorded within 540 days^d^ prior to or at the covariate assessment date. | x |
| Bodyweight (kg)  (continuous) | Time-varying | Most recent weight measurement recorded within 540 days^d^ prior to or at the covariate assessment date. | x |
| BMI (kg/m^2^)  (continuous)  (categorical, levels: ≥40, ≥35-<40, ≥30-<35, ≥25<30, <25) | Time-varying | BMI derived from most recent weight measurement recorded within 540 days^d^ prior to or at the covariate assessment date and most recent height measurement captured prior to (when ≥18 years old) or at the covariate assessment date. | x |
| Systolic blood pressure (mmHg)  (continuous)  (categorical, levels: ≥140, <140) | Time-varying | Most recent systolic blood pressure measurement captured within 540 days^d^ prior to or at the covariate assessment date. | x |
| Diastolic blood pressure (mmHg)  (continuous)  (categorical, levels: ≥90, <90) | Time-varying | Most recent diastolic blood pressure measurement captured within 540 days^d^ prior to or at the covariate assessment date. | x |
| eGFR (ml/min/1.73m^2^)  (continuous)  (categorical, levels: ≥90, ≥60-<90, ≥30-<60, <30) | Time-varying | eGFR derived from most recent serum creatinine laboratory test recorded within 540 days^d^ prior to or at the covariate assessment date using the 2021 Chronic Kidney Disease Epidemiology Collaboration (CKD-EPI) equation.^11^ | x |
| ^a^Time-fixed covariates were assessed at study cohort entry. ^b^Time-varying covariates were assessed at study cohort entry and updated every 30-days. ^c^Analysis covariates were used for conducting the multiple imputation (time-fixed and time-varying covariates measured at cohort entry; see Supplementary Table S5), calculating the inverse probability of treatment weights (time-fixed and time-varying covariates measured at cohort entry), and for calculating the inverse probability of censoring weights (time-fixed covariates measured at cohort entry and time-varying covariates updated throughout follow-up). ^d^This timeframe was chosen based on the Quality Outcomes Framework recommendations that advise annual clinical assessments for individuals with diabetes, allowing extra time for scheduling visits and recording information.^12–14^ Abbreviations: HbA1c glycated hemoglobin, BMI body-mass index, eGFR estimated glomerular filtration rate. | | | |

| **Table S5: Imputed variables and covariates included in imputation models.** | |
| --- | --- |
| **Imputed partially observed variables^a^** | **Predictors included in each imputation model (specified separately by treatment strategy)** |
| **Missing measurements in continuous baseline covariates** | |
| HbA1c | - Analysis covariates (specified in Supplementary Table S4) - Baseline measurements of continuous variables - Outcomes assessed over 1-year - Outcomes assessed over 6-months - Additional time-varying auxiliary variables:   - - 1. Indicator of inclusion in the per-protocol analysis set (coded ‘no’ for early-attrition participants       2. Censoring indicator per 30-day interval during follow-up (yes/no)       3. Treatment strategy discontinuation indicator per 30-day interval during follow-up (yes/no)       4. Highest dose prescribed per 30-day interval during follow-up (Supplementary Table S2 for dose information) |
| Weight/BMI |  |
| Systolic BP |  |
| Diastolic BP |  |
| eGFR |  |
| **Missing measurements in outcomes assessed at 6-months/1-year^b^** | |
| HbA1c | - Analysis covariates (specified in Supplementary Table S4) - Baseline measurements of continuous variables - Outcomes assessed over 1-year - Outcomes assessed over 6-months - Additional time-varying auxiliary variables:  1. Indicator of inclusion in the per-protocol analysis set (coded ‘no’ for early-attrition participants 2. Censoring indicator per 30-day interval during follow-up (yes/no) 3. Treatment strategy discontinuation indicator per 30-day interval during follow-up (yes/no) 4. Highest dose prescribed per 30-day interval during follow-up (Supplementary Table S2 for dose information) |
| Weight/BMI |  |
| Systolic BP |  |
| Diastolic BP |  |
| eGFR |  |
| Abbreviations: HbA1c glycated hemoglobin, BMI body-mass index, BP blood pressure, eGFR estimated glomerular filtration rate, NA not applicable. ^a^Summary of missing data in continuous baseline covariates and longitudinal outcome measures is presented in Supplementary Table S6. ^b^By definition, individuals in the early-attrition set could not contribute to the 1-year primary endpoint timing defined for the per-protocol set (Supplementary Table S3); therefore, the population-level on-treatment effect of injectable semaglutide versus dulaglutide in the full analysis set reflects the aggregated effect of the 1-year outcome from the per-protocol set and the 6-month outcome from the early-attrition set. | |

| **Table S6: Descriptive summary of missing data in continuous baseline covariates and longitudinal outcomes, by analysis set and treatment strategy.** | | | | | | |
| --- | --- | --- | --- | --- | --- | --- |
| **Analysis set** | **Primary** | | **Secondary** | | | |
|  | **Per-protocol analysis set** | | **Full analysis set** | | **Early-attrition analysis set** | |
| **Treatment strategy** | **Semaglutide**  **(n = 1901)** | **Dulaglutide**  **(n = 2735)** | **Semaglutide**  **(n = 2918)** | **Dulaglutide**  **(n = 3698)** | **Semaglutide**  **(n = 1017)** | **Dulaglutide**  **(n = 963)** |
| **Missing measurements in continuous baseline covariates** | | | | | | |
| HbA1c | 539 (28.4) | 854 (31.2) | 854 (29.3) | 1179 (31.9) | 315 (31.0) | 325 (33.7) |
| Weight/BMI | 403 (21.2) | 540 (19.7) | 629 (21.6) | 746 (20.2) | 226 (22.2) | 206 (21.4) |
| Systolic BP | 97 (5.1) | 118 (4.3) | 162 (5.6) | 169 (4.6) | 65 (6.4) | 51 (5.3) |
| Diastolic BP | 97 (5.1) | 118 (4.3) | 162 (5.6) | 169 (4.6) | 65 (6.4) | 51 (5.3) |
| eGFR | 20 (1.1) | 23 (0.8) | 47 (1.6) | 41 (1.1) | 27 (2.7) | 18 (1.9) |
| **Missing measurements in outcomes assessed at 1-year** | | | | | | |
| HbA1c | 803 (42.2) | 1273 (46.5) | 1580 (54.1) | 2005 (54.2) | NA | NA |
| Weight/BMI | 1027 (54.0) | 1429 (52.2) | 1777 (60.9) | 2170 (58.7) | NA | NA |
| Systolic BP | 811 (42.7) | 1082 (39.6) | 1540 (52.8) | 1765 (47.7) | NA | NA |
| Diastolic BP | 811 (42.7) | 1082 (39.6) | 1540 (52.8) | 1765 (47.7) | NA | NA |
| eGFR | 638 (33.6) | 878 (32.1) | 1321 (45.3) | 1499 (40.5) | NA | NA |
| **Missing measurements in outcomes assessed at 6-months** | | | | | | |
| HbA1c | 679 (35.7) | 1043 (38.1) | 1456 (49.9) | 1775 (48.0) | 777 (76.4) | 732 (76.0) |
| Weight/BMI | 907 (47.7) | 1375 (50.3) | 1657 (56.8) | 2116 (57.2) | 750 (73.7) | 741 (76.9) |
| Systolic BP | 785 (41.3) | 1104 (40.4) | 1514 (51.9) | 1787 (48.3) | 729 (71.7) | 683 (70.9) |
| Diastolic BP | 785 (41.3) | 1104 (40.4) | 1514 (51.9) | 1787 (48.3) | 729 (71.7) | 683 (70.9) |
| eGFR | 583 (30.7) | 925 (33.8) | 1266 (43.4) | 1546 (41.8) | 683 (67.2) | 621 (64.5) |
| Data presented in n (%). Abbreviations: HbA1c glycated hemoglobin, BMI body-mass index, BP blood pressure, eGFR estimated glomerular filtration rate, NA not applicable. *By definition, individuals in the early-attrition set could not contribute to the 1-year primary endpoint timing defined for the per-protocol set (NA); therefore, the population-level on-treatment effect of injectable semaglutide versus dulaglutide in the full analysis set reflects the aggregated effect of the 1-year outcome from the per-protocol set and the 6-month outcome from the early-attrition set. | | | | | | |

| **Table S7: Application of SUSTAIN 7,**^1^ **STEP 2,**^2^ **and SUSTAIN 6**^3^ **trial eligibility criteria to UK primary care data.** | | | | |
| --- | --- | --- | --- | --- |
| **Eligibility criteria for respective type 2 diabetes trial populations** | **Emulation of trial eligibility criteria** | **SUSTAIN 7 trial**^1^ | **STEP 2 trial**^2^ | **SUSTAIN 6 trial**^3^ |
| **Treatment comparison** | Once-weekly subcutaneous semaglutide versus dulaglutide | Once-weekly subcutaneous semaglutide 0.5 mg versus dulaglutide 0.75 mg and  semaglutide 1.0 mg versus dulaglutide 1.5 mg | Once-weekly subcutaneous semaglutide 2.4 mg versus semaglutide 1.0 mg (semaglutide 1.0 mg versus placebo) | Once-weekly subcutaneous semaglutide 0.5 mg or 1.0 mg versus placebo |
| **Scope** | Glycemic control, weight management, cardio-renal outcome measures | Glycemic control | Weight management | Cardiovascular outcomes trial |
| ***Inclusion criteria*** | | | | |
| **Informed consent obtained before any trial-related activities.** | Ethical approval for the use of the IMRD is issued by the NHS Health Research Authority (East Midlands - Derby Research Ethics Committee reference: 23/EM/0151).^15^ | Applied | Applied | Applied |
| **Male or female** | Sex assigned at birth (male or female) | Male or female | Male or female | Male or female |
| **Age** | ≥18 years | ≥18 years | ≥18 years | ≥50 years |
| *Type 2 diabetes-related inclusion criteria* | | | | |
| **Type 2 diabetes diagnosis** | Type 2 diabetes diagnosis before or at GLP-1RA initiation. | ≥90 days prior to screening | ≥180 days prior to screening | Type 2 diagnosis |
| **HbA1c** | HbA1c at baseline according to: SUSTAIN 7 trial: 7-10.5%  STEP 2 trial: 7-10%, SUSTAIN 6 trial: ≥7.0% | 7-10.5% | 7-10% | ≥7.0% at screening |
| **Medications for the indication of type 2 diabetes** | SUSTAIN 7 trial: ≥1 metformin prescription during the 90-days before or at GLP-1RA initiation, STEP 2 trial: Prescriptions for 0 up to 3 glucose-lowering agents, including metformin, sulfonylureas, SGLT-2i, or glitazone within the 90-days before or at GLP-1RA initiation, SUSTAIN 6 trial: Prescriptions for 0 up to 2 oral glucose-lowering agents, and insulin | Subjects on stable diabetes treatment with metformin for 90 days prior to screening | Patient treated with either: Diet and exercise alone or stable treatment with metformin, sulfonylurea (SU), sodium-glucose co-transporter-2 inhibitors (SGLT2is), or glitazone as single-agent therapy; or  Up to 3 oral glucose-lowering drugs (metformin, SU, SGLT2i, or glitazone) according to local label. Any approved and marketed metformin, glitazone, SGLT2i, or SU product, or combination products are allowed. Treatment with oral agents should be stable (same drug[s], dose, and dosing frequency) for at least 90 days prior to screening. | Anti-diabetic drug naïve, or treated with one or two OAD(s), or treated with human NPH insulin or long-acting insulin analogue or pre-mixed insulin, alone or in combination with one or two OAD(s). |
| *Obesity-related inclusion criteria (specific for the weight management trial, the STEP 2 trial)* | | | | |
| **BMI** | STEP 2 trial: Baseline BMI measurement ≥27 kg/m^2^ | NA | ≥27 kg/m^2^ | NA |
| **Unsuccessful lifestyle intervention** | According to the NICE guidelines, patients with type 2 diabetes should receive ongoing advice about diet and lifestyle.^5^ Thus, patients with overweight or obesity, and type 2 diabetes who initiated GLP-1RA therapy are likely to have a history of at least one unsuccessful dietary attempt. | NA | History of at least one self-reported unsuccessful dietary effort to lose body weight. | NA |
| *CVD-related inclusion criteria (specific for the CVOT, the SUSTAIN 6 trial)* | | | | |
| **CVD** | SUSTAIN 6 trial: Age ≥50 years at GLP-1RA initiation and having a history of CVD defined as having a diagnosis code for at least one of below criteria prior to or at GLP-1RA initiation: i. MI ii. Stroke or TIA iii. Prior coronary, carotid or peripheral arterial revascularization iv. Stenosis of coronary or carotid arteries, or lower extremity arteries v. Unstable angina vi. Heart failure vii. Chronic renal impairment, defined as baseline eGFR <60 mL/min/1.73m^2^ | NA | NA | Age ≥50 years at screening and clinical evidence of cardiovascular disease defined as meeting at least one of the below criteria (a - h). All applicable categories included in this criterion must be ticked off in the eCRF a. prior myocardial infarction b. prior stroke or transient ischaemic attack (TIA) c. prior coronary, carotid or peripheral arterial revascularization d. >50% stenosis on angiography or imaging of coronary, carotid or lower extremity arteries e. history of symptomatic coronary heart disease documented by positive exercise stress test or any cardiac imaging or unstable angina with ECG changes f. asymptomatic cardiac ischemia documented by positive nuclear imaging test or exercise test or dobutamine stress echo g. chronic heart failure New York Heart Association (NYHA) class II-III h. chronic renal impairment, defined as estimated glomerular filtration rate (eGFR) <60 mL/min/1.73m2 per MDRD  OR  Age ≥60 years at screening and subclinical evidence of cardiovascular disease defined as meeting at least one of the below criteria (i - l). All applicable categories included in this criterion must be ticked off in the eCRF i. persistent microalbuminuria (30-299 mg/g) or proteinuria j. hypertension and left ventricular hypertrophy by ECG or imaging  k. left ventricular systolic or diastolic dysfunction by imaging l. ankle/brachial index <0.9 |
| ***Exclusion criteria*** | | | | |
| *Trial-related exclusion criteria* | | | | |
| **Hypersensitivity to trial product** | NA | Known or suspected hypersensitivity to trial product(s) or related products | Known or suspected hypersensitivity to trial product(s) or related products | Known or suspected hypersensitivity to trial products or related products. |
| **Previous participation in the trial** | NA | Previous participation in this trial. Participation is defined as signed informed consent | Previous participation in this trial. Participation is defined as signed informed consent. Participation in another clinical trial within 90 days before screening. Other subject(s) from the same household participating in any semaglutide trial. | Simultaneous participation in any other clinical trial of an investigational agent. Participation in a clinical trial with investigational stent(s) is allowed. Previous participation in this trial. Participation is defined as randomised. |
| **Receipt of any investigational medicinal products** | NA | Receipt of any investigational medicinal product within 90 days before screening | Receipt of any other glucose-lowering investigational drug within 90 days prior to screening for this trial, or receipt of any investigational drugs not affecting diabetes within 30 days before screening for this trial. Treatment with a glucagon-like peptide-1 receptor agonist within 180 days prior to screening. | Receipt of any investigational medicinal product (IMP) within 30 days prior to screening (Visit 1) or according to local requirements, if longer. |
| **Any condition, which in the investigator's opinion might jeopardize subject's safety or compliance with the protocol** | NA | Any condition, which in the investigator's opinion might jeopardize subject's safety or compliance with the protocol | Any disorder, unwillingness or inability, not covered by any of the other exclusion criteria, which in the investigator’s opinion, might jeopardize the subject’s safety or compliance with the protocol. | Any acute condition or exacerbation of chronic condition that would in the investigator's opinion interfere with the initial trial visit schedule and procedures. |
| *Diabetes-related exclusion criteria* | | | | |
| **Type 1 diabetes** | Diagnosis code for type 1 diabetes before or at GLP-1RA initiation. | NA | NA | Type 1 diabetes |
| **Medications for the indication of type 2 diabetes or obesity (1)** | SUSTAIN 7 trial: Prescription of any medication with the indication of diabetes or obesity during the 90-days prior to GLP-1RA initiation apart from metformin (except for insulin)  STEP 2 trial: Prescriptions for any medications for the indication of type 2 diabetes or obesity other than stated in the inclusion criteria within the 90-days before GLP-1RA initiation. SUSTAIN 6 trial: Prescription for pramlintide within 90-days or prescription for DPP-4is within 30-days before or at GLP-1RA initiation. | Treatment with any medication for the indication of diabetes or obesity other than stated in the inclusion criteria in a period of 90-days before screening. An exception is short-term insulin treatment for acute illness for a total of <14-days | Treatment with any medication for the indication of diabetes or obesity other than stated in the inclusion criteria within the past 90 days before screening. | Use of GLP-1 receptor agonist (exenatide (BID or OW), liraglutide, or other) or pramlintide within 90 days prior to screening. Use of any DPP-IV inhibitor within 30 days prior to screening. Treatment with insulin other than basal and pre-mixed insulin, within 90 days prior to screening - except for short-term use in connection with intercurrent illness. Acute decompensation of glycaemic control requiring immediate intensification of treatment to prevent acute complications of diabetes (e.g. diabetes ketoacidosis) within 90 days prior to screening. |
| **Medications for the indication of type 2 diabetes or obesity (2)** | SUSTAIN 7 trial: Prescription for antiobesity medications, systemic corticosteroids, or thyroid hormones within the 90-days prior to GLP-1RA initiation. | Anticipated initiation or change in concomitant medications (for more than consecutive days or on a frequent basis) known to affect weight or glucose metabolism (e.g., orlistat, thyroid hormones, corticosteroids) | NA | NA |
| **Retinopathy/maculopathy** | SUSTAIN 7 trial & STEP 2 trial: Diagnosis code for retinopathy or maculopathy during the 90-days before or at GLP-1RA initiation. | Proliferative retinopathy or maculopathy requiring acute treatment | Uncontrolled and potentially unstable diabetic retinopathy or maculopathy, verified by a pharmacologically pupil-dilated fundus examination performed by an ophthalmologist or an equally qualified healthcare provider (e.g., optometrist) within the past 90 days before screening or in the period between screening and randomization. | NA |
| *Obesity-related exclusion criteria* | | | | |
| **Self-reported weight loss** | NA | NA | A self-reported change in body weight of >5 kg (11 lbs) within 90 days before screening, irrespective of medical records. | NA |
| **Obesity treatment with surgery or a weight loss device** | STEP 2 trial: Diagnosis code for liposuction and/or abdominoplasty during the 365 days before GLP-1RA initiation. Diagnosis code for obesity treatment with surgery or a weight loss device ever before or at GLP-1RA initiation. | NA | Previous or planned (during the trial period) obesity treatment with surgery or a weight loss device. However, the following are allowed: (1) liposuction and/or abdominoplasty, if performed > 1 year before screening, (2) lap banding, if the band has been removed > 1 year before screening, (3) intragastric balloon, if the balloon has been removed > 1 year before screening or (4) duodenal-jejunal bypass sleeve, if the sleeve has been removed > 1 year before screening | NA |
| **Uncontrolled thyroid disease** | STEP 2 trial: Uncontrolled thyroid disease, defined as thyroid-stimulating hormone >6 mlU/L or <0.4 mlU/L measured within the 90 days prior to baseline. | NA | Uncontrolled thyroid disease, defined as thyroid stimulating hormone (TSH) >6.0 mIU/L or <0.4 mIU/L as measured by central laboratory at screening | NA |
| *General safety* | | | | |
| **Pregnant, breastfeeding, or fertile women without contraception** | Diagnosis code for pregnancy or breastfeeding within the year prior to initiating GLP-1RA therapy. | Female who is pregnant, breast-feeding, or intends to become pregnant or is of child-bearing potential and not using an adequate contraceptive method (as required by local regulation or practice) | Female who is pregnant, breast-feeding or intends to become pregnant or is of child-bearing potential and not using a highly effective contraceptive method. | Female of childbearing potential who is pregnant, breast-feeding or intend to become pregnant or is not using adequate contraceptive methods (adequate contraceptive measures as required by local law or practice). |
| **Acute pancreatitis** | Diagnosis code for acute pancreatitis before or at GLP-1RA initiation | History of acute pancreatitis | Presence of acute pancreatitis within the past 180 days prior to the day of screening. | History of chronic pancreatitis or idiopathic acute pancreatitis. |
| **Chronic pancreatitis** | Diagnosis code for chronic pancreatitis before or at GLP-1RA initiation | History of chronic pancreatitis | History or presence of chronic pancreatitis. | History of chronic pancreatitis or idiopathic acute pancreatitis. |
| **Calcitonin** | Not available in the IMRD | Screening calcitonin >= 50ng/L | Calcitonin ≥ 100 ng/L as measured by the central laboratory at screening. | Screening calcitonin >= 50ng/L |
| **Cancer** | Diagnosis code for cancer (except for non-melanoma skin cancers) identified within the 5 years before or at GLP-1RA initiation. | History or presence of malignant neoplasms within the last 5 years (except basal and squamous cell skin cancer and in-situ carcinomas) | History of malignant neoplasms within the past 5 years prior to screening. Basal and squamous cell skin cancer and any carcinoma in-situ are allowed. | Malignant neoplasm requiring chemotherapy, surgery, radiation or palliative therapy in the previous 5 years. |
| **Multiple endocrine neoplasia type 2 or medullary thyroid carcinoma** | Diagnosis code for multiple endocrine neoplasia type 2 or (medullary) thyroid carcinoma before or at GLP-1RA therapy initiation | Family or personal history of multiple endocrine neoplasia type 2 or medullary thyroid carcinoma | Personal or first-degree relative(s) history of multiple endocrine neoplasia type 2 or medullary thyroid carcinoma. | Personal or family history of multiple endocrine neoplasia type 2 (MEN2) or familial medullary thyroid carcinoma. Personal history of non-familial medullary thyroid carcinoma. |
| **Recent cardiovascular events** | Diagnosis code for MI, stroke, unstable angina, or TIA during the 90-days before or at GLP-1RA initiation. | MI, stroke or hospitalization for unstable angina and/or TIA within the past 180-days prior to the day of screening | Any of the following: myocardial infarction, stroke, hospitalization for unstable angina or transient ischaemic attack within the past 60 days prior to screening. | An acute coronary or cerebrovascular event within the previous 14 days from Visit 2. |
| **Cardiovascular procedures** | Diagnosis code for coronary, carotid, or peripheral artery revascularization within 30-days before or at GLP-1RA initiation | Planned coronary, carotid, or peripheral artery revascularization on the day of screening | NA | Currently planned coronary, carotid or peripheral artery revascularization. |
| **NYHA** | Diagnosis code for NYHA class 4 during the 90-days before or at GLP-1RA initiation | Subjects presently classified as being in NYHA class 4 | Subject presently classified as being in New York Heart Association (NYHA) Class IV. | Chronic heart failure NYHA class IV. |
| **Renal impairment** | SUSTAIN 7 trial: Baseline eGFR <60 ml/min/1.73m^2^ STEP 2 trial: Baseline eGFR <30 ml/min/1.73m^2^, SUSTAIN 6 trial: Having diagnosis code for end-stage kidney disease prior to or at GLP-1RA initiation | eGFR <60 ml/min/1.73 m2 as per CKD-EPI | eGFR <30 mL/min/1.73 m2 (<60 mL/min/1.73 m2 in patients treated with SGLT2i) according to Chronic Kidney Disease Epidemiology Collaboration creatinine equation as defined by Kidney Disease: Improving Global Outcomes 2012 by the central laboratory at screening. | Chronic haemodialysis or chronic peritoneal dialysis. |
| **Abuse of drugs or alcohol** | Diagnosis code for drug or alcohol abuse recorded within the 180-days before or at GLP-1RA initiation. | NA | Known or suspected abuse of alcohol or recreational drugs | Known use of non-prescribed narcotics or illicit drugs. |
| **End-stage liver disease** | SUSTAIN 6 trial: Having diagnosis code for end-stage liver disease prior to or at GLP-1RA initiation | NA | NA | End stage liver disease, defined as the presence of acute or chronic liver disease and recent history of one or more of the following: ascites, encephalopathy, variceal bleeding, bilirubin ≥ 2.0 mg/dL, albumin level ≤3.5 g/dL, prothrombin time ≥4 seconds prolonged, international normalised ratio (INR) ≥1.7 or prior liver transplant. |
| **Organ transplant** | SUSTAIN 6 trial: Diagnosis code for solid organ transplant before or at GLP-1RA initiation. | NA | NA | A prior solid organ transplant or awaiting solid organ transplant. |
| **Surgery** | NA | NA | Surgery scheduled for the duration of the trial, except for minor surgical procedures, in the opinion of the investigator | NA |
| **Other medicines** | NA | NA | Use of non-herbal Chinese medicine or other non-herbal local medicine with unknown/unspecified content within 90 days before screening. | NA |
| *Mental health-related* | | | | |
| **Depression** | STEP 2 trial: Diagnosis code for depression within the 2 years before or at GLP-1RA initiation. | NA | History of major depressive disorder within 2 years before screening. A Patient Health Questionnaire-9 (PHQ-9) score of ≥ 15 at screening. | NA |
| **Severe psychiatric disorder** | STEP 2 trial: Diagnosis code for schizophrenia or bipolar disorder before or at GLP-1RA initiation. | NA | Diagnosis of other severe psychiatric disorder (e.g., schizophrenia, bipolar disorder). | NA |
| **Suicidal attempt** | STEP 2 trial: Diagnosis code for suicidal attempt ever before or at GLP-1RA initiation. | NA | A lifetime history of a suicidal attempt. | NA |
| **Suicidal behavior** | STEP 2 trial: Diagnosis code for suicidal behavior in the 30-days before or at GLP-1RA initiation | NA | Suicidal behaviour within 30 days before screening. Suicidal ideation corresponding to type 4 or 5 on the Columbia-Suicide Severity Rating Scale (C-SSRS) within the past 30 days before screening. | NA |
| Abbreviations: SUSTAIN Semaglutide Unabated Sustainability in Treatment of Type 2 Diabetes, STEP Semaglutide Treatment Effect in People With Obesity, UK United Kingdom, HbA1c glycated hemoglobin, IMRD IQVIA Medical Research Data, CVOT cardiovascular outcomes trial, NHS National Health Service, GLP-1RA glucagon-like peptide-1 receptor agonist, SGLT-2i sodium-glucose co-transporter-2 inhibitor, BMI body-mass index, NICE National Institute for Health and Care Excellence, NA not applied, CVD cardiovascular disease, MI, myocardial infarction, TIA transient ischemic attack, eGFR estimated glomerular filtration rate, NYHA New York Heart Association. | | | | |

| **Table S8: On-treatment safety events between treatment initiation and 210-days of follow-up, stratified by analysis set.** | | | | | | | | |
| --- | --- | --- | --- | --- | --- | --- | --- | --- |
| **Analysis set** | **Per-protocol analysis set** | | | | **Early-attrition analysis set** | | | |
| **Treatment strategy** | **Semaglutide**  **(n = 1901)** | | **Dulaglutide**  **(n = 2735)** | | **Semaglutide**  **(n = 1017)** | | **Dulaglutide**  **(n = 963)** | |
| **Safety events** | **n (%)** | **Rate of events per 100 patient-years** | **n (%)** | **Rate of events per 100 patient-years** | **n (%)** | **Rate of events per 100 patient-years** | **n (%)** | **Rate of events per 100 patient-years** |
| **Fatal events** | <7 | 0.0 | <7 | 0.0 | <7 | 0.0 | <7 | 1.3 |
| **Safety events that emerged on-treatment and occurred in ≥5% individuals in any group** | | | | | | | | |
| **Gastrointestinal safety events** | 144 (7.6) | 13.8 | 161 (5.9) | 10.6 | 56 (5.5) | 23.5 | 59 (6.1) | 26.1 |
| *Nausea* | 21 (1.1) | 1.9 | 18 (0.7) | 1.2 | 12 (1.2) | 4.9 | 14 (1.5) | 6.0 |
| *Vomiting* | 24 (1.3) | 2.2 | 15 (0.5) | 1.0 | 9 (0.9) | 3.7 | 9 (0.9) | 3.8 |
| *Diarrhea* | 37 (1.9) | 3.4 | 34 (1.2) | 2.2 | 14 (1.4) | 5.8 | 21 (2.2) | 9.0 |
| *Constipation* | 25 (1.3) | 2.3 | 30 (1.1) | 1.9 | 9 (0.9) | 3.7 | <7 | 1.7 |
| *Abdominal pain or distention* | 30 (1.6) | 2.8 | 47 (1.7) | 3.0 | 13 (1.3) | 5.3 | 14 (1.5) | 6.0 |
| *Dyspepsia or gastroesophageal reflux* | 28 (1.5) | 2.6 | 29 (1.1) | 1.9 | 8 (0.8) | 3.3 | 7 (0.7) | 3.0 |
| **Safety events that emerged on-treatment and occurred in ≥1% individuals in any group** | | | | | | | | |
| **Retinopathy** | 40 (2.1) | 3.7 | 69 (2.5) | 4.4 | 9 (0.9) | 3.7 | 10 (1) | 4.3 |
| **Headache** | 13 (0.7) | 1.2 | 26 (1) | 1.7 | 10 (1) | 4.1 | <7 | 2.1 |
| **Chronic kidney disease** | 22 (1.2) | 2.0 | 31 (1.1) | 2.0 | 7 (0.7) | 2.9 | 8 (0.8) | 3.4 |
| **Cardiovascular event** | 14 (0.7) | 1.3 | 30 (1.1) | 1.9 | <7 | 1.6 | <7 | 2.6 |
| **Depression** | 38 (2) | 3.5 | 34 (1.2) | 2.2 | 10 (1) | 4.1 | 10 (1) | 4.3 |
| **Safety events that emerged on-treatment and occurred in ≥0.5% individuals in any group** | | | | | | | | |
| **Heart failure** | 11 (0.6) | 1.0 | 21 (0.8) | 1.3 | <7 | 2.5 | <7 | 2.1 |
| **Gallbladder disorders** | <7 | 0.6 | <7 | 0.3 | <7 | 2.1 | <7 | 0.9 |
| *Cholelithiasis* | <7 | 0.4 | <7 | 0.3 | <7 | 1.2 | <7 | 0.4 |
| **Acute renal failure** | 13 (0.7) | 1.2 | 21 (0.8) | 1.3 | 7 (0.7) | 2.9 | <7 | 0.9 |
| **Cancer** | 14 (0.7) | 1.3 | 16 (0.6) | 1.0 | <7 | 2.1 | <7 | 1.3 |
| *Pancreatic carcinoma* | <7 | 0.0 | <7 | 0.0 | <7 | 0.0 | <7 | 0.0 |
| *Thyroid carcinoma* | <7 | 0.0 | <7 | 0.1 | <7 | 0.0 | <7 | 0.0 |
| **Other safety events emerging on-treatment** | | | | | | | | |
| **Hypoglycemia** | 7 (0.4) | 0.6 | <7 | 0.2 | <7 | 0.8 | <7 | 0.4 |
| **Suicidality** | <7 | 0.1 | <7 | 0.2 | <7 | 0.0 | <7 | 0.4 |
| **Pancreatitis** | <7 | 0.0 | <7 | 0.2 | <7 | 0.4 | <7 | 0.0 |
| **Diabetic ketoacidosis** | <7 | 0.0 | <7 | 0.0 | <7 | 0.0 | <7 | 0.4 |
| Data presented in n (%) and corresponding rate of events per 100 person-years. Counts <7 are suppressed to prevent person identification. | | | | | | | | |

| **Table S9: Subgroup analyses of 1-year primary endpoints in the per-protocol analysis set, based on REWIND trial subgroup definitions**^4^ **and SUSTAIN 7 trial dose comparisons.**^1^ | | | | | |
| --- | --- | --- | --- | --- | --- |
| **Subgroup^a^** | **Endpoint** | **Semaglutide** | **Dulaglutide** | **ETD**  **(95% CI)** | **p-value** |
| **Subgroup^a^ analyses informed by REWIND trial subgrouping**^4^ | | | | | |
| Age ≥65 years  Semaglutide, n = 556  Dulaglutide, n = 929 | Absolute change from baseline HbA1c, percentage points | -0.82 (0.06) | -0.65 (0.04) | -0.16  (-0.3, -0.03) | 0.0182 |
|  | Relative change from baseline HbA1c, % | -9.62 (0.69) | -7.6 (0.52) | -2.02  (-3.68, -0.36) | 0.0171 |
|  | Absolute change from baseline bodyweight, kg | -6.03 (0.76) | -3.95 (0.47) | -2.08  (-3.79, -0.37) | 0.0174 |
|  | Relative change from baseline bodyweight, % | -5.72 (0.87) | -3.59 (0.52) | -2.13  (-4.07, -0.2) | 0.0309 |
| Age <65 years  Semaglutide, n = 1345  Dulaglutide, n = 1806 | Absolute change from baseline HbA1c, percentage points | -0.89 (0.04) | -0.64 (0.03) | -0.25  (-0.34, -0.15) | <0.0001 |
|  | Relative change from baseline HbA1c, % | -10.65 (0.44) | -7.54 (0.4) | -3.11  (-4.24, -1.98) | <0.0001 |
|  | Absolute change from baseline bodyweight, kg | -5.26 (0.49) | -3.39 (0.44) | -1.88  (-3.14, -0.62) | 0.0036 |
|  | Relative change from baseline bodyweight, % | -4.36 (0.5) | -2.59 (0.44) | -1.77  (-3.05, -0.49) | 0.007 |
| Sex assigned at birth: Female  Semaglutide, n = 921  Dulaglutide, n = 1295 | Absolute change from baseline HbA1c, percentage points | -0.91 (0.04) | -0.68 (0.04) | -0.23  (-0.34, -0.12) | <0.0001 |
|  | Relative change from baseline HbA1c, % | -10.95 (0.55) | -7.97 (0.47) | -2.98  (-4.37, -1.59) | <0.0001 |
|  | Absolute change from baseline bodyweight, kg | -5.76 (0.72) | -3.94 (0.5) | -1.82  (-3.44, -0.2) | 0.028 |
|  | Relative change from baseline bodyweight, % | -5.14 (0.79) | -3.37 (0.51) | -1.77  (-3.55, 0.01) | 0.0507 |
| Sex assigned at birth: Male  Semaglutide, n = 980  Dulaglutide, n = 1440 | Absolute change from baseline HbA1c, percentage points | -0.84 (0.04) | -0.61 (0.04) | -0.22  (-0.32, -0.12) | <0.0001 |
|  | Relative change from baseline HbA1c, % | -9.88 (0.5) | -7.11 (0.44) | -2.77  (-4.04, -1.51) | <0.0001 |
|  | Absolute change from baseline bodyweight, kg | -5.26 (0.53) | -3.2 (0.43) | -2.06  (-3.35, -0.77) | 0.0019 |
|  | Relative change from baseline bodyweight, % | -4.4 (0.53) | -2.48 (0.44) | -1.92  (-3.21, -0.63) | 0.0037 |
| Cardiovascular disease history at baseline  Semaglutide, n = 319  Dulaglutide, n = 529 | Absolute change from baseline HbA1c, percentage points | -0.85 (0.09) | -0.57 (0.06) | -0.28  (-0.48, -0.07) | 0.0082 |
|  | Relative change from baseline HbA1c, % | -10.08 (1.06) | -6.57 (0.75) | -3.52  (-6.03, -1.01) | 0.0061 |
|  | Absolute change from baseline bodyweight, kg | -5.51 (1.01) | -3.88 (0.7) | -1.63  (-4.05, 0.79) | 0.185 |
|  | Relative change from baseline bodyweight, % | -5.23 (1.19) | -3.37 (0.8) | -1.87  (-4.73, 0.99) | 0.201 |
| No cardiovascular disease history at baseline  Semaglutide, n = 1582  Dulaglutide, n = 2206 | Absolute change from baseline HbA1c, percentage points | -0.88 (0.03) | -0.67 (0.03) | -0.2  (-0.29, -0.12) | <0.0001 |
|  | Relative change from baseline HbA1c, % | -10.48 (0.41) | -7.9 (0.36) | -2.58  (-3.61, -1.55) | <0.0001 |
|  | Absolute change from baseline bodyweight, kg | -5.54 (0.48) | -3.5 (0.4) | -2.04  (-3.18, -0.9) | 0.0005 |
|  | Relative change from baseline bodyweight, % | -4.76 (0.54) | -2.8 (0.4) | -1.96  (-3.21, -0.71) | 0.0022 |
| Baseline BMI ≥35 kg/m^2^  Semaglutide, n = 998  Dulaglutide, n = 1317 | Absolute change from baseline HbA1c, percentage points | -0.88 (0.04) | -0.68 (0.04) | -0.21  (-0.31, -0.1) | 0.0002 |
|  | Relative change from baseline HbA1c, % | -10.65 (0.52) | -8.04 (0.47) | -2.61  (-3.96, -1.26) | <0.0001 |
|  | Absolute change from baseline bodyweight, kg | -6.23 (0.6) | -4.28 (0.55) | -1.95  (-3.47, -0.43) | 0.0122 |
|  | Relative change from baseline bodyweight, % | -4.86 (0.57) | -3.15 (0.52) | -1.71  (-3.15, -0.27) | 0.0204 |
| Baseline BMI <35 kg/m^2^  Semaglutide, n = 903  Dulaglutide, n = 1418 | Absolute change from baseline HbA1c, percentage points | -0.86 (0.04) | -0.63 (0.03) | -0.23  (-0.34, -0.12) | <0.0001 |
|  | Relative change from baseline HbA1c, % | -10.06 (0.55) | -7.18 (0.42) | -2.88  (-4.2, -1.55) | <0.0001 |
|  | Absolute change from baseline bodyweight, kg | -4.82 (0.6) | -2.76 (0.39) | -2.07  (-3.39, -0.74) | 0.0023 |
|  | Relative change from baseline bodyweight, % | -4.76 (0.7) | -2.56 (0.45) | -2.2  (-3.75, -0.65) | 0.0056 |
| Baseline HbA1c ≥8%  Semaglutide, n = 1062  Dulaglutide, n = 1757 | Absolute change from baseline HbA1c, percentage points | -1.09 (0.05) | -0.84 (0.03) | -0.24  (-0.35, -0.13) | <0.0001 |
|  | Relative change from baseline HbA1c, % | -12.66 (0.58) | -9.76 (0.36) | -2.9  (-4.19, -1.6) | <0.0001 |
|  | Absolute change from baseline bodyweight, kg | -5.53 (0.62) | -3.35 (0.35) | -2.18  (-3.56, -0.8) | 0.0021 |
|  | Relative change from baseline bodyweight, % | -4.97 (0.67) | -2.83 (0.39) | -2.13  (-3.63, -0.63) | 0.0055 |
| Baseline HbA1c <8%  Semaglutide, n = 839  Dulaglutide, n = 978 | Absolute change from baseline HbA1c, percentage points | -0.54 (0.04) | -0.36 (0.04) | -0.18  (-0.29, -0.06) | 0.0028 |
|  | Relative change from baseline HbA1c, % | -6.85 (0.58) | -4.44 (0.55) | -2.41  (-3.96, -0.86) | 0.0023 |
|  | Absolute change from baseline bodyweight, kg | -5.64 (0.64) | -3.88 (0.68) | -1.76  (-3.4, -0.12) | 0.0353 |
|  | Relative change from baseline bodyweight, % | -4.68 (0.68) | -3.02 (0.67) | -1.66  (-3.35, 0.03) | 0.0536 |
| Number of co-prescribed glucose-lowering therapies =0 or 1  Semaglutide, n = 366  Dulaglutide, n = 459 | Absolute change from baseline HbA1c, percentage points | -0.88 (0.13) | -0.83 (0.06) | -0.05  (-0.34, 0.23) | 0.715 |
|  | Relative change from baseline HbA1c, % | -10.8 (1.67) | -10.12 (0.83) | -0.68  (-4.31, 2.94) | 0.712 |
|  | Absolute change from baseline bodyweight, kg | -6.21 (1.25) | -4.16 (1.14) | -2.05  (-5.1, 1) | 0.188 |
|  | Relative change from baseline bodyweight, % | -5.13 (1.28) | -3.09 (1.02) | -2.04  (-5.04, 0.96) | 0.182 |
| Number of co-prescribed glucose-lowering therapies =2  Semaglutide, n = 661  Dulaglutide, n = 951 | Absolute change from baseline HbA1c, percentage points | -0.9 (0.05) | -0.69 (0.04) | -0.21  (-0.35, -0.07) | 0.0031 |
|  | Relative change from baseline HbA1c, % | -10.8 (0.67) | -8.09 (0.56) | -2.71  (-4.46, -0.96) | 0.0025 |
|  | Absolute change from baseline bodyweight, kg | -5.55 (0.75) | -3.38 (0.59) | -2.17  (-4.05, -0.29) | 0.0237 |
|  | Relative change from baseline bodyweight, % | -4.73 (0.79) | -2.7 (0.61) | -2.03  (-4, -0.06) | 0.0434 |
| Number of co-prescribed glucose-lowering therapies ≥3  Semaglutide, n = 874  Dulaglutide, n = 1325 | Absolute change from baseline HbA1c, percentage points | -0.8 (0.05) | -0.55 (0.04) | -0.26  (-0.37, -0.14) | <0.0001 |
|  | Relative change from baseline HbA1c, % | -9.4 (0.58) | -6.24 (0.45) | -3.16  (-4.53, -1.8) | <0.0001 |
|  | Absolute change from baseline bodyweight, kg | -5.27 (0.62) | -3.49 (0.43) | -1.77  (-3.18, -0.37) | 0.0136 |
|  | Relative change from baseline bodyweight, % | -4.8 (0.67) | -2.99 (0.47) | -1.81  (-3.33, -0.29) | 0.0195 |
| Type 2 diabetes duration <5 years  Semaglutide, n = 999  Dulaglutide, n = 1405 | Absolute change from baseline HbA1c, percentage points | -0.87 (0.04) | -0.7 (0.04) | -0.17  (-0.27, -0.07) | 0.0013 |
|  | Relative change from baseline HbA1c, % | -10.43 (0.5) | -8.33 (0.45) | -2.1  (-3.38, -0.82) | 0.0013 |
|  | Absolute change from baseline bodyweight, kg | -5.34 (0.59) | -3.53 (0.51) | -1.81  (-3.29, -0.32) | 0.0175 |
|  | Relative change from baseline bodyweight, % | -4.5 (0.59) | -2.74 (0.52) | -1.75  (-3.25, -0.26) | 0.0218 |
| Type 2 diabetes duration 5-<10 years  Semaglutide, n = 425  Dulaglutide, n = 652 | Absolute change from baseline HbA1c, percentage points | -0.92 (0.07) | -0.62 (0.05) | -0.3  (-0.47, -0.13) | 0.0005 |
|  | Relative change from baseline HbA1c, % | -10.98 (0.88) | -7.18 (0.63) | -3.8  (-5.88, -1.73) | 0.0003 |
|  | Absolute change from baseline bodyweight, kg | -5.59 (0.95) | -3.3 (0.62) | -2.29  (-4.48, -0.09) | 0.0413 |
|  | Relative change from baseline bodyweight, % | -4.97 (0.96) | -2.69 (0.64) | -2.28  (-4.5, -0.06) | 0.0446 |
| Type 2 diabetes duration ≥10 years  Semaglutide, n = 477  Dulaglutide, n = 678 | Absolute change from baseline HbA1c, percentage points | -0.84 (0.07) | -0.55 (0.05) | -0.28  (-0.44, -0.12) | 0.0006 |
|  | Relative change from baseline HbA1c, % | -9.81 (0.78) | -6.32 (0.67) | -3.49  (-5.45, -1.53) | 0.0005 |
|  | Absolute change from baseline bodyweight, kg | -5.77 (0.86) | -3.89 (0.64) | -1.88  (-3.92, 0.16) | 0.0708 |
|  | Relative change from baseline bodyweight, % | -5.19 (1.04) | -3.45 (0.68) | -1.74  (-4.09, 0.62) | 0.147 |
| **Subgroup^b^ analyses informed by SUSTAIN 7 trial dose comparisons.**^1^ | | | | | |
| Dose comparisons corresponding to those assessed in the SUSTAIN 7 trial (weekly injectable semaglutide 0.5 mg versus dulaglutide 0.75 mg, and semaglutide 1.0 mg versus dulaglutide 1.5 mg)^1^ | | | | | |
| Semaglutide 0.5 mg, n = 856  Dulaglutide 0.75 mg, n = 574 | Absolute change from baseline HbA1c, percentage points | -0.85 (0.04) | -0.62 (0.06) | -0.23 (-0.37, -0.09) | 0.0009 |
|  | Relative change from baseline HbA1c, % | -10.17 (0.54) | -7.19 (0.71) | -2.98 (-4.69, -1.28) | 0.0006 |
|  | Absolute change from baseline bodyweight, kg | -4.95 (0.58) | -3.22 (0.72) | -1.73 (-3.47, 0.01) | 0.0517 |
|  | Relative change from baseline bodyweight, % | -4.41 (0.63) | -2.62 (0.73) | -1.79 (-3.59, 0.01) | 0.0516 |
| Semaglutide 1.0 mg, n = 897  Dulaglutide 1.5 mg, n = 1933 | Absolute change from baseline HbA1c, percentage points | -0.92 (0.04) | -0.66 (0.03) | -0.26 (-0.36, -0.16) | <0.0001 |
|  | Relative change from baseline HbA1c, % | -10.99 (0.54) | -7.73 (0.38) | -3.26 (-4.53, -2) | <0.0001 |
|  | Absolute change from baseline bodyweight, kg | -5.78 (0.62) | -3.78 (0.42) | -2.01 (-3.47, -0.54) | 0.0073 |
|  | Relative change from baseline bodyweight, % | -4.92 (0.67) | -3.1 (0.42) | -1.82 (-3.39, -0.25) | 0.023 |
| Dose comparisons not tested in SUSTAIN 7 (semaglutide 0.5 mg versus dulaglutide 1.5 mg, and semaglutide 1.0 mg versus dulaglutide 0.75 mg)^1^ | | | | | |
| Semaglutide 0.5 mg, n = 856  Dulaglutide 1.5 mg, n = 1933 | Absolute change from baseline HbA1c, percentage points | -0.87 (0.04) | -0.68 (0.03) | -0.19 (-0.29, -0.09) | 0.0002 |
|  | Relative change from baseline HbA1c, % | -10.32 (0.54) | -7.9 (0.36) | -2.42 (-3.67, -1.17) | 0.0002 |
|  | Absolute change from baseline bodyweight, kg | -5.11 (0.61) | -3.56 (0.4) | -1.55 (-2.86, -0.23) | 0.021 |
|  | Relative change from baseline bodyweight, % | -4.45 (0.7) | -2.94 (0.42) | -1.51 (-2.96, -0.05) | 0.0423 |
| Semaglutide 1.0 mg, n = 897  Dulaglutide 0.75 mg, n = 574 | Absolute change from baseline HbA1c, percentage points | -0.9 (0.04) | -0.58 (0.06) | -0.32 (-0.47, -0.18) | <0.0001 |
|  | Relative change from baseline HbA1c, % | -10.81 (0.55) | -6.71 (0.75) | -4.1 (-5.9, -2.3) | <0.0001 |
|  | Absolute change from baseline bodyweight, kg | -5.73 (0.66) | -3.61 (0.84) | -2.12 (-4.26, 0.01) | 0.0509 |
|  | Relative change from baseline bodyweight, % | -4.84 (0.71) | -2.86 (0.79) | -1.99 (-4.1, 0.12) | 0.065 |
| **Exploratory post-hoc subgroup analyses.** | | | | | |
| Stratifications based on concomitant medication use at baseline | | | | | |
| SGLT-2is co-prescribed  Semaglutide, n = 955  Dulaglutide, n = 1380 | Absolute change from baseline HbA1c, percentage points | -0.87 (0.04) | -0.65 (0.03) | -0.22 (-0.32, -0.12) | <0.0001 |
|  | Relative change from baseline HbA1c, % | -10.24 (0.51) | -7.52 (0.41) | -2.72 (-3.96, -1.48) | <0.0001 |
|  | Absolute change from baseline bodyweight, kg | -5.05 (0.64) | -3.16 (0.44) | -1.89 (-3.31, -0.46) | 0.0098 |
|  | Relative change from baseline bodyweight, % | -4.40 (0.69) | -2.59 (0.46) | -1.81 (-3.34, -0.28) | 0.0209 |
| No SGLT-2is co-prescribed  Semaglutide, n = 946  Dulaglutide, n = 1355 | Absolute change from baseline HbA1c, percentage points | -0.88 (0.04) | -0.65 (0.04) | -0.22 (-0.34, -0.11) | <0.0001 |
|  | Relative change from baseline HbA1c, % | -10.5 (0.55) | -7.67 (0.5) | -2.84 (-4.24, -1.43) | <0.0001 |
|  | Absolute change from baseline bodyweight, kg | -5.91 (0.61) | -4.01 (0.51) | -1.9 (-3.37, -0.43) | 0.0113 |
|  | Relative change from baseline bodyweight, % | -5.07 (0.66) | -3.26 (0.52) | -1.81 (-3.37, -0.25) | 0.0228 |
| Insulin co-prescribed  Semaglutide, n = 263  Dulaglutide, n = 340 | Absolute change from baseline HbA1c, percentage points | -0.58 (0.1) | -0.48 (0.09) | -0.1 (-0.36, 0.15) | 0.419 |
|  | Relative change from baseline HbA1c, % | -6.57 (1.32) | -5.22 (1.13) | -1.35 (-4.63, 1.92) | 0.417 |
|  | Absolute change from baseline bodyweight, kg | -4.85 (1.47) | -4.15 (1.11) | -0.7 (-4.21, 2.81) | 0.696 |
|  | Relative change from baseline bodyweight, % | -4.13 (1.63) | -3.49 (1.13) | -0.64 (-4.48, 3.19) | 0.743 |
| No insulin co-prescribed Semaglutide, n = 1638  Dulaglutide, n = 2395 | Absolute change from baseline HbA1c, percentage points | -0.91 (0.03) | -0.68 (0.03) | -0.23 (-0.31, -0.15) | <0.0001 |
|  | Relative change from baseline HbA1c, % | -10.86 (0.39) | -7.99 (0.33) | -2.87 (-3.85, -1.89) | <0.0001 |
|  | Absolute change from baseline bodyweight, kg | -5.62 (0.45) | -3.48 (0.37) | -2.13 (-3.21, -1.06) | 0.0001 |
|  | Relative change from baseline bodyweight, % | -4.89 (0.47) | -2.83 (0.38) | -2.06 (-3.18, -0.94) | 0.0003 |
| Dose comparisons^b^ for semaglutide (0.5 mg/1.0 mg) versus high-dose dulaglutide (3.0 mg/4.5 mg) | | | | | |
| Semaglutide 0.5 mg, n = 856  Dulaglutide 3.0 mg, n = 162 | Absolute change from baseline HbA1c, percentage points | -0.8 (0.2) | -0.63 (0.24) | -0.17 (-0.79, 0.46) | 0.6 |
|  | Relative change from baseline HbA1c, % | -9.48 (2.41) | -7.34 (2.95) | -2.14 (-9.9, 5.61) | 0.587 |
|  | Absolute change from baseline bodyweight, kg | -5.21 (2.7) | -3.96 (3.2) | -1.25 (-9.61, 7.12) | 0.77 |
|  | Relative change from baseline bodyweight, % | -4.53 (2.67) | -3.17 (3.21) | -1.36 (-9.72, 6.99) | 0.749 |
| Semaglutide 0.5 mg, n = 856  Dulaglutide 4.5 mg, n = 66 | Absolute change from baseline HbA1c, percentage points | -0.8 (0.23) | -1.36 (0.53) | 0.56 (-0.56, 1.69) | 0.323 |
|  | Relative change from baseline HbA1c, % | -9.49 (2.82) | -16.22 (6.12) | 6.73 (-6.36, 19.83) | 0.312 |
|  | Absolute change from baseline bodyweight, kg | -5.35 (2.86) | -3.37 (2.31) | -1.98 (-9.19, 5.23) | 0.589 |
|  | Relative change from baseline bodyweight, % | -4.73 (2.76) | -3.25 (2.52) | -1.48 (-8.91, 5.96) | 0.697 |
| Semaglutide 1.0 mg, n = 897  Dulaglutide 3.0 mg, n = 162 | Absolute change from baseline HbA1c, percentage points | -0.88 (0.1) | -0.52 (0.4) | -0.37 (-1.21, 0.47) | 0.391 |
|  | Relative change from baseline HbA1c, % | -10.54 (1.15) | -5.98 (5.23) | -4.57 (-15.49, 6.36) | 0.41 |
|  | Absolute change from baseline bodyweight, kg | -6.46 (1.99) | -4.06 (6.41) | -2.4 (-15.39, 10.59) | 0.715 |
|  | Relative change from baseline bodyweight, % | -5.49 (2.11) | -2.64 (10.25) | -2.85 (-23.25, 17.56) | 0.783 |
| Semaglutide 1.0 mg, n = 897  Dulaglutide 4.5 mg, n = 66 | Absolute change from baseline HbA1c, percentage points | -0.92 (0.49) | -0.83 (0.37) | -0.09 (-1.37, 1.19) | 0.888 |
|  | Relative change from baseline HbA1c, % | -10.7 (6.5) | -10 (4.99) | -0.73 (-17.78, 16.31) | 0.932 |
|  | Absolute change from baseline bodyweight, kg | -5.31 (5.86) | -4.06 (3.99) | -1.24 (-15.12, 12.64) | 0.86 |
|  | Relative change from baseline bodyweight, % | -4.41 (5.9) | -3.45 (4.73) | -0.96 (-15.75, 13.83) | 0.898 |
| Data are presented as mean (SE) change from baseline, with mean (95% CI) estimated treatment difference (ETD); using data from individuals in the per-protocol analysis set (i.e., those who remained on their assigned treatment strategy for more than 210 days after cohort entry), while on-treatment. ^a^Subgroups were defined using baseline characteristics; if a baseline measurement was imputed, the median value across imputed datasets was used for subgroup classification. ^b^Dose comparison subgroups were defined by segmenting follow-up into 30-day intervals, assigning each interval the highest dose recorded, and classifying each individual to the respective comparison based on the dose used for most of follow-up (i.e., the maintenance dose). Accordingly, among semaglutide initiators in the per-protocol analysis set (n=1901), were assigned to a maintenance of: 0.25 mg (n=148; 7.8%), 0.50 mg (n=856; 45.0%), and 1.00 mg (n=897; 47.2%). Among dulaglutide initiators in the per-protocol analysis set (n=2735), were assigned to a maintenance of: 0.75 mg (n=574; 21.0%), 1.50 mg (n=1933; 70.7%), 3.00 mg (n=162; 5.9%), and 4.50 mg (n=66; 2.4%). Abbreviations: ETD estimated treatment difference, HbA1c glycated hemoglobin, BMI body-mass index, SGLT-2i sodium-glucose co-transporter 2 inhibitor. | | | | | |

| **Table S10: Baseline demographic and clinical characteristics of semaglutide versus dulaglutide initiators in the full analysis set, before and after weighting.*** | | | | | | |
| --- | --- | --- | --- | --- | --- | --- |
| **Characteristics** | **Before weighting** | | | **After weighting** | | |
|  | **Semaglutide**  **(n = 2918)** | **Dulaglutide**  **(n = 3698)** | **SMD** | **Semaglutide**  **(n = 2917.5)** | **Dulaglutide**  **(n = 3698.5)** | **SMD** |
| **Demographic characteristics and lifestyle factors** | | | | | | |
| Age, years | 58.5 (11.8) | 60 (11.6) | 0.128 | 59.3 (11.7) | 59.3 (11.8) | 0.001 |
| Age 18-34 years | 109 (3.7%) | 93 (2.5%) | 0.07 | 87 (3%) | 110 (3%) | 0.001 |
| Age 35-49 years | 531 (18.2%) | 597 (16.1%) | 0.054 | 502 (17.2%) | 637 (17.2%) | 0.001 |
| Age 50-59 years | 907 (31.1%) | 1145 (31%) | 0.003 | 898 (30.8%) | 1140 (30.8%) | 0.001 |
| Age 60-69 years | 852 (29.2%) | 1080 (29.2%) | 0.001 | 858 (29.4%) | 1085 (29.3%) | 0.002 |
| Age ≥70 years | 519 (17.8%) | 783 (21.2%) | 0.086 | 572 (19.6%) | 727 (19.7%) | 0.001 |
| Sex assigned at birth: Female | 1416 (48.5%) | 1767 (47.8%) | 0.015 | 1393 (47.8%) | 1777 (48%) | 0.006 |
| Type 2 diabetes duration, years | 6.2 (5.9) | 6.6 (6.1) | 0.064 | 6.4 (6) | 6.4 (6) | 0.004 |
| Type 2 diabetes duration <5 years | 1535 (52.6%) | 1875 (50.7%) | 0.038 | 1503 (51.5%) | 1900 (51.4%) | 0.003 |
| Type 2 diabetes duration 5-<10 years | 676 (23.2%) | 881 (23.8%) | 0.016 | 691 (23.7%) | 873 (23.6%) | 0.002 |
| Type 2 diabetes duration ≥10 years | 707 (24.2%) | 942 (25.5%) | 0.029 | 723 (24.8%) | 925 (25%) | 0.005 |
| Smoking status: Current | 399 (13.7%) | 571 (15.4%) | 0.05 | 423 (14.5%) | 538 (14.6%) | 0.002 |
| Smoking status: Former | 1063 (36.4%) | 1360 (36.8%) | 0.007 | 1069 (36.6%) | 1353 (36.6%) | 0.001 |
| Smoking status: Never | 1452 (49.8%) | 1762 (47.6%) | 0.042 | 1422 (48.8%) | 1803 (48.7%) | 0.001 |
| Smoking status: Missing | <7 | <7 | 0.001 | <7 | <7 | 0.002 |
| Alcohol consumption: Current | 1859 (63.7%) | 2384 (64.5%) | 0.016 | 1872 (64.2%) | 2369 (64.1%) | 0.002 |
| Alcohol consumption: Former | 184 (6.3%) | 248 (6.7%) | 0.016 | 185 (6.3%) | 238 (6.4%) | 0.004 |
| Alcohol consumption: Never | 784 (26.9%) | 959 (25.9%) | 0.021 | 775 (26.6%) | 980 (26.5%) | 0.002 |
| Alcohol consumption: Missing | 91 (3.1%) | 107 (2.9%) | 0.013 | 86 (2.9%) | 111 (3%) | 0.004 |
| Cohort entry year: 2019 | 639 (21.9%) | 994 (26.9%) | 0.116 | 734 (25.2%) | 921 (24.9%) | 0.006 |
| Cohort entry year: 2020 | 728 (24.9%) | 735 (19.9%) | 0.122 | 643 (22%) | 817 (22.1%) | 0.001 |
| Cohort entry year: 2021 | 856 (29.3%) | 981 (26.5%) | 0.063 | 810 (27.8%) | 1028 (27.8%) | 0.001 |
| Cohort entry year: 2022 | 695 (23.8%) | 988 (26.7%) | 0.067 | 730 (25%) | 933 (25.2%) | 0.005 |
| **Co-prescribed glucose-lowering therapies** | | | | | | |
| Metformin | 2487 (85.2%) | 3112 (84.2%) | 0.03 | 2471 (84.7%) | 3132 (84.7%) | 0.001 |
| Sulfonylureas | 1028 (35.2%) | 1455 (39.3%) | 0.085 | 1098 (37.6%) | 1389 (37.6%) | 0.002 |
| Sodium-glucose co-transporter-2 inhibitors | 1382 (47.4%) | 1831 (49.5%) | 0.043 | 1426 (48.9%) | 1799 (48.6%) | 0.005 |
| Dipeptidyl peptidase-4 inhibitors | 1213 (41.6%) | 1628 (44%) | 0.05 | 1254 (43%) | 1586 (42.9%) | 0.002 |
| Thiazolidinediones | 117 (4%) | 159 (4.3%) | 0.015 | 122 (4.2%) | 155 (4.2%) | 0.001 |
| Other glucose-lowering therapies | <7 | 13 (0.4%) | 0.043 | 8 (0.3%) | 10 (0.3%) | 0.002 |
| Insulin | 460 (15.8%) | 535 (14.5%) | 0.036 | 441 (15.1%) | 561 (15.2%) | 0.002 |
| **Number of co-prescribed glucose-lowering therapies** | | | | | | |
| 0 | 62 (2.1%) | 54 (1.5%) | 0.05 | 51 (1.7%) | 64 (1.7%) | 0.001 |
| 1 | 553 (19%) | 617 (16.7%) | 0.059 | 508 (17.4%) | 651 (17.6%) | 0.005 |
| 2 | 1037 (35.5%) | 1304 (35.3%) | 0.006 | 1035 (35.5%) | 1310 (35.4%) | 0.002 |
| ≥3 | 1266 (43.4%) | 1723 (46.6%) | 0.064 | 1323 (45.4%) | 1674 (45.3%) | 0.002 |
| **Clinical measurements** | | | | | | |
| Bodyweight, kg | 101.8 (19.3) | 99.9 (18.9) | 0.1 | 100.8 (19.0) | 100.8 (19.1) | 0.002 |
| BMI, kg/m^2^ | 35.3 (5.6) | 34.8 (5.5) | 0.097 | 35.1 (5.5) | 35.1 (5.6) | 0.001 |
| BMI <25 kg/m^2^ | 58 (2%) | 89 (2.4%) | 0.03 | 61 (2.1%) | 81 (2.2%) | 0.006 |
| BMI 25-<30 kg/m^2^ | 442 (15.1%) | 647 (17.5%) | 0.064 | 480 (16.5%) | 608 (16.4%) | 0.001 |
| BMI 30-<35 kg/m^2^ | 963 (33%) | 1255 (33.9%) | 0.02 | 979 (33.6%) | 1239 (33.5%) | 0.002 |
| BMI 35-<40 kg/m^2^ | 847 (29%) | 1013 (27.4%) | 0.036 | 823 (28.2%) | 1041 (28.1%) | 0.001 |
| BMI ≥40 kg/m^2^ | 608 (20.8%) | 693 (18.7%) | 0.053 | 574 (19.7%) | 730 (19.7%) | 0.002 |
| HbA1c, % | 8 (0.7) | 8.1 (0.7) | 0.155 | 8 (0.7) | 8 (0.7) | 0.001 |
| HbA1c <8 % | 1342 (46%) | 1466 (39.7%) | 0.128 | 1242 (42.6%) | 1572 (42.5%) | 0.001 |
| HbA1c ≥8 % | 1576 (54%) | 2232 (60.3%) | 0.128 | 1676 (57.4%) | 2126 (57.5%) | 0.001 |
| Systolic blood pressure, mmHg | 133.2 (14.6) | 133.5 (15.4) | 0.023 | 133.4 (15) | 133.4 (15.1) | 0.002 |
| Systolic blood pressure <140 mmHg | 1995 (68.4%) | 2534 (68.5%) | 0.006 | 1991 (68.3%) | 2527 (68.3%) | 0.002 |
| Systolic blood pressure ≥140 mmHg | 923 (31.6%) | 1164 (31.5%) | 0.006 | 926 (31.7%) | 1171 (31.7%) | 0.002 |
| Diastolic blood pressure, mmHg | 78.7 (9.6) | 78.6 (9.7) | 0.013 | 78.6 (9.7) | 78.6 (9.7) | 0.002 |
| Diastolic blood pressure <90 mmHg | 2534 (86.8%) | 3244 (87.7%) | 0.026 | 2553 (87.5%) | 3235 (87.5%) | 0.001 |
| Diastolic blood pressure ≥90 mmHg | 384 (13.2%) | 454 (12.3%) | 0.026 | 365 (12.5%) | 464 (12.5%) | 0.001 |
| eGFR, ml/min/1.73m^2^ | 95.7 (22.2) | 93.2 (22) | 0.116 | 94.2 (22.2) | 94.3 (22.2) | 0.003 |
| eGFR <30 ml/min/1.73m^2^ | 13 (0.4%) | 16 (0.4%) | 0.002 | 12 (0.4%) | 16 (0.4%) | 0.003 |
| eGFR 30-<60 ml/min/1.73m^2^ | 258 (8.8%) | 363 (9.8%) | 0.034 | 277 (9.5%) | 349 (9.4%) | 0.002 |
| eGFR 60-<90 ml/min/1.73m^2^ | 620 (21.3%) | 850 (23%) | 0.042 | 650 (22.3%) | 822 (22.2%) | 0.002 |
| eGFR ≥90 ml/min/1.73m^2^ | 2027 (69.4%) | 2468 (66.7%) | 0.058 | 1978 (67.8%) | 2512 (67.9%) | 0.002 |
| **Comorbidities** | | | | | | |
| Cardiovascular disease | 524 (18%) | 732 (19.8%) | 0.047 | 555 (19%) | 702 (19%) | 0.001 |
| Cardiovascular event | 342 (11.7%) | 481 (13%) | 0.039 | 361 (12.4%) | 460 (12.4%) | 0.002 |
| Myocardial infarction | 201 (6.9%) | 272 (7.4%) | 0.018 | 208 (7.1%) | 265 (7.2%) | 0.002 |
| Unstable angina | 46 (1.6%) | 47 (1.3%) | 0.026 | 43 (1.5%) | 52 (1.4%) | 0.005 |
| Stroke | 155 (5.3%) | 248 (6.7%) | 0.059 | 175 (6%) | 224 (6.1%) | 0.003 |
| Coronary, carotid or peripheral arterial revascularization | 166 (5.7%) | 221 (6%) | 0.012 | 170 (5.8%) | 215 (5.8%) | 0.001 |
| Stenosis of coronary or carotid arteries, or lower extremity arteries | 82 (2.8%) | 134 (3.6%) | 0.046 | 97 (3.3%) | 121 (3.3%) | 0.003 |
| Heart failure | 135 (4.6%) | 195 (5.3%) | 0.03 | 145 (5%) | 185 (5%) | 0.002 |
| Hypertension | 1621 (55.6%) | 2105 (56.9%) | 0.028 | 1643 (56.3%) | 2083 (56.3%) | 0.001 |
| Dyslipidemia | 561 (19.2%) | 718 (19.4%) | 0.005 | 566 (19.4%) | 717 (19.4%) | 0.001 |
| Obesity treatment with surgery or a weight loss device | 30 (1%) | 20 (0.5%) | 0.055 | 22 (0.8%) | 28 (0.7%) | 0.001 |
| Asthma | 670 (23%) | 894 (24.2%) | 0.029 | 686 (23.5%) | 872 (23.6%) | 0.001 |
| Chronic obstructive pulmonary disease | 746 (25.6%) | 1006 (27.2%) | 0.037 | 770 (26.4%) | 979 (26.5%) | 0.001 |
| Sleep apnea | 150 (5.1%) | 161 (4.4%) | 0.037 | 136 (4.7%) | 173 (4.7%) | 0.001 |
| Osteoarthritis | 654 (22.4%) | 884 (23.9%) | 0.035 | 679 (23.3%) | 863 (23.3%) | 0.002 |
| Bone fracture | 39 (1.3%) | 58 (1.6%) | 0.019 | 42 (1.4%) | 54 (1.4%) | 0.001 |
| Non-alcoholic fatty liver disease | 294 (10.1%) | 310 (8.4%) | 0.059 | 265 (9.1%) | 337 (9.1%) | 0.001 |
| Pancreatitis | 38 (1.3%) | 57 (1.5%) | 0.02 | 44 (1.5%) | 55 (1.5%) | 0.002 |
| Gastrointestinal disorders | 220 (7.5%) | 295 (8%) | 0.016 | 225 (7.7%) | 287 (7.7%) | 0.002 |
| Gallbladder disorders | 305 (10.5%) | 354 (9.6%) | 0.029 | 291 (10%) | 369 (10%) | 0.001 |
| Hyperthyroidism | 18 (0.6%) | 36 (1%) | 0.04 | 26 (0.9%) | 31 (0.8%) | 0.006 |
| Hypothyroidism | 316 (10.8%) | 397 (10.7%) | 0.003 | 318 (10.9%) | 402 (10.9%) | 0.001 |
| Acute renal failure | 32 (1.1%) | 55 (1.5%) | 0.035 | 40 (1.4%) | 49 (1.3%) | 0.002 |
| Chronic kidney disease | 428 (14.7%) | 623 (16.8%) | 0.06 | 461 (15.8%) | 587 (15.9%) | 0.002 |
| Urinary tract infection | 154 (5.3%) | 183 (4.9%) | 0.015 | 152 (5.2%) | 192 (5.2%) | 0.001 |
| Retinopathy | 477 (16.3%) | 780 (21.1%) | 0.122 | 552 (18.9%) | 703 (19%) | 0.002 |
| Neuropathy | 379 (13%) | 561 (15.2%) | 0.063 | 418 (14.3%) | 531 (14.4%) | 0.001 |
| Hypoglycemia | 27 (0.9%) | 16 (0.4%) | 0.06 | 18 (0.6%) | 22 (0.6%) | 0.005 |
| Polycystic ovary syndrome | 106 (3.6%) | 100 (2.7%) | 0.053 | 90 (3.1%) | 115 (3.1%) | 0.001 |
| Gestational diabetes | 81 (2.8%) | 79 (2.1%) | 0.041 | 74 (2.5%) | 93 (2.5%) | 0.001 |
| Depression | 1254 (43%) | 1568 (42.4%) | 0.012 | 1236 (42.4%) | 1573 (42.5%) | 0.003 |
| Cancer | 225 (7.7%) | 332 (9%) | 0.046 | 246 (8.4%) | 312 (8.4%) | 0.001 |
| **Comedications** | | | | | | |
| Statins | 2154 (73.8%) | 2798 (75.7%) | 0.042 | 2189 (75%) | 2767 (74.8%) | 0.005 |
| Angiotensin converting enzyme inhibitors | 1266 (43.4%) | 1629 (44.1%) | 0.013 | 1275 (43.7%) | 1614 (43.7%) | 0.001 |
| Angiotensin II receptor blockers | 524 (18%) | 674 (18.2%) | 0.007 | 532 (18.2%) | 673 (18.2%) | 0.001 |
| Calcium channel blockers | 60 (2.1%) | 75 (2%) | 0.002 | 60 (2.1%) | 74 (2%) | 0.004 |
| Diuretics | 737 (25.3%) | 961 (26%) | 0.017 | 749 (25.7%) | 948 (25.6%) | 0.001 |
| Beta blockers | 760 (26%) | 993 (26.9%) | 0.018 | 769 (26.3%) | 973 (26.3%) | 0.001 |
| Antiplatelets | 206 (7.1%) | 302 (8.2%) | 0.042 | 228 (7.8%) | 286 (7.7%) | 0.003 |
| Acetylsalicylic acid | 523 (17.9%) | 699 (18.9%) | 0.025 | 535 (18.3%) | 684 (18.5%) | 0.004 |
| Anticoagulants | 230 (7.9%) | 310 (8.4%) | 0.018 | 237 (8.1%) | 299 (8.1%) | 0.002 |
| Antipsychotics | 156 (5.3%) | 235 (6.4%) | 0.043 | 172 (5.9%) | 218 (5.9%) | 0.001 |
| Selective serotonin reuptake inhibitors | 656 (22.5%) | 769 (20.8%) | 0.041 | 630 (21.6%) | 796 (21.5%) | 0.002 |
| Tricyclic antidepressants | 400 (13.7%) | 491 (13.3%) | 0.013 | 397 (13.6%) | 501 (13.5%) | 0.002 |
| Other antidepressants | 401 (13.7%) | 538 (14.5%) | 0.023 | 407 (13.9%) | 520 (14.1%) | 0.003 |
| Anxiolytics | 133 (4.6%) | 195 (5.3%) | 0.033 | 141 (4.8%) | 181 (4.9%) | 0.003 |
| Antiepileptics | 84 (2.9%) | 95 (2.6%) | 0.019 | 78 (2.7%) | 100 (2.7%) | 0.002 |
| Gabapentinoids | 321 (11%) | 421 (11.4%) | 0.012 | 323 (11.1%) | 413 (11.2%) | 0.003 |
| Opioids | 956 (32.8%) | 1273 (34.4%) | 0.035 | 980 (33.6%) | 1241 (33.6%) | 0.001 |
| Nonsteroidal anti-inflammatory drugs | 543 (18.6%) | 669 (18.1%) | 0.013 | 536 (18.4%) | 677 (18.3%) | 0.002 |
| Proton-pump inhibitors | 1339 (45.9%) | 1684 (45.5%) | 0.007 | 1334 (45.7%) | 1690 (45.7%) | 0.001 |
| Anti-obesity medications | 17 (0.6%) | 32 (0.9%) | 0.033 | 24 (0.8%) | 28 (0.8%) | 0.006 |
| Systemic corticosteroids | 211 (7.2%) | 297 (8%) | 0.03 | 225 (7.7%) | 283 (7.6%) | 0.002 |
| Thyroid hormone replacements | 335 (11.5%) | 392 (10.6%) | 0.028 | 326 (11.2%) | 411 (11.1%) | 0.002 |
| Data presented in n (%) or mean (SD). Weighted n (%) or mean (SD) represent the pseudo-population created by stabilized inverse probability of treatment weights (IPTWs) and may differ from the unweighted sample size. Individuals who are underrepresented in their treatment group relative to their covariate profile receive weights >1, while overrepresented individuals receive weights <1, resulting in totals that can exceed or fall below the original sample size and are expressed as continuous values (not integers). These weighted values do not represent actual numbers of individuals but rather the effective sample size in the weighted pseudo-population. Counts <7 are suppressed to prevent person identification. Abbreviations: Abbreviations: SMD standardized mean difference, BMI body-mass index, HbA1c glycated hemoglobin, eGFR estimated glomerular filtration rate. *Covariate balance was assessed within each imputed dataset using SMDs, which were then averaged across imputations (a pragmatic approach given the lack of consensus on how to evaluate balance after multiple imputation),^16^ while descriptive summaries were combined across imputations using Rubin’s rules.^7^ | | | | | | |

| **Table S11: Baseline demographic and clinical characteristics of semaglutide versus dulaglutide initiators in the per-protocol analysis set, before and after weighting.*** | | | | | | |
| --- | --- | --- | --- | --- | --- | --- |
| **Characteristics** | **Before weighting** | | | **After weighting** | | |
|  | **Semaglutide**  **(n = 1901)** | **Dulaglutide**  **(n = 2735)** | **SMD** | **Semaglutide**  **(n = 1902.7)** | **Dulaglutide**  **(n = 2734.6)** | **SMD** |
| **Demographic characteristics and lifestyle factors** | | | | | | |
| Age, years | 58.1 (11.6) | 59.9 (11.4) | 0.157 | 59.1 (11.5) | 59.1 (11.5) | 0.001 |
| Age 18-34 years | 73 (3.8%) | 57 (2.1%) | 0.104 | 52 (2.8%) | 75 (2.8%) | 0.001 |
| Age 35-49 years | 354 (18.6%) | 452 (16.5%) | 0.055 | 332 (17.5%) | 479 (17.5%) | 0.001 |
| Age 50-59 years | 603 (31.7%) | 862 (31.5%) | 0.004 | 597 (31.4%) | 857 (31.3%) | 0.001 |
| Age 60-69 years | 575 (30.2%) | 810 (29.6%) | 0.014 | 573 (30.1%) | 822 (30%) | 0.002 |
| Age ≥70 years | 296 (15.6%) | 554 (20.3%) | 0.122 | 348 (18.3%) | 502 (18.3%) | 0.002 |
| Sex assigned at birth: Female | 921 (48.4%) | 1295 (47.3%) | 0.022 | 896 (47.1%) | 1302 (47.6%) | 0.011 |
| Type 2 diabetes duration, years | 6.2 (5.9) | 6.5 (6.1) | 0.041 | 6.3 (5.9) | 6.4 (6) | 0.008 |
| Type 2 diabetes duration <5 years | 999 (52.6%) | 1405 (51.4%) | 0.024 | 985 (51.8%) | 1414 (51.7%) | 0.002 |
| Type 2 diabetes duration 5-<10 years | 425 (22.4%) | 652 (23.8%) | 0.035 | 453 (23.8%) | 640 (23.4%) | 0.009 |
| Type 2 diabetes duration ≥10 years | 477 (25.1%) | 678 (24.8%) | 0.007 | 465 (24.4%) | 681 (24.9%) | 0.01 |
| Smoking status: Current | 254 (13.4%) | 431 (15.8%) | 0.068 | 278 (14.6%) | 402 (14.7%) | 0.003 |
| Smoking status: Former | 716 (37.7%) | 1008 (36.9%) | 0.017 | 703 (37%) | 1016 (37.2%) | 0.004 |
| Smoking status: Never | 927 (48.8%) | 1291 (47.2%) | 0.031 | 918 (48.3%) | 1311 (48%) | 0.006 |
| Smoking status: Missing | <7 | <7 | 0.006 | <7 | <7 | 0.004 |
| Alcohol consumption: Current | 1232 (64.8%) | 1761 (64.4%) | 0.009 | 1235 (64.9%) | 1767 (64.6%) | 0.006 |
| Alcohol consumption: Former | 122 (6.4%) | 198 (7.2%) | 0.033 | 126 (6.6%) | 186 (6.8%) | 0.007 |
| Alcohol consumption: Never | 494 (26%) | 703 (25.7%) | 0.006 | 492 (25.9%) | 708 (25.9%) | 0.001 |
| Alcohol consumption: Missing | 53 (2.8%) | 73 (2.7%) | 0.007 | 50 (2.6%) | 74 (2.7%) | 0.006 |
| Cohort entry year: 2019 | 400 (21%) | 713 (26.1%) | 0.119 | 466 (24.5%) | 660 (24.1%) | 0.008 |
| Cohort entry year: 2020 | 484 (25.5%) | 533 (19.5%) | 0.143 | 415 (21.8%) | 599 (21.9%) | 0.002 |
| Cohort entry year: 2021 | 538 (28.3%) | 768 (28.1%) | 0.005 | 533 (28%) | 771 (28.2%) | 0.004 |
| Cohort entry year: 2022 | 479 (25.2%) | 721 (26.4%) | 0.027 | 489 (25.7%) | 705 (25.8%) | 0.002 |
| **Co-prescribed glucose-lowering therapies** | | | | | | |
| Metformin | 1655 (87.1%) | 2362 (86.4%) | 0.021 | 1654 (86.9%) | 2371 (86.7%) | 0.007 |
| Sulfonylureas | 678 (35.7%) | 1082 (39.6%) | 0.08 | 726 (38.2%) | 1040 (38%) | 0.003 |
| Sodium-glucose co-transporter-2 inhibitors | 955 (50.2%) | 1380 (50.5%) | 0.004 | 957 (50.3%) | 1375 (50.3%) | 0.001 |
| Dipeptidyl peptidase-4 inhibitors | 826 (43.5%) | 1263 (46.2%) | 0.055 | 862 (45.3%) | 1233 (45.1%) | 0.004 |
| Thiazolidinediones | 72 (3.8%) | 114 (4.2%) | 0.019 | 77 (4%) | 112 (4.1%) | 0.003 |
| Other glucose-lowering therapies | <7 | 8 (0.3%) | 0.058 | <7 | <7 | 0.006 |
| Insulin | 263 (13.8%) | 340 (12.4%) | 0.042 | 249 (13.1%) | 360 (13.2%) | 0.003 |
| **Number of co-prescribed glucose-lowering therapies** | | | | | | |
| 0 | 36 (1.9%) | 35 (1.3%) | 0.049 | 29 (1.5%) | 42 (1.5%) | 0.002 |
| 1 | 330 (17.4%) | 424 (15.5%) | 0.05 | 305 (16%) | 442 (16.2%) | 0.003 |
| 2 | 661 (34.8%) | 951 (34.8%) | 0.001 | 659 (34.6%) | 950 (34.7%) | 0.002 |
| ≥3 | 874 (46%) | 1325 (48.4%) | 0.049 | 910 (47.8%) | 1301 (47.6%) | 0.005 |
| **Clinical measurements** | | | | | | |
| Bodyweight, kg | 102.4 (19.1) | 100.6 (18.5) | 0.098 | 101.4 (18.7) | 101.4 (18.8) | 0.003 |
| BMI, kg/m^2^ | 35.5 (5.5) | 35 (5.5) | 0.093 | 35.2 (5.5) | 35.2 (5.5) | 0.002 |
| BMI <25 kg/m^2^ | 27 (1.4%) | 54 (2%) | 0.045 | 32 (1.7%) | 48 (1.8%) | 0.007 |
| BMI 25-<30 kg/m^2^ | 277 (14.6%) | 456 (16.7%) | 0.058 | 300 (15.8%) | 431 (15.8%) | 0.002 |
| BMI 30-<35 kg/m^2^ | 633 (33.3%) | 932 (34.1%) | 0.018 | 642 (33.8%) | 923 (33.7%) | 0.002 |
| BMI 35-<40 kg/m^2^ | 561 (29.5%) | 758 (27.7%) | 0.04 | 540 (28.4%) | 776 (28.4%) | 0.002 |
| BMI ≥40 kg/m^2^ | 403 (21.2%) | 535 (19.6%) | 0.041 | 388 (20.4%) | 557 (20.4%) | 0.002 |
| HbA1c, % | 8 (0.7) | 8.1 (0.7) | 0.148 | 8 (0.7) | 8 (0.7) | 0.002 |
| HbA1c <8 % | 871 (45.8%) | 1074 (39.3%) | 0.132 | 801 (42.1%) | 1149 (42%) | 0.003 |
| HbA1c ≥8 % | 1030 (54.2%) | 1661 (60.7%) | 0.132 | 1101 (57.9%) | 1586 (58%) | 0.003 |
| Systolic blood pressure, mmHg | 133 (14.4) | 133.5 (15.4) | 0.036 | 133.4 (15.1) | 133.4 (15.1) | 0.004 |
| Systolic blood pressure <140 mmHg | 1296 (68.2%) | 1878 (68.7%) | 0.011 | 1295 (68.1%) | 1866 (68.2%) | 0.003 |
| Systolic blood pressure ≥140 mmHg | 605 (31.8%) | 857 (31.3%) | 0.011 | 607 (31.9%) | 869 (31.8%) | 0.003 |
| Diastolic blood pressure, mmHg | 78.7 (9.6) | 78.6 (9.7) | 0.006 | 78.6 (9.6) | 78.7 (9.6) | 0.003 |
| Diastolic blood pressure <90 mmHg | 1649 (86.7%) | 2395 (87.6%) | 0.024 | 1662 (87.4%) | 2388 (87.3%) | 0.001 |
| Diastolic blood pressure ≥90 mmHg | 252 (13.3%) | 340 (12.4%) | 0.024 | 241 (12.6%) | 346 (12.7%) | 0.001 |
| eGFR, ml/min/1.73m^2^ | 96.4 (21.8) | 93.3 (21.9) | 0.143 | 94.4 (22) | 94.5 (21.9) | 0.003 |
| eGFR <30 ml/min/1.73m^2^ | 8 (0.4%) | 11 (0.4%) | 0.003 | 8 (0.4%) | 12 (0.4%) | 0.007 |
| eGFR 30-<60 ml/min/1.73m^2^ | 158 (8.3%) | 267 (9.8%) | 0.051 | 175 (9.2%) | 250 (9.1%) | 0.003 |
| eGFR 60-<90 ml/min/1.73m^2^ | 386 (20.3%) | 605 (22.1%) | 0.044 | 414 (21.7%) | 588 (21.5%) | 0.006 |
| eGFR ≥90 ml/min/1.73m^2^ | 1348 (70.9%) | 1851 (67.7%) | 0.07 | 1306 (68.6%) | 1885 (68.9%) | 0.006 |
| **Comorbidities** | | | | | | |
| Cardiovascular disease | 319 (16.8%) | 529 (19.3%) | 0.067 | 344 (18.1%) | 497 (18.2%) | 0.003 |
| Cardiovascular event | 211 (11.1%) | 344 (12.6%) | 0.046 | 225 (11.8%) | 326 (11.9%) | 0.003 |
| Myocardial infarction | 117 (6.2%) | 196 (7.2%) | 0.041 | 128 (6.7%) | 184 (6.7%) | 0.001 |
| Unstable angina | 20 (1.1%) | 31 (1.1%) | 0.008 | 24 (1.3%) | 30 (1.1%) | 0.015 |
| Stroke | 100 (5.3%) | 177 (6.5%) | 0.052 | 113 (5.9%) | 162 (5.9%) | 0.002 |
| Coronary, carotid or peripheral arterial revascularization | 93 (4.9%) | 156 (5.7%) | 0.036 | 102 (5.4%) | 147 (5.4%) | 0.001 |
| Stenosis of coronary or carotid arteries, or lower extremity arteries | 49 (2.6%) | 101 (3.7%) | 0.064 | 63 (3.3%) | 88 (3.2%) | 0.004 |
| Heart failure | 86 (4.5%) | 135 (4.9%) | 0.019 | 87 (4.6%) | 130 (4.7%) | 0.008 |
| Hypertension | 1079 (56.8%) | 1571 (57.4%) | 0.014 | 1084 (57%) | 1562 (57.1%) | 0.003 |
| Dyslipidemia | 364 (19.1%) | 534 (19.5%) | 0.01 | 377 (19.8%) | 534 (19.5%) | 0.008 |
| Obesity treatment with surgery or a weight loss device | 22 (1.2%) | 17 (0.6%) | 0.057 | 16 (0.8%) | 24 (0.9%) | 0.004 |
| Asthma | 442 (23.3%) | 650 (23.8%) | 0.012 | 444 (23.3%) | 644 (23.6%) | 0.005 |
| Chronic obstructive pulmonary disease | 487 (25.6%) | 720 (26.3%) | 0.016 | 490 (25.8%) | 712 (26%) | 0.006 |
| Sleep apnea | 107 (5.6%) | 122 (4.5%) | 0.053 | 93 (4.9%) | 135 (4.9%) | 0.002 |
| Osteoarthritis | 409 (21.5%) | 654 (23.9%) | 0.057 | 436 (22.9%) | 631 (23.1%) | 0.004 |
| Bone fracture | 22 (1.2%) | 41 (1.5%) | 0.03 | 24 (1.2%) | 37 (1.3%) | 0.009 |
| Non-alcoholic fatty liver disease | 206 (10.8%) | 223 (8.2%) | 0.092 | 175 (9.2%) | 252 (9.2%) | 0.001 |
| Pancreatitis | 25 (1.3%) | 40 (1.5%) | 0.013 | 26 (1.4%) | 39 (1.4%) | 0.006 |
| Gastrointestinal disorders | 125 (6.6%) | 220 (8%) | 0.056 | 139 (7.3%) | 204 (7.5%) | 0.005 |
| Gallbladder disorders | 197 (10.4%) | 255 (9.3%) | 0.035 | 186 (9.8%) | 268 (9.8%) | 0.001 |
| Hyperthyroidism | 9 (0.5%) | 27 (1%) | 0.06 | 16 (0.8%) | 22 (0.8%) | 0.006 |
| Hypothyroidism | 200 (10.5%) | 293 (10.7%) | 0.006 | 201 (10.6%) | 292 (10.7%) | 0.004 |
| Acute renal failure | 23 (1.2%) | 47 (1.7%) | 0.042 | 28 (1.5%) | 42 (1.5%) | 0.004 |
| Chronic kidney disease | 269 (14.2%) | 449 (16.4%) | 0.063 | 295 (15.5%) | 425 (15.6%) | 0.001 |
| Urinary tract infection | 100 (5.3%) | 127 (4.6%) | 0.028 | 94 (5%) | 136 (5%) | 0.001 |
| Retinopathy | 314 (16.5%) | 572 (20.9%) | 0.113 | 371 (19.5%) | 523 (19.1%) | 0.009 |
| Neuropathy | 243 (12.8%) | 397 (14.5%) | 0.05 | 269 (14.1%) | 385 (14.1%) | 0.002 |
| Hypoglycemia | 16 (0.8%) | 12 (0.4%) | 0.051 | 11 (0.6%) | 16 (0.6%) | 0.002 |
| Polycystic ovary syndrome | 73 (3.8%) | 67 (2.4%) | 0.08 | 58 (3%) | 84 (3.1%) | 0.002 |
| Gestational diabetes | 57 (3%) | 57 (2.1%) | 0.058 | 48 (2.5%) | 71 (2.6%) | 0.003 |
| Depression | 816 (42.9%) | 1157 (42.3%) | 0.013 | 801 (42.1%) | 1160 (42.4%) | 0.006 |
| Cancer | 124 (6.5%) | 251 (9.2%) | 0.099 | 157 (8.3%) | 223 (8.2%) | 0.004 |
| **Comedications** | | | | | | |
| Statins | 1397 (73.5%) | 2087 (76.3%) | 0.065 | 1434 (75.4%) | 2053 (75.1%) | 0.007 |
| Angiotensin converting enzyme inhibitors | 835 (43.9%) | 1234 (45.1%) | 0.024 | 843 (44.3%) | 1217 (44.5%) | 0.004 |
| Angiotensin II receptor blockers | 356 (18.7%) | 497 (18.2%) | 0.014 | 358 (18.8%) | 505 (18.5%) | 0.009 |
| Calcium channel blockers | 39 (2.1%) | 52 (1.9%) | 0.011 | 39 (2%) | 52 (1.9%) | 0.01 |
| Diuretics | 495 (26%) | 716 (26.2%) | 0.003 | 498 (26.1%) | 717 (26.2%) | 0.002 |
| Beta blockers | 500 (26.3%) | 738 (27%) | 0.015 | 503 (26.4%) | 725 (26.5%) | 0.002 |
| Antiplatelets | 128 (6.7%) | 220 (8%) | 0.05 | 148 (7.8%) | 206 (7.5%) | 0.009 |
| Acetylsalicylic acid | 322 (16.9%) | 514 (18.8%) | 0.048 | 335 (17.6%) | 493 (18%) | 0.011 |
| Anticoagulants | 143 (7.5%) | 222 (8.1%) | 0.022 | 147 (7.7%) | 211 (7.7%) | 0.001 |
| Antipsychotics | 101 (5.3%) | 182 (6.7%) | 0.057 | 112 (5.9%) | 165 (6%) | 0.007 |
| Selective serotonin reuptake inhibitors | 426 (22.4%) | 587 (21.5%) | 0.023 | 419 (22%) | 598 (21.9%) | 0.004 |
| Tricyclic antidepressants | 264 (13.9%) | 367 (13.4%) | 0.014 | 259 (13.6%) | 375 (13.7%) | 0.003 |
| Other antidepressants | 262 (13.8%) | 421 (15.4%) | 0.046 | 275 (14.4%) | 399 (14.6%) | 0.004 |
| Anxiolytics | 88 (4.6%) | 152 (5.6%) | 0.042 | 98 (5.2%) | 140 (5.1%) | 0.002 |
| Antiepileptics | 52 (2.7%) | 75 (2.7%) | 0.001 | 51 (2.7%) | 75 (2.7%) | 0.003 |
| Gabapentinoids | 208 (10.9%) | 336 (12.3%) | 0.042 | 222 (11.7%) | 323 (11.8%) | 0.004 |
| Opioids | 630 (33.1%) | 957 (35%) | 0.039 | 646 (34%) | 934 (34.2%) | 0.004 |
| Nonsteroidal anti-inflammatory drugs | 360 (18.9%) | 498 (18.2%) | 0.019 | 359 (18.9%) | 513 (18.8%) | 0.003 |
| Proton-pump inhibitors | 848 (44.6%) | 1239 (45.3%) | 0.014 | 858 (45.1%) | 1233 (45.1%) | 0.001 |
| Anti-obesity medications | 15 (0.8%) | 23 (0.8%) | 0.006 | 17 (0.9%) | 23 (0.8%) | 0.005 |
| Systemic corticosteroids | 143 (7.5%) | 204 (7.5%) | 0.002 | 141 (7.4%) | 203 (7.4%) | 0.001 |
| Thyroid hormone replacements | 213 (11.2%) | 294 (10.7%) | 0.015 | 208 (11%) | 301 (11%) | 0.002 |
| Data presented in n (%) or mean (SD). Weighted n (%) or mean (SD) represent the pseudo-population created by stabilized inverse probability of treatment weights (IPTWs) and may differ from the unweighted sample size. Individuals who are underrepresented in their treatment group relative to their covariate profile receive weights >1, while overrepresented individuals receive weights <1, resulting in totals that can exceed or fall below the original sample size and are expressed as continuous values (not integers). These weighted values do not represent actual numbers of individuals but rather the effective sample size in the weighted pseudo-population. Counts <7 are suppressed to prevent person identification. Abbreviations: Abbreviations: SMD standardized mean difference, BMI body-mass index, HbA1c glycated hemoglobin, eGFR estimated glomerular filtration rate. *Covariate balance was assessed within each imputed dataset using SMDs, which were then averaged across imputations (a pragmatic approach given the lack of consensus on how to evaluate balance after multiple imputation),^16^ while descriptive summaries were combined across imputations using Rubin’s rules.^7^ | | | | | | |

| **Table S12: Baseline demographic and clinical characteristics of semaglutide versus dulaglutide initiators in the early-attrition analysis set, before and after weighting.*** | | | | | | |
| --- | --- | --- | --- | --- | --- | --- |
| **Characteristics** | **Before weighting** | | | **After weighting** | | |
|  | **Semaglutide**  **(n = 1017)** | **Dulaglutide**  **(n = 963)** | **SMD** | **Semaglutide**  **(n = 1019.1)** | **Dulaglutide**  **(n = 961.2)** | **SMD** |
| **Demographic characteristics and lifestyle factors** | | | | | | |
| Age, years | 59.3 (12.3) | 60.4 (12.3) | 0.088 | 59.8 (12.1) | 59.8 (12.2) | 0.004 |
| Age 18-34 years | 36 (3.5%) | 36 (3.7%) | 0.011 | 36 (3.5%) | 34 (3.5%) | 0.002 |
| Age 35-49 years | 177 (17.4%) | 145 (15.1%) | 0.064 | 164 (16.1%) | 157 (16.3%) | 0.007 |
| Age 50-59 years | 304 (29.9%) | 283 (29.4%) | 0.011 | 307 (30.2%) | 285 (29.6%) | 0.012 |
| Age 60-69 years | 277 (27.2%) | 270 (28%) | 0.018 | 283 (27.7%) | 268 (27.9%) | 0.004 |
| Age ≥70 years | 223 (21.9%) | 229 (23.8%) | 0.044 | 229 (22.5%) | 218 (22.7%) | 0.004 |
| Sex assigned at birth: Female | 495 (48.7%) | 472 (49%) | 0.007 | 494 (48.5%) | 467 (48.6%) | 0.003 |
| Type 2 diabetes duration, years | 6.2 (5.9) | 6.5 (6.1) | 0.041 | 6.3 (5.9) | 6.4 (6) | 0.008 |
| Type 2 diabetes duration <5 years | 999 (52.6%) | 1405 (51.4%) | 0.024 | 985 (51.8%) | 1414 (51.7%) | 0.002 |
| Type 2 diabetes duration 5-<10 years | 425 (22.4%) | 652 (23.8%) | 0.035 | 453 (23.8%) | 640 (23.4%) | 0.009 |
| Type 2 diabetes duration ≥10 years | 477 (25.1%) | 678 (24.8%) | 0.007 | 465 (24.4%) | 681 (24.9%) | 0.01 |
| Smoking status: Current | 145 (14.3%) | 140 (14.5%) | 0.008 | 148 (14.5%) | 136 (14.1%) | 0.011 |
| Smoking status: Former | 347 (34.1%) | 352 (36.6%) | 0.051 | 356 (35%) | 338 (35.1%) | 0.004 |
| Smoking status: Never | 525 (51.6%) | 471 (48.9%) | 0.054 | 515 (50.6%) | 488 (50.7%) | 0.004 |
| Smoking status: Missing | <7 | <7 | 0.000 | <7 | <7 | 0.000 |
| Alcohol consumption: Current | 627 (61.7%) | 623 (64.7%) | 0.063 | 637 (62.5%) | 601 (62.6%) | 0.003 |
| Alcohol consumption: Former | 62 (6.1%) | 50 (5.2%) | 0.039 | 57 (5.6%) | 54 (5.6%) | 0.003 |
| Alcohol consumption: Never | 290 (28.5%) | 256 (26.6%) | 0.043 | 287 (28.2%) | 269 (28%) | 0.004 |
| Alcohol consumption: Missing | 38 (3.7%) | 34 (3.5%) | 0.011 | 38 (3.7%) | 36 (3.8%) | 0.003 |
| Cohort entry year: 2019 | 239 (23.5%) | 281 (29.2%) | 0.129 | 273 (26.7%) | 256 (26.6%) | 0.004 |
| Cohort entry year: 2020 | 244 (24%) | 202 (21%) | 0.072 | 228 (22.4%) | 215 (22.4%) | 0.002 |
| Cohort entry year: 2021 | 318 (31.3%) | 213 (22.1%) | 0.208 | 274 (26.9%) | 263 (27.4%) | 0.01 |
| Cohort entry year: 2022 | 216 (21.2%) | 267 (27.7%) | 0.151 | 244 (24%) | 228 (23.7%) | 0.007 |
| **Co-prescribed glucose-lowering therapies** | | | | | | |
| Metformin | 832 (81.8%) | 750 (77.9%) | 0.098 | 817 (80.1%) | 767 (79.8%) | 0.009 |
| Sulfonylureas | 350 (34.4%) | 373 (38.7%) | 0.09 | 375 (36.8%) | 354 (36.9%) | 0.003 |
| Sodium-glucose co-transporter-2 inhibitors | 427 (42%) | 451 (46.8%) | 0.098 | 464 (45.5%) | 432 (45%) | 0.011 |
| Dipeptidyl peptidase-4 inhibitors | 387 (38.1%) | 365 (37.9%) | 0.003 | 386 (37.9%) | 360 (37.5%) | 0.01 |
| Thiazolidinediones | 45 (4.4%) | 45 (4.7%) | 0.012 | 46 (4.5%) | 43 (4.5%) | 0.002 |
| Other glucose-lowering therapies | <7 | <7 | 0.035 | <7 | <7 | 0.006 |
| Insulin | 197 (19.4%) | 195 (20.2%) | 0.022 | 199 (19.5%) | 192 (19.9%) | 0.01 |
| **Number of co-prescribed glucose-lowering therapies** | | | | | | |
| 0 | 26 (2.6%) | 19 (2%) | 0.039 | 23 (2.3%) | 22 (2.3%) | 0.002 |
| 1 | 223 (21.9%) | 193 (20%) | 0.046 | 205 (20.1%) | 200 (20.8%) | 0.016 |
| 2 | 376 (37%) | 353 (36.7%) | 0.007 | 378 (37.1%) | 352 (36.6%) | 0.009 |
| ≥3 | 392 (38.5%) | 398 (41.3%) | 0.057 | 413 (40.5%) | 387 (40.3%) | 0.005 |
| **Clinical measurements** | | | | | | |
| Bodyweight, kg | 100.6 (19.5) | 97.9 (19.6) | 0.139 | 99.3 (19.6) | 99.3 (19.5) | 0.004 |
| BMI, kg/m^2^ | 35.1 (5.8) | 34.3 (5.7) | 0.134 | 34.7 (5.7) | 34.7 (5.7) | 0.004 |
| BMI <25 kg/m^2^ | 31 (3%) | 35 (3.6%) | 0.034 | 30 (3%) | 30 (3.1%) | 0.008 |
| BMI 25-<30 kg/m^2^ | 165 (16.2%) | 192 (19.9%) | 0.096 | 190 (18.7%) | 176 (18.3%) | 0.009 |
| BMI 30-<35 kg/m^2^ | 330 (32.5%) | 323 (33.6%) | 0.026 | 332 (32.5%) | 316 (32.9%) | 0.007 |
| BMI 35-<40 kg/m^2^ | 286 (28.1%) | 254 (26.4%) | 0.038 | 281 (27.6%) | 264 (27.5%) | 0.004 |
| BMI ≥40 kg/m^2^ | 205 (20.2%) | 158 (16.4%) | 0.096 | 186 (18.2%) | 175 (18.2%) | 0.005 |
| HbA1c, % | 7.9 (0.7) | 8.1 (0.7) | 0.16 | 8 (0.7) | 8 (0.7) | 0.004 |
| HbA1c <8 % | 471 (46.3%) | 392 (40.7%) | 0.113 | 446 (43.7%) | 423 (44%) | 0.006 |
| HbA1c ≥8 % | 546 (53.7%) | 571 (59.3%) | 0.113 | 573 (56.3%) | 538 (56%) | 0.006 |
| Systolic blood pressure, mmHg | 133.5 (15) | 133.5 (15.2) | 0.009 | 133.6 (15) | 133.5 (15) | 0.005 |
| Systolic blood pressure <140 mmHg | 699 (68.7%) | 656 (68.1%) | 0.015 | 692 (67.9%) | 657 (68.4%) | 0.011 |
| Systolic blood pressure ≥140 mmHg | 318 (31.3%) | 307 (31.9%) | 0.015 | 327 (32.1%) | 304 (31.6%) | 0.011 |
| Diastolic blood pressure, mmHg | 78.7 (9.7) | 78.4 (9.9) | 0.037 | 78.6 (9.9) | 78.5 (9.8) | 0.005 |
| Diastolic blood pressure <90 mmHg | 885 (87%) | 849 (88.2%) | 0.033 | 893 (87.7%) | 843 (87.7%) | 0.003 |
| Diastolic blood pressure ≥90 mmHg | 132 (13%) | 114 (11.8%) | 0.033 | 126 (12.3%) | 118 (12.3%) | 0.003 |
| eGFR, ml/min/1.73m^2^ | 94.5 (22.8) | 92.9 (22.3) | 0.071 | 93.7 (22.5) | 93.8 (22.7) | 0.004 |
| eGFR <30 ml/min/1.73m^2^ | <7 | <7 | 0.004 | <7 | <7 | 0.001 |
| eGFR 30-<60 ml/min/1.73m^2^ | 100 (9.8%) | 96 (10%) | 0.005 | 99 (9.8%) | 96 (10%) | 0.009 |
| eGFR 60-<90 ml/min/1.73m^2^ | 234 (23%) | 245 (25.4%) | 0.057 | 249 (24.4%) | 230 (23.9%) | 0.011 |
| eGFR ≥90 ml/min/1.73m^2^ | 678 (66.7%) | 617 (64.1%) | 0.055 | 666 (65.4%) | 630 (65.6%) | 0.005 |
| **Comorbidities** | | | | | | |
| Cardiovascular disease | 205 (20.2%) | 203 (21.1%) | 0.023 | 210 (20.6%) | 200 (20.8%) | 0.006 |
| Cardiovascular event | 131 (12.9%) | 137 (14.2%) | 0.039 | 136 (13.4%) | 131 (13.6%) | 0.006 |
| Myocardial infarction | 84 (8.3%) | 76 (7.9%) | 0.013 | 83 (8.2%) | 80 (8.3%) | 0.005 |
| Unstable angina | 26 (2.6%) | 16 (1.7%) | 0.062 | 21 (2.1%) | 20 (2.1%) | 0.003 |
| Stroke | 55 (5.4%) | 71 (7.4%) | 0.08 | 62 (6.1%) | 59 (6.2%) | 0.003 |
| Coronary, carotid or peripheral arterial revascularization | 73 (7.2%) | 65 (6.7%) | 0.017 | 74 (7.3%) | 70 (7.3%) | 0.003 |
| Stenosis of coronary or carotid arteries, or lower extremity arteries | 33 (3.2%) | 33 (3.4%) | 0.01 | 34 (3.3%) | 32 (3.3%) | 0.002 |
| Heart failure | 49 (4.8%) | 60 (6.2%) | 0.062 | 59 (5.8%) | 55 (5.7%) | 0.005 |
| Hypertension | 542 (53.3%) | 534 (55.5%) | 0.043 | 552 (54.2%) | 525 (54.6%) | 0.01 |
| Dyslipidemia | 197 (19.4%) | 184 (19.1%) | 0.007 | 195 (19.1%) | 184 (19.1%) | 0.003 |
| Obesity treatment with surgery or a weight loss device | 8 (0.8%) | <7 | 0.064 | <7 | <7 | 0.005 |
| Asthma | 228 (22.4%) | 244 (25.3%) | 0.068 | 242 (23.8%) | 226 (23.5%) | 0.006 |
| Chronic obstructive pulmonary disease | 259 (25.5%) | 286 (29.7%) | 0.095 | 283 (27.7%) | 266 (27.6%) | 0.003 |
| Sleep apnea | 43 (4.2%) | 39 (4%) | 0.009 | 43 (4.2%) | 39 (4%) | 0.009 |
| Osteoarthritis | 245 (24.1%) | 230 (23.9%) | 0.005 | 241 (23.7%) | 225 (23.4%) | 0.006 |
| Bone fracture | 17 (1.7%) | 17 (1.8%) | 0.007 | 19 (1.8%) | 18 (1.8%) | 0.002 |
| Non-alcoholic fatty liver disease | 88 (8.7%) | 87 (9%) | 0.013 | 90 (8.9%) | 86 (9%) | 0.004 |
| Pancreatitis | 13 (1.3%) | 17 (1.8%) | 0.04 | 16 (1.6%) | 15 (1.6%) | 0.004 |
| Gastrointestinal disorders | 95 (9.3%) | 75 (7.8%) | 0.056 | 88 (8.7%) | 81 (8.4%) | 0.008 |
| Gallbladder disorders | 108 (10.6%) | 99 (10.3%) | 0.011 | 106 (10.4%) | 99 (10.3%) | 0.004 |
| Hyperthyroidism | 9 (0.9%) | 9 (0.9%) | 0.005 | 10 (1%) | 9 (0.9%) | 0.006 |
| Hypothyroidism | 116 (11.4%) | 104 (10.8%) | 0.019 | 119 (11.7%) | 111 (11.5%) | 0.005 |
| Acute renal failure | 9 (0.9%) | 8 (0.8%) | 0.006 | 10 (1%) | 10 (1%) | 0.006 |
| Chronic kidney disease | 159 (15.6%) | 174 (18.1%) | 0.065 | 168 (16.5%) | 159 (16.6%) | 0.004 |
| Urinary tract infection | 54 (5.3%) | 56 (5.8%) | 0.022 | 59 (5.8%) | 58 (6%) | 0.008 |
| Retinopathy | 163 (16%) | 208 (21.6%) | 0.143 | 187 (18.4%) | 182 (18.9%) | 0.014 |
| Neuropathy | 136 (13.4%) | 164 (17%) | 0.102 | 152 (14.9%) | 147 (15.3%) | 0.011 |
| Hypoglycemia | 11 (1.1%) | 4 (0.4%) | 0.077 | 7 (0.7%) | <7 | 0.026 |
| Polycystic ovary syndrome | 33 (3.2%) | 33 (3.4%) | 0.01 | 33 (3.2%) | 30 (3.1%) | 0.005 |
| Gestational diabetes | 24 (2.4%) | 22 (2.3%) | 0.005 | 25 (2.5%) | 23 (2.4%) | 0.004 |
| Depression | 438 (43.1%) | 411 (42.7%) | 0.008 | 432 (42.4%) | 406 (42.3%) | 0.003 |
| Cancer | 101 (9.9%) | 81 (8.4%) | 0.053 | 93 (9.1%) | 87 (9.1%) | 0.002 |
| **Comedications** | | | | | | |
| Statins | 757 (74.4%) | 711 (73.8%) | 0.014 | 759 (74.5%) | 713 (74.2%) | 0.007 |
| Angiotensin converting enzyme inhibitors | 431 (42.4%) | 395 (41%) | 0.028 | 425 (41.8%) | 400 (41.6%) | 0.003 |
| Angiotensin II receptor blockers | 168 (16.5%) | 177 (18.4%) | 0.049 | 177 (17.4%) | 168 (17.5%) | 0.003 |
| Calcium channel blockers | 21 (2.1%) | 23 (2.4%) | 0.022 | 24 (2.4%) | 22 (2.3%) | 0.005 |
| Diuretics | 242 (23.8%) | 245 (25.4%) | 0.038 | 250 (24.5%) | 237 (24.6%) | 0.003 |
| Beta blockers | 260 (25.6%) | 255 (26.5%) | 0.021 | 259 (25.5%) | 246 (25.6%) | 0.004 |
| Antiplatelets | 78 (7.7%) | 82 (8.5%) | 0.031 | 82 (8.1%) | 78 (8.1%) | 0.003 |
| Acetylsalicylic acid | 201 (19.8%) | 185 (19.2%) | 0.014 | 198 (19.4%) | 189 (19.6%) | 0.005 |
| Anticoagulants | 87 (8.6%) | 88 (9.1%) | 0.021 | 91 (8.9%) | 85 (8.8%) | 0.004 |
| Antipsychotics | 55 (5.4%) | 53 (5.5%) | 0.004 | 57 (5.6%) | 54 (5.6%) | 0.003 |
| Selective serotonin reuptake inhibitors | 230 (22.6%) | 182 (18.9%) | 0.092 | 210 (20.6%) | 195 (20.3%) | 0.008 |
| Tricyclic antidepressants | 136 (13.4%) | 124 (12.9%) | 0.015 | 135 (13.2%) | 122 (12.7%) | 0.016 |
| Other antidepressants | 139 (13.7%) | 117 (12.1%) | 0.045 | 134 (13.1%) | 127 (13.2%) | 0.003 |
| Anxiolytics | 45 (4.4%) | 43 (4.5%) | 0.002 | 45 (4.4%) | 42 (4.4%) | 0.003 |
| Antiepileptics | 32 (3.1%) | 20 (2.1%) | 0.067 | 26 (2.5%) | 24 (2.5%) | 0.004 |
| Gabapentinoids | 113 (11.1%) | 85 (8.8%) | 0.076 | 98 (9.6%) | 91 (9.5%) | 0.006 |
| Opioids | 326 (32.1%) | 316 (32.8%) | 0.016 | 331 (32.5%) | 311 (32.4%) | 0.003 |
| Nonsteroidal anti-inflammatory drugs | 183 (18%) | 171 (17.8%) | 0.006 | 176 (17.3%) | 166 (17.2%) | 0.003 |
| Proton-pump inhibitors | 491 (48.3%) | 445 (46.2%) | 0.041 | 476 (46.7%) | 453 (47.1%) | 0.007 |
| Anti-obesity medications | <7 | <7 | 0.099 | 7 (0.7%) | <7 | 0.02 |
| Systemic corticosteroids | 68 (6.7%) | 93 (9.7%) | 0.109 | 88 (8.6%) | 81 (8.4%) | 0.006 |
| Thyroid hormone replacements | 122 (12%) | 98 (10.2%) | 0.058 | 117 (11.5%) | 109 (11.3%) | 0.005 |
| Data presented in n (%) or mean (SD). Weighted n (%) or mean (SD) represent the pseudo-population created by stabilized inverse probability of treatment weights (IPTWs) and may differ from the unweighted sample size. Individuals who are underrepresented in their treatment group relative to their covariate profile receive weights >1, while overrepresented individuals receive weights <1, resulting in totals that can exceed or fall below the original sample size and are expressed as continuous values (not integers). These weighted values do not represent actual numbers of individuals but rather the effective sample size in the weighted pseudo-population. Counts <7 are suppressed to prevent person identification. Abbreviations: Abbreviations: SMD standardized mean difference, BMI body-mass index, HbA1c glycated hemoglobin, eGFR estimated glomerular filtration rate. *Covariate balance was assessed within each imputed dataset using SMDs, which were then averaged across imputations (a pragmatic approach given the lack of consensus on how to evaluate balance after multiple imputation),^16^ while descriptive summaries were combined across imputations using Rubin’s rules.^7^ | | | | | | |

| **Table S13: Sensitivity analyses on primary outcomes (changes in glycated hemoglobin [HbA1c] and bodyweight from baseline to 1-year) conducted among individuals in the per-protocol analysis set.** | | | | |
| --- | --- | --- | --- | --- |
| **Endpoints** | **Semaglutide**  **(n = 1901)** | **Dulaglutide**  **(n = 2735)** | **Treatment comparison (95% CI)** | **p value** |
| **Primary analyses repeated using inverse probability of censoring weighting** | | | | |
| Absolute change from baseline HbA1c, percentage points | -0.88 (0.02) | -0.65 (0.02) | -0.23 (-0.27, -0.19) | <0.0001 |
| Percentage change from baseline HbA1c, % | -10.42 (0.21) | -7.57 (0.19) | -2.85 (-3.37, -2.33) | <0.0001 |
| Absolute change from baseline bodyweight, kg | -5.51 (0.3) | -3.58 (0.26) | -1.93 (-2.63, -1.23) | <0.0001 |
| Percentage change from baseline bodyweight, % | -4.78 (0.32) | -2.92 (0.27) | -1.87 (-2.6, -1.13) | <0.0001 |
| **Primary analyses repeated using stabilized inverse probability of treatment weights trimmed at the 1st and 99th percentiles.** | | | | |
| Absolute change from baseline HbA1c, percentage points | -0.87 (0.03) | -0.66 (0.03) | -0.21 (-0.29, -0.14) | <0.0001 |
| Percentage change from baseline HbA1c, % | -10.36 (0.37) | -7.66 (0.32) | -2.69 (-3.62, -1.77) | <0.0001 |
| Absolute change from baseline bodyweight, kg | -5.48 (0.42) | -3.56 (0.34) | -1.92 (-2.91, -0.93) | 0.0002 |
| Percentage change from baseline bodyweight, % | -4.75 (0.44) | -2.91 (0.36) | -1.84 (-2.87, -0.81) | 0.0005 |
| **Primary analyses repeated without using stabilized inverse probability of treatment weights.** | | | | |
| Absolute change from baseline HbA1c, percentage points | -0.83 (0.03) | -0.67 (0.02) | -0.16 (-0.23, -0.08) | <0.0001 |
| Percentage change from baseline HbA1c, % | -9.94 (0.36) | -7.86 (0.31) | -2.07 (-3, -1.15) | <0.0001 |
| Absolute change from baseline bodyweight, kg | -5.58 (0.41) | -3.52 (0.33) | -2.06 (-3.06, -1.07) | <0.0001 |
| Percentage change from baseline bodyweight, % | -4.8 (0.42) | -2.9 (0.36) | -1.9 (-2.94, -0.85) | 0.0004 |
| **Primary analyses repeated using the respective complete case analysis set.** | | | | |
| Absolute change from baseline HbA1c, percentage points | -0.96 (0.03)  N=867 | -0.77 (0.03)  N=1137 | -0.19 (-0.28, -0.11) | <0.0001 |
| Percentage change from baseline HbA1c, % | -11.66 (0.41)  N=867 | -9.17 (0.37)  N=1137 | -2.49 (-3.58, -1.41) | <0.0001 |
| Absolute change from baseline bodyweight, kg | -6.14 (0.25)  N=836 | -3.63 (0.17)  N=1245 | -2.52 (-3.11, -1.92) | <0.0001 |
| Percentage change from baseline bodyweight, % | -6.05 (0.26)  N=836 | -3.61 (0.17)  N=1245 | -2.45 (-3.05, -1.84) | <0.0001 |
| **Primary analyses repeated using propensity score matching*.** | | | | |
| Absolute change from baseline HbA1c, percentage points | -0.94 (0.03)  N=827 | -0.79 (0.03)  N=827 | -0.15 (-0.24, -0.05) | 0.0017 |
| Absolute change from baseline bodyweight, kg | -5.96 (0.26)  N=809 | -3.52 (0.20)  N=809 | -2.44 (-3.08, -1.80) | <0.0001 |
| **Primary analyses repeated by changing the endpoint assessment window to 365-days post-index ±90-days.** | | | | |
| Absolute change from baseline HbA1c, percentage points | -0.97 (0.04)  N=525 | -0.78 (0.04)  N=664 | -0.19 (-0.31, -0.07) | 0.00125 |
| Absolute change from baseline bodyweight, kg | -5.90 (0.32)  N=495 | -3.48 (0.21)  N=762 | -2.42 (-3.17, -1.67) | <0.0001 |
| **Primary analyses repeated by changing the endpoint assessment window to 365-days post-index ±60-days.** | | | | |
| Absolute change from baseline HbA1c, percentage points | -0.97 (0.06)  N=347 | -0.75 (0.04)  N=463 | -0.21 (-0.36, -0.07) | 0.00345 |
| Absolute change from baseline bodyweight, kg | -6.03 (0.41)  N=335 | -3.13 (0.25)  N=535 | -2.90 (-3.84, -1.96) | <0.0001 |
| **Primary analyses repeated by changing the endpoint assessment window to 365-days post-index ±30-days.** | | | | |
| Absolute change from baseline HbA1c, percentage points | -0.99 (0.09)  N=176 | -0.71 (0.07)  N=230 | -0.28 (-0.50, -0.05) | 0.015 |
| Absolute change from baseline bodyweight, kg | -5.88 (0.72)  N=182 | -2.58 (0.32)  N=301 | -3.30 (-4.84, -1.75) | <0.0001 |
| **Primary analyses repeated according to intention-to-treat principle using the respective complete case analysis set.** | | | | |
| Percentage change from baseline bodyweight, % | -6.17 (0.25)  N=901 | -3.58 (0.17)  N=1314 | -2.59 (-3.17, -2) | <0.0001 |
| Percentage change from baseline HbA1c, % | -11.48 (0.4)  N=926 | -9.02 (0.36)  N=1200 | -2.46 (-3.51, -1.41) | <0.0001 |
| **Primary analyses repeated by varying the primary definition of the permissible gap between consecutive prescriptions from 90-days to 60-days.** | | | | |
| Percentage change from baseline bodyweight, % | -6.29 (0.3)  N=630 | -3.68 (0.19)  N=1047 | -2.61 (-3.3, -1.92) | <0.0001 |
| Percentage change from baseline HbA1c, % | -12.36 (0.47)  N=667 | -9.56 (0.4)  N=943 | -2.79 (-4.01, -1.58) | <0.0001 |
| **Primary analyses repeated by varying the primary definition of the permissible gap between consecutive prescriptions from 90-days to 120-days.** | | | | |
| Percentage change from baseline bodyweight, % | -6.09 (0.25)  N=856 | -3.6 (0.17)  N=1262 | -2.49 (-3.08, -1.89) | <0.0001 |
| Percentage change from baseline HbA1c, % | -11.7 (0.41)  N=882 | -9.16 (0.37)  N=1152 | -2.53 (-3.61, -1.46) | <0.0001 |
| **Primary analyses repeated by varying the primary definition of the permissible gap between consecutive prescriptions from 90-days to 180-days.** | | | | |
| Percentage change from baseline bodyweight, % | -6.07 (0.25)  N=864 | -3.57 (0.17)  N=1268 | -2.5 (-3.09, -1.91) | <0.0001 |
| Percentage change from baseline HbA1c, % | -11.65 (0.41)  N=887 | -9.12 (0.36)  N=1155 | -2.53 (-3.6, -1.45) | <0.0001 |
| Data are presented as mean (SE) change from baseline, with mean (95% CI) estimated treatment difference (ETD); with details on sensitivity analyses specified in Supplementary Table S1. *Nearest-neighbor matching (1:1 ratio), with a caliper width of 0.2 standard deviations (on the logit of the propensity score), without replacement; propensity scores estimated using the same logistic regression model as in the primary inverse probability of treatment weighting analysis. | | | | |

| **Table S14: Individuals considered eligible for the SUSTAIN 7,**^1^ **STEP 2,**^2^ **and SUSTAIN 6**^3^ **trials based on eligibility criteria specified in Supplementary Table S7, stratified by analysis set and treatment strategy.** | | | | | | | |
| --- | --- | --- | --- | --- | --- | --- | --- |
|  | **Full analysis set** | | | **Per-protocol analysis set** | | **Early-attrition analysis set** | |
| **Eligibility criteria** | **Overall** | **Semaglutide** | **Dulaglutide** | **Semaglutide** | **Dulaglutide** | **Semaglutide** | **Dulaglutide** |
| n | 6616 | 2918 | 3698 | 1901 | 2735 | 1017 | 963 |
| **Eligibility criteria considered consistent across selected trials** | | | | | | | |
| Female or Male | 6616 (100.0) | 2918 (100.0) | 3698 (100.0) | 1901 (100.0) | 2735 (100.0) | 1017 (100.0) | 963 (100.0) |
| Age ≥18 years | 6616 (100.0) | 2918 (100.0) | 3698 (100.0) | 1901 (100.0) | 2735 (100.0) | 1017 (100.0) | 963 (100.0) |
| Type 2 diabetes diagnosis before or at GLP-1RA therapy initiation | 6616 (100.0) | 2918 (100.0) | 3698 (100.0) | 1901 (100.0) | 2735 (100.0) | 1017 (100.0) | 963 (100.0) |
| HbA1c ≥7% at GLP-1RA therapy initiation | 6111 (92.4) | 2648 (90.7) | 3463 (93.6) | 1726 (90.8) | 2566 (93.8) | 922 (90.7) | 897 (93.1) |
| No type 1 diabetes diagnosis before or at GLP-1RA therapy initiation | 6616 (100.0) | 2918 (100.0) | 3698 (100.0) | 1901 (100.0) | 2735 (100.0) | 1017 (100.0) | 963 (100.0) |
| No diagnosis code for retinopathy or maculopathy during the 90-days before or at GLP-1RA initiation | 6527 (98.7) | 2887 (98.9) | 3640 (98.4) | 1879 (98.8) | 2697 (98.6) | 1008 (99.1) | 943 (97.9) |
| No diagnosis code for pregnancy or breastfeeding during the 1-year before or at GLP-1RA initiation | 6612 (99.9) | 2915 (99.9) | 3697 (100.0) | 1898 (99.8) | 2734 (100.0) | 1017 (100.0) | 963 (100.0) |
| No diagnosis code for acute pancreatitis before or at GLP-1RA initiation | 6542 (98.9) | 2891 (99.1) | 3651 (98.7) | 1882 (99.0) | 2701 (98.8) | 1009 (99.2) | 950 (98.7) |
| No diagnosis code for chronic pancreatitis before or at GLP-1RA initiation | 6597 (99.7) | 2906 (99.6) | 3691 (99.8) | 1894 (99.6) | 2730 (99.8) | 1012 (99.5) | 961 (99.8) |
| No diagnosis code for cancer (except for non-melanoma skin cancers) in the 5-years before or at GLP-1RA initiation | 6375 (96.4) | 2821 (96.7) | 3554 (96.1) | 1850 (97.3) | 2629 (96.1) | 971 (95.5) | 925 (96.1) |
| No diagnosis code for thyroid carcinoma (incl. multiple endocrine neoplasia type 2 or medullary thyroid carcinoma) before or at GLP-1RA initiation | 6608 (99.9) | 2915 (99.9) | 3693 (99.9) | 1900 (99.9) | 2730 (99.8) | 1015 (99.8) | 963 (100.0) |
| No diagnosis code myocardial infarction, stroke, unstable angina, or transient ischemic attack in the 90-days before or at GLP-1RA initiation | 6569 (99.3) | 2900 (99.4) | 3669 (99.2) | 1891 (99.5) | 2719 (99.4) | 1009 (99.2) | 950 (98.7) |
| No diagnosis code for coronary, carotid, or peripheral artery revascularization in the 30-days before or at GLP-1RA initiation | 6606 (99.8) | 2913 (99.8) | 3693 (99.9) | 1898 (99.8) | 2732 (99.9) | 1015 (99.8) | 961 (99.8) |
| No diagnosis code for NYHA Class IV in the 90-days before or at GLP-1RA initiation | 6616 (100.0) | 2918 (100.0) | 3698 (100.0) | 1901 (100.0) | 2735 (100.0) | 1017 (100.0) | 963 (100.0) |
| No diagnosis code for abuse of alcohol or recreational drugs in the 180-days before or at GLP-1RA initiation | 6612 (99.9) | 2916 (99.9) | 3696 (99.9) | 1899 (99.9) | 2734 (100.0) | 1017 (100.0) | 962 (99.9) |
| **Individuals included based on all eligibility criteria deemed consistent across the selected trials.** | **5673 (85.7)** | **2472 (84.7)** | **3201 (86.6)** | **1621 (85.3)** | **2378 (86.9)** | **851 (83.7)** | **823 (85.5)** |
| **Eligibility criteria specific to the SUSTAIN 7 trial** | | | | | | | |
| Baseline HbA1c 7-10.5% | 6111 (92.4) | 2648 (90.7) | 3463 (93.6) | 1726 (90.8) | 2566 (93.8) | 922 (90.7) | 897 (93.1) |
| ≥1 prescription for metformin in the 90-days before or at GLP-1RA initiation | 5424 (82.0) | 2403 (82.4) | 3021 (81.7) | 1607 (84.5) | 2305 (84.3) | 796 (78.3) | 716 (74.4) |
| No prescription for any medication for the indication of diabetes or obesity other than stated in the inclusion criteria in the 90-days before or at GLP-1RA initiation (excl. short-term insulin treatment) | 1160 (17.5) | 575 (19.7) | 585 (15.8) | 355 (18.7) | 427 (15.6) | 220 (21.6) | 158 (16.4) |
| Baseline eGFR ≥60 ml/min/1.73 m^2^ | 5968 (90.2) | 2648 (90.7) | 3320 (89.8) | 1735 (91.3) | 2457 (89.8) | 913 (89.8) | 863 (89.6) |
| **SUSTAIN 7 trial-eligible individuals** | **847 (12.8)** | **413 (14.2)** | **434 (11.7)** | **256 (13.5)** | **316 (11.6)** | **157 (15.4)** | **118 (12.3)** |
| **Eligibility criteria specific to the STEP trial** | | | | | | | |
| Baseline HbA1c 7-10% | 6111 (92.4) | 2648 (90.7) | 3463 (93.6) | 1726 (90.8) | 2566 (93.8) | 922 (90.7) | 897 (93.1) |
| Baseline BMI ≥27 kg/m^2^ | 6321 (95.5) | 2796 (95.8) | 3525 (95.3) | 1835 (96.5) | 2623 (95.9) | 961 (94.5) | 902 (93.7) |
| Prescribed <4 oral glucose-lowering drugs (metformin, sulfonylureas, SGLT-2is, or thiazolidinediones) in the 90-days before or at GLP-1RA initiation | 3896 (58.9) | 1768 (60.6) | 2128 (57.5) | 1115 (58.7) | 1517 (55.5) | 653 (64.2) | 611 (63.4) |
| No prescription for any medication for the indication of diabetes or obesity other than stated in the inclusion criteria in the 90-days before or at GLP-1RA initiation | 3113 (47.1) | 1409 (48.3) | 1704 (46.1) | 908 (47.8) | 1259 (46.0) | 501 (49.3) | 445 (46.2) |
| No diagnosis code for liposuction and/or abdominoplasty in the 1-year before or at GLP-1RA initiation | 6616 (100.0) | 2918 (100.0) | 3698 (100.0) | 1901 (100.0) | 2735 (100.0) | 1017 (100.0) | 963 (100.0) |
| No diagnosis code for obesity treatment with surgery or a weight loss device before or at GLP-1RA initiation | 6566 (99.2) | 2888 (99.0) | 3678 (99.5) | 1879 (98.8) | 2718 (99.4) | 1009 (99.2) | 960 (99.7) |
| Baseline eGFR ≥30 ml/min/1.73 m^2^ | 6587 (99.6) | 2905 (99.6) | 3682 (99.6) | 1893 (99.6) | 2724 (99.6) | 1012 (99.5) | 958 (99.5) |
| No diagnosis code for depression in the 2-years before or at GLP-1RA initiation | 6272 (94.8) | 2767 (94.8) | 3505 (94.8) | 1787 (94.0) | 2599 (95.0) | 980 (96.4) | 906 (94.1) |
| No diagnosis code for suicidal attempt before or at GLP-1RA initiation | 6537 (98.8) | 2882 (98.8) | 3655 (98.8) | 1878 (98.8) | 2701 (98.8) | 1004 (98.7) | 954 (99.1) |
| No diagnosis code for suicidal behavior in the 30-days before or at GLP-1RA initiation | 6615 (100.0) | 2918 (100.0) | 3697 (100.0) | 1901 (100.0) | 2734 (100.0) | 1017 (100.0) | 963 (100.0) |
| No diagnosis code for schizophrenia or bipolar disorder before or at GLP-1RA initiation | 6485 (98.0) | 2866 (98.2) | 3619 (97.9) | 1865 (98.1) | 2676 (97.8) | 1001 (98.4) | 943 (97.9) |
| No uncontrolled thyroid disease, defined as thyroid-stimulating hormone >6 mlU/L or <0.4 mlU/L measured within the 90-days prior to GLP-1RA initiation | 6448 (97.5) | 2850 (97.7) | 3598 (97.3) | 1857 (97.7) | 2666 (97.5) | 993 (97.6) | 932 (96.8) |
| **STEP 2 trial-eligible individuals** | **2233 (33.8)** | **998 (34.2)** | **1235 (33.4)** | **640 (33.7)** | **927 (33.9)** | **358 (35.2)** | **308 (32.0)** |
| **Eligibility criteria specific to the SUSTAIN 6 trial** | | | | | | | |
| <4 glucose-lowering drugs (incl. insulin) in the 90-days before or at GLP-1RA initiation | 6154 (93.0) | 2719 (93.2) | 3435 (92.9) | 1768 (93.0) | 2539 (92.8) | 951 (93.5) | 896 (93.0) |
| Age ≥50 years at GLP-1RA initiation and clinical evidence of cardiovascular disease apart from inclusion criteria | 1136 (17.2) | 467 (16.0) | 669 (18.1) | 281 (14.8) | 487 (17.8) | 186 (18.3) | 182 (18.9) |
| Apart from medications stated in the inclusion criteria, no prescription for any DPP-4 inhibitor in the 30-days, pramlintide in the 90-days, or a diagnosis code for diabetic ketoacidosis in the 90-days before or at GLP-1RA initiation | 4481 (67.7) | 2040 (69.9) | 2441 (66.0) | 1291 (67.9) | 1757 (64.2) | 749 (73.6) | 684 (71.0) |
| No diagnosis code for solid organ transplant before or at GLP-1RA initiation | 6588 (99.6) | 2906 (99.6) | 3682 (99.6) | 1895 (99.7) | 2722 (99.5) | 1011 (99.4) | 960 (99.7) |
| No diagnosis code for end-stage renal disease before or at GLP-1RA initiation | 6595 (99.7) | 2907 (99.6) | 3688 (99.7) | 1896 (99.7) | 2729 (99.8) | 1011 (99.4) | 959 (99.6) |
| No diagnosis code for moderate to severe liver disease before or at GLP-1RA initiation | 6592 (99.6) | 2911 (99.8) | 3681 (99.5) | 1896 (99.7) | 2720 (99.5) | 1015 (99.8) | 961 (99.8) |
| **SUSTAIN 6 trial-eligible individuals** | **620 (9.4)** | **266 (9.1)** | **354 (9.6)** | **164 (8.6)** | **242 (8.8)** | **102 (10.0)** | **112 (11.6)** |
| Data are n (%). Abbreviations: SUSTAIN Semaglutide Unabated Sustainability in Treatment of Type 2 Diabetes, STEP Semaglutide Treatment Effect in People With Obesity, CVOT cardiovascular outcomes trial, GLP-1RA glucagon-like peptide-1 receptor agonist, HbA1c glycated hemoglobin, NYHA New York Heart Association, eGFR estimated glomerular filtration rate, BMI body-mass index, SGLT-2i sodium-glucose co-transporter-2 inhibitor. | | | | | | | |

| **Table S15: Subgroup analyses based on eligibility for the SUSTAIN 7,**^1^ **STEP 2,**^2^ **and SUSTAIN 6**^3^ **trials in the per-protocol analysis set for primary endpoints.** | | | | | |
| --- | --- | --- | --- | --- | --- |
| **Subgroup** | **Endpoint** | **Semaglutide** | **Dulaglutide** | **ETD**  **(95% CI)** | **p-value** |
| **SUSTAIN 7 trial-eligible**  Semaglutide, n = 256  Dulaglutide, n = 316 | Absolute change from baseline HbA1c, percentage points | -1.1 (0.13) | -1.02 (0.08) | -0.08 (-0.38, 0.22) | 0.596 |
|  | Relative change from baseline HbA1c, % | -13.38 (1.63) | -12.24 (1.02) | -1.14 (-4.91, 2.63) | 0.553 |
|  | Absolute change from baseline bodyweight, kg | -5.72 (1.89) | -4.18 (1.14) | -1.54 (-5.76, 2.68) | 0.474 |
|  | Relative change from baseline bodyweight, % | -4.68 (1.96) | -3.15 (1.1) | -1.52 (-5.79, 2.75) | 0.484 |
| **SUSTAIN 7 trial-ineligible**  Semaglutide, n = 1645  Dulaglutide, n = 2419 | Absolute change from baseline HbA1c, percentage points | -0.83 (0.03) | -0.6 (0.03) | -0.23 (-0.31, -0.15) | <0.0001 |
|  | Relative change from baseline HbA1c, % | -9.87 (0.4) | -6.92 (0.33) | -2.95 (-3.94, -1.95) | <0.0001 |
|  | Absolute change from baseline bodyweight, kg | -5.5 (0.45) | -3.49 (0.35) | -2.01 (-3.07, -0.95) | 0.0002 |
|  | Relative change from baseline bodyweight, % | -4.82 (0.48) | -2.88 (0.37) | -1.94 (-3.06, -0.82) | 0.0007 |
| **STEP 2 trial-eligible**  Semaglutide, n = 640  Dulaglutide, n = 927 | Absolute change from baseline HbA1c, percentage points | -1.03 (0.05) | -0.8 (0.04) | -0.23 (-0.36, -0.1) | 0.0007 |
|  | Relative change from baseline HbA1c, % | -12.33 (0.66) | -9.54 (0.53) | -2.78 (-4.39, -1.18) | 0.0007 |
|  | Absolute change from baseline bodyweight, kg | -5.52 (0.87) | -3.61 (0.56) | -1.91 (-3.91, 0.09) | 0.061 |
|  | Relative change from baseline bodyweight, % | -4.7 (0.91) | -2.84 (0.56) | -1.86 (-3.94, 0.22) | 0.0802 |
| **STEP 2 trial-ineligible**  Semaglutide, n = 1261  Dulaglutide, n = 1808 | Absolute change from baseline HbA1c, percentage points | -0.79 (0.04) | -0.58 (0.03) | -0.22 (-0.31, -0.12) | <0.0001 |
|  | Relative change from baseline HbA1c, % | -9.37 (0.47) | -6.62 (0.39) | -2.75 (-3.92, -1.57) | <0.0001 |
|  | Absolute change from baseline bodyweight, kg | -4.7 (0.62) | -2.97 (0.44) | -1.73 (-3.11, -0.35) | 0.0138 |
|  | Relative change from baseline bodyweight, % | -5.43 (0.52) | -3.58 (0.41) | -1.84 (-3.07, -0.62) | 0.0032 |
| **SUSTAIN 6 trial-eligible**  Semaglutide, n = 164  Dulaglutide, n = 242 | Absolute change from baseline HbA1c, percentage points | -0.83 (0.21) | -0.66 (0.12) | -0.17 (-0.63, 0.29) | 0.464 |
|  | Relative change from baseline HbA1c, % | -9.86 (2.89) | -7.73 (1.5) | -2.13 (-8.22, 3.96) | 0.492 |
|  | Absolute change from baseline bodyweight, kg | -5.32 (1.26) | -4.38 (1.23) | -0.93 (-4.37, 2.51) | 0.594 |
|  | Relative change from baseline bodyweight, % | -4.99 (1.35) | -3.95 (1.32) | -1.04 (-4.8, 2.72) | 0.587 |
| **SUSTAIN 6 trial-ineligible**  Semaglutide, n = 1737  Dulaglutide, n = 2493 | Absolute change from baseline HbA1c, percentage points | -0.86 (0.03) | -0.65 (0.03) | -0.21 (-0.29, -0.13) | <0.0001 |
|  | Relative change from baseline HbA1c, % | -10.29 (0.39) | -7.64 (0.33) | -2.64 (-3.61, -1.68) | <0.0001 |
|  | Absolute change from baseline bodyweight, kg | -5.54 (0.44) | -3.54 (0.37) | -2 (-3.05, -0.95) | 0.0002 |
|  | Relative change from baseline bodyweight, % | -4.77 (0.48) | -2.86 (0.38) | -1.91 (-3.03, -0.8) | 0.0008 |
| Data are presented as mean (SE) change from baseline, with mean (95% CI) estimated treatment difference (ETD); using data from individuals in the per-protocol analysis set (i.e., those who remained on their assigned treatment strategy for more than 210 days after cohort entry), while on-treatment. Abbreviations: SUSTAIN Semaglutide Unabated Sustainability in Treatment of Type 2 Diabetes, STEP Semaglutide Treatment Effect in People With Obesity, ETD estimated treatment difference, HbA1c glycated hemoglobin. | | | | | |

| **Table S16: Effectiveness outcomes measured over 6-months and 1-year in the full analysis set.** | | | | |
| --- | --- | --- | --- | --- |
| **Endpoints** | **Semaglutide**  **(n = 2918)** | **Dulaglutide**  **(n = 3698** | **Treatment comparison (95% CI)** | **p value** |
| **Primary endpoints measured as absolute and percentage changes from baseline over 1-year** | | | | |
| Absolute change from baseline HbA1c, percentage points | -0.83 (0.03) | -0.63 (0.02) | ETD -0.2 (-0.27, -0.13) | <0.0001 |
| Percentage change from baseline HbA1c, % | -10.38 (0.37) | -7.59 (0.32) | ETD -2.78  (-3.71, -1.85) | <0.0001 |
| Absolute change from baseline bodyweight, kg | -5.03 (0.35) | -3.51 (0.32) | ETD -1.52 (-2.41, -0.63) | 0.0009 |
| Percentage change from baseline bodyweight, % | -4.76 (0.45) | -2.92 (0.35) | ETD -1.85  (-2.9, -0.79) | 0.0006 |
| **Primary endpoints measured as absolute and percentage changes from baseline over 6-months** | | | | |
| Absolute change from baseline HbA1c, percentage points | -0.76 (0.03) | -0.65 (0.02) | ETD -0.12 (-0.18, -0.05) | 0.0003 |
| Percentage change from baseline HbA1c, % | -9.07 (0.32) | -7.55 (0.27) | ETD -1.52 (-2.32, -0.72) | 0.0002 |
| Absolute change from baseline bodyweight, kg | -3.73 (0.34) | -2.62 (0.32) | ETD -1.11 (-2.06, -0.16) | 0.0223 |
| Percentage change from baseline bodyweight, % | -3.03 (0.38) | -1.96 (0.33) | ETD -1.06 (-2.09, -0.04) | 0.0427 |
| **Secondary endpoints measured as absolute changes from baseline over 1-year** | | | | |
| BMI (kg/m^2^) | -1.82 (0.1) | -1.24 (0.08) | ETD -0.58 (-0.82, -0.33) | <0.0001 |
| Systolic blood pressure (mmHg) | -4.06 (0.41) | -2.29 (0.37) | ETD -1.77 (-2.84, -0.71) | 0.0011 |
| Diastolic blood pressure (mmHg) | -1.26 (0.26) | -0.58 (0.23) | ETD -0.68 (-1.37, 0) | 0.0516 |
| eGFR (ml/min/1.73m^2^) | -0.59 (0.24) | -0.61 (0.19) | ETD 0.02 (-0.57, 0.61) | 0.947 |
| **Secondary endpoints measured as absolute changes from baseline over 6-months** | | | | |
| BMI (kg/m^2^) | -1.38 (0.1) | -0.94 (0.09) | ETD -0.44 (-0.7, -0.18) | 0.0011 |
| Systolic blood pressure (mmHg) | -3.94 (0.41) | -2.39 (0.38) | ETD -1.56 (-2.63, -0.48) | 0.0045 |
| Diastolic blood pressure (mmHg) | -0.59 (0.26) | -0.6 (0.22) | ETD 0.01 (-0.66, 0.68) | 0.982 |
| eGFR (ml/min/1.73m^2^) | -0.23 (0.22) | -0.1 (0.18) | ETD -0.13 (-0.69, 0.43) | 0.656 |
| **Secondary endpoints measured as proportions of individuals achieving glycemic and weight management targets over 1-year** | | | | |
| Absolute HbA1c reduction ≥1% | 43.9% (1.3%) | 36.9% (1.1%) | OR 1.34 (1.17, 1.54) | <0.0001 |
| ≥3% percentage bodyweight reduction (% with SE) | 59.8% (1.4%) | 50.8% (1.2%) | OR 1.44 (1.25, 1.66) | <0.0001 |
| ≥5% percentage bodyweight reduction (% with SE) | 49.6% (1.4%) | 39.3% (1.1%) | OR 1.52 (1.32, 1.75) | <0.0001 |
| ≥10% percentage bodyweight reduction (% with SE) | 27.5% (1.2%) | 19.1% (1%) | OR 1.6 (1.35, 1.89) | <0.0001 |
| Composite of absolute HbA1c reduction ≥1% and percentage bodyweight reduction ≥3% | 29.3% (1.3%) | 21.1% (0.9%) | OR 1.55 (1.33, 1.82) | <0.0001 |
| **Secondary endpoints measured as proportions of individuals achieving glycemic and weight management targets over 6-months** | | | | |
| Absolute HbA1c reduction ≥1% | 41.7% (1.3%) | 36.9% (1%) | OR 1.22 (1.08, 1.39) | 0.0022 |
| ≥3% percentage bodyweight reduction (% with SE) | 53.3% (1.3%) | 45.9% (1.2%) | OR 1.35 (1.17, 1.55) | <0.0001 |
| ≥5% percentage bodyweight reduction (% with SE) | 42% (1.3%) | 33.7% (1.2%) | OR 1.42 (1.22, 1.66) | <0.0001 |
| ≥10% percentage bodyweight reduction (% with SE) | 21.1% (1.2%) | 16.1% (1%) | OR 1.39 (1.13, 1.71) | 0.0018 |
| Composite of absolute HbA1c reduction ≥1% and percentage bodyweight reduction ≥3% | 24.5% (1.1%) | 19.2% (0.8%) | OR 1.37 (1.16, 1.62) | 0.0002 |
| Data are presented as mean (SE) change from baseline, with mean (95% CI) estimated treatment difference (ETD), or as n (% and corresponding SE in %) with estimated odds ratio (OR) and corresponding 95% CI; using data from individuals in the full analysis set (i.e., eligible individuals who received at least one prescription for injectable semaglutide or dulaglutide), while on-treatment. Abbreviations: HbA1c glycated hemoglobin, BMI body-mass index, eGFR estimated glomerular filtration rate. | | | | |

| **Table S17: Effectiveness outcomes measured over 6-months in the per-protocol analysis set.** | | | | |
| --- | --- | --- | --- | --- |
| **Endpoints** | **Semaglutide**  **(n = 1901)** | **Dulaglutide**  **(n = 2735)** | **Treatment comparison (95% CI)** | **p value** |
| **Primary endpoints measured as absolute and percentage changes from baseline over 6-months** | | | | |
| Absolute change from baseline HbA1c, percentage points | -0.79 (0.03) | -0.66 (0.02) | ETD -0.13 (-0.2, -0.06) | 0.0004 |
| Percentage change from baseline HbA1c, % | -9.37 (0.35) | -7.74 (0.3) | ETD -1.63 (-2.51, -0.75) | 0.0003 |
| Absolute change from baseline bodyweight, kg | -3.98 (0.38) | -2.66 (0.34) | ETD -1.32 (-2.32, -0.32) | 0.0096 |
| Percentage change from baseline bodyweight, % | -3.28 (0.41) | -2.01 (0.35) | ETD -1.27 (-2.33, -0.2) | 0.0197 |
| **Secondary endpoints measured as absolute changes from baseline over 6-months** | | | | |
| BMI (kg/m^2^) | -1.46 (0.11) | -0.95 (0.09) | ETD -0.51 (-0.79, -0.23) | 0.0004 |
| Systolic blood pressure (mmHg) | -4.03 (0.47) | -2.36 (0.41) | ETD -1.68 (-2.87, -0.48) | 0.006 |
| Diastolic blood pressure (mmHg) | -0.6 (0.3) | -0.62 (0.25) | ETD 0.02 (-0.73, 0.77) | 0.965 |
| eGFR (ml/min/1.73m^2^) | -0.04 (0.25) | -0.08 (0.2) | ETD 0.04 (-0.58, 0.66) | 0.893 |
| **Secondary endpoints measured as proportions of individuals achieving glycemic and weight management targets over 6-months** | | | | |
| Absolute HbA1c reduction ≥1% | 43.6% (1.4%) | 37.3% (1.1%) | OR 1.3 (1.12, 1.5) | 0.0004 |
| ≥3% percentage bodyweight reduction (% with SE) | 54.7% (1.5%) | 46% (1.3%) | OR 1.42 (1.22, 1.66) | <0.0001 |
| ≥5% percentage bodyweight reduction (% with SE) | 42.4% (1.5%) | 33.1% (1.3%) | OR 1.49 (1.26, 1.76) | <0.0001 |
| ≥10% percentage bodyweight reduction (% with SE) | 20% (1.2%) | 15.4% (1.1%) | OR 1.38 (1.11, 1.73) | 0.0046 |
| Composite of absolute HbA1c reduction ≥1% and percentage bodyweight reduction ≥3% | 26.4% (1.3%) | 19.3% (1%) | OR 1.5 (1.25, 1.8) | <0.0001 |
| Data are presented as mean (SE) change from baseline, with mean (95% CI) estimated treatment difference (ETD), or as n (% and corresponding SE in %) with estimated odds ratio (OR) and corresponding 95% CI; using data from individuals in the per-protocol (i.e., primary) analysis set (i.e., individuals from the full analysis set who remained on their assigned treatment strategy for more than 210-days after cohort entry), while on-treatment. Abbreviations: HbA1c glycated hemoglobin, BMI body-mass index, eGFR estimated glomerular filtration rate. | | | | |

| **Table S18: Effectiveness outcomes measured over 6-months in the early-attrition analysis set.** | | | | |
| --- | --- | --- | --- | --- |
| **Endpoints** | **Semaglutide**  **(n = 1017)** | **Dulaglutide**  **(n = 963)** | **Treatment comparison (95% CI)** | **p value** |
| **Primary endpoints measured as absolute and percentage changes from baseline over 6-months** | | | | |
| Absolute change from baseline HbA1c, percentage points | -0.71 (0.05) | -0.61 (0.04) | -0.11 (-0.23, 0.02) | 0.107 |
| Percentage change from baseline HbA1c, % | -8.41 (0.6) | -7.07 (0.57) | -1.34 (-2.95, 0.27) | 0.103 |
| Absolute change from baseline bodyweight, kg | -3.26 (0.6) | -2.62 (0.63) | -0.64 (-2.39, 1.11) | 0.47 |
| Percentage change from baseline bodyweight, % | -2.54 (0.67) | -1.93 (0.65) | -0.61 (-2.48, 1.27) | 0.523 |
| **Secondary endpoints measured as absolute changes from baseline over 6-months** | | | | |
| BMI (kg/m^2^) | -1.22 (0.17) | -0.94 (0.16) | -0.28 (-0.75, 0.2) | 0.253 |
| Systolic blood pressure (mmHg) | -4.01 (0.72) | -2.47 (0.76) | -1.54 (-3.62, 0.54) | 0.147 |
| Diastolic blood pressure (mmHg) | -0.64 (0.49) | -0.5 (0.47) | -0.13 (-1.47, 1.21) | 0.847 |
| eGFR (ml/min/1.73m^2^) | -0.51 (0.4) | -0.21 (0.38) | -0.3 (-1.39, 0.8) | 0.595 |
| **Secondary endpoints measured as proportions of individuals achieving glycemic and weight management targets over 6-months** | | | | |
| Absolute HbA1c reduction ≥1% | 38.2% (2.3%) | 35.8% (2.1%) | 1.11 (0.85, 1.44) | 0.435 |
| ≥3% percentage bodyweight reduction (% with SE) | 50.6% (2.3%) | 45.8% (2.4%) | 1.21 (0.94, 1.57) | 0.146 |
| ≥5% percentage bodyweight reduction (% with SE) | 41.1% (2.3%) | 35.6% (2.3%) | 1.27 (0.96, 1.67) | 0.0994 |
| ≥10% percentage bodyweight reduction (% with SE) | 22.6% (2%) | 18.1% (2%) | 1.32 (0.92, 1.9) | 0.128 |
| Composite of absolute HbA1c reduction ≥1% and percentage bodyweight reduction ≥3% | 21% (1.9%) | 18.7% (1.9%) | 1.16 (0.83, 1.6) | 0.386 |
| Data are presented as mean (SE) change from baseline, with mean (95% CI) estimated treatment difference (ETD), or as n (% and corresponding SE in %) with estimated odds ratio (OR) and corresponding 95% CI; using data from individuals in the early-attrition analysis set (i.e., individuals in the full analysis set who remained on their assigned treatment strategy for 210-days or less), while on-treatment. Abbreviations: HbA1c glycated hemoglobin, BMI body-mass index, eGFR estimated glomerular filtration rate. | | | | |

| **Table S19: Summary of endpoint estimates from the SUSTAIN 7,**^1^ **STEP 2,**^2^ **and SUSTAIN 6**^3^ **trials.** | | | | | |
| --- | --- | --- | --- | --- | --- |
| **Trial:**  **Treatment strategy 1,**  **treatment strategy 2** | **Endpoint** | **Treatment strategy 1** | **Treatment strategy 2** | **Treatment comparison**  **(95% CI)** | **p-value** |
| **SUSTAIN 7 trial^a^**  **Low dose comparison**  Semaglutide 0.5 mg, n = 301  Dulaglutide 0.75 mg, n = 299 | Absolute change from baseline HbA1c, percentage points | -1.5 (0.06) | -1.1 (0.05) | ETD -0.40 (-0.55 to -0.25) | <0.0001 |
|  | Absolute change from baseline bodyweight, kg | -4.6 (0.28) | -2.3 (0.27) | ETD -2.26 (-3.02 to -1.51) | <0.0001 |
|  | Absolute change from baseline BMI, kg/m^2^ | -1.6 (0.10) | -0.8 (0.10) | ETD -0.81 (-1.08 to -0.54) | <0.0001 |
|  | Absolute change from baseline systolic blood pressure, mmHg | -2.4 (0.76) | -2.2 (0.75) | ETD -0.28 (-2.37 to 1.81) | 0.79 |
|  | Absolute change from baseline diastolic blood pressure, mmHg | -0.6 (0.48) | -0.3 (0.47) | ETD -0.22 (-1.54 to 1.10) | 0.74 |
|  | ≥5% percentage bodyweight reduction | 132 (44%) | 68 (23%) | OR 2.40 (1.65-3.47) | <0.0001 |
|  | ≥10% percentage bodyweight reduction | 43 (14%) | 10 (3%) | OR 4.79 (2.38-9.65) | <0.0001 |
|  | Composite of absolute HbA1c reduction ≥1% and percentage bodyweight reduction ≥3% | 160 (53%) | 75 (25%) | OR 2.82 (1.95-4.08) | <0.0001 |
| **SUSTAIN 7 trial^a^**  **High dose comparison**  Semaglutide 1.0 mg, n = 300  Dulaglutide 1.5 mg, n = 299 | Absolute change from baseline HbA1c, percentage points | -1.8 (0.06) | -1.4 (0.06) | ETD -0.41 (-0.57 to -0.25) | <0.0001 |
|  | Absolute change from baseline bodyweight, kg | -6.5 (0.28) | -3.0 (0.27) | ETD -3.55 (-4.32 to -2.78) | <0.0001 |
|  | Absolute change from baseline BMI, kg/m^2^ | -2.3 (0.10) | -1.1 (0.10) | ETD -1.25 (-1.52 to -0.98) | <0.0001 |
|  | Absolute change from baseline systolic blood pressure, mmHg | -4.9 (0.77) | -2.9 (0.75) | ETD -2.02 (-4.14 to 0.09) | 0.0607 |
|  | Absolute change from baseline diastolic blood pressure, mmHg | -2.0 (0.49) | <-0.1 (0.47) | ETD -2.02 (-3.35 to -0.68) | 0.0031 |
|  | ≥5% percentage bodyweight reduction | 189 (63%) | 90 (30%) | OR 3.03 (2.11-4.34) | <0.0001 |
|  | ≥10% percentage bodyweight reduction | 80 (27%) | 23 (8%) | OR 4.55 (2.73-7.59) | <0.0001 |
|  | Composite of absolute HbA1c reduction ≥1% and percentage bodyweight reduction ≥3% | 205 (68%) | 104 (35%) | OR 3.11 (2.17-4.46) | <0.0001 |
| **STEP 2 trial^b^**  Semaglutide 2.4 mg, n = 404  Semaglutide 1.0 mg, n = 403 | Absolute change from baseline HbA1c, percentage points | -1.6 (0.1) | -1.5 (0.1) | ETD -0.2  (-0.3 to 0.0) | NA |
|  | Absolute change from baseline bodyweight, kg | -9.7 (0.4) | -6.9 (0.4) | ETD -2.7  (-3.8 to -1.7) | NA |
|  | Relative change from baseline bodyweight, % | -9.64 (0.4) | -6.99 (0.4) | ETD -2.65  (-3.66 to -1.64) | <0.0001 |
|  | ≥5% percentage bodyweight reduction | 267/388 (68.8%) | 217/380 (57.1%) | OR 1.62  (1.21 to 2.18) | NA |
| **SUSTAIN 6 trial^c^**  Semaglutide 0.5 mg, n = 826  Semaglutide 1.0 mg, n = 822 | Absolute change from baseline HbA1c, percentage points | -1.1 | -1.4 | NA (was compared to placebo) | NA |
|  | Absolute change from baseline bodyweight, kg | -3.6 | -4.9 | NA (was compared to placebo) | NA |
|  | Absolute change from baseline systolic blood pressure, mmHg | -3.44 | -5.37 | NA (was compared to placebo) | NA |
|  | Absolute change from baseline diastolic blood pressure, mmHg | -1.37 | -1.57 | NA (was compared to placebo) | NA |
| Data are presented as mean (SE) change from baseline, with mean (95% CI) estimated treatment difference (ETD), or as n (%) with estimated odds ratio (OR) and corresponding 95% CI; where available. ^a^Data for all randomized patients exposed to at least one dose of trial product, while on-treatment (based on change from baseline to week 40). ^b^Data for all patients in the full analysis set (i.e., intention-to-treat analysis; based on change from baseline to week 68). ^c^In-trial data for scheduled visits for the full analysis set (i.e., intention-to-treat analysis; based on change from baseline to week 104). Abbreviations: SUSTAIN Semaglutide Unabated Sustainability in Treatment of Type 2 Diabetes, STEP Semaglutide Treatment Effect in People With Obesity, HbA1c glycated hemoglobin, ETD estimated treatment difference, body-mass index, NA not available. | | | | | |

| **Table S20: Selected baseline characteristics of participants (randomized to once-weekly subcutaneous semaglutide 1.0 mg) in the SUSTAIN 7,**^1^ **STEP 2,**^2^ **and SUSTAIN 6**^3^ **trials, along with those of individuals (assigned to once-weekly subcutaneous semaglutide at cohort entry) in the per-protocol analysis set who were considered eligible for the corresponding trials (Supplementary Table S14).** | | | | | | |
| --- | --- | --- | --- | --- | --- | --- |
| **Characteristics** | **Clinical trial participants** | | | **Individuals considered eligible for corresponding clinical trials**  **(based on emulation of corresponding eligibility criteria)** | | |
| **Treatment strategy** | **Once-weekly subcutaneous semaglutide 1.0 mg** | | | **Once-weekly subcutaneous semaglutide** | | |
| **Trial** | **SUSTAIN 7 trial** | **STEP 2 trial** | **SUSTAIN 6 trial** | **SUSTAIN 7 trial** | **STEP 2 trial** | **SUSTAIN 6 trial** |
| n | 300 | 403 | 822 | 254 | 237 | 163 |
| **Demographic characteristics** | | | | | | |
| Age, years | 55 (10.6) | 56 (10) | 64.7 (7.1) | 54.4 (12.7) | 56.2 (11.4) | 65.4 (7.7) |
| Sex assigned at birth: Female | 8.2 (0.9) | 203 (50.4%) | 304 (27%) | 140 (55.1%) | 319 (50.1%) | 52 (31.9%) |
| Type 2 diabetes duration, years | 7.3 (5.7) | 7.7 (5.9) | 14.1 (8.17) | 5.8 (6.8) | 5.5 (5.5) | 7.7 (6.6) |
| **Glucose-lowering therapies** | | | | | | |
| Metformin | On-metformin only | 379 (94.0%) | 594 (72.3%) | 254 (100%) | 569 (89.3%) | 138 (84.7%) |
| Sulfonylureas | NA | 99 (24.6%) | 349 (42.5) | <7 | 243 (38.1%) | 63 (38.7%) |
| Sodium-glucose co-transporter-2 inhibitors | NA | 96 (23.8%) | 23 (2.8) 1 (0.1) | 19 (7.5%) | 359 (56.4%) | 82 (50.3%) |
| Dipeptidyl peptidase-4 inhibitors | NA | 3 (0.7%) | 2 (0.2) | 9 (3.5%) | 33 (5.2%) | 42 (25.8%) |
| Thiazolidinediones | NA | 16 (4.0%) | 21 (2.6) | <7 | 23 (3.6%) | <7 |
| Insulin | NA | 0 | 259 (31.5) | 50 (19.7%) | <7 | 46 (28.2%) |
| Number of glucose-lowering therapies ≥3 | NA | 3: 29 (7.2%)  4: 1 (0.2%) | NA | 7 (2.8%) | 176 (27.6%) | 72 (44.2%) |
| **Clinical characteristics** | | | | | | |
| Bodyweight, kg | 95.5 (20.9) | 99.0 (21.1) | 92.9 (21.05) | 108.1 (19.5) | 105.2 (18.9) | 100.9 (19) |
| BMI, kg/m^2^ | 33.6 (6.5) | 35.3 (5.9) | 32.9 (6.18) | 37.5 (5.6) | 36.4 (5.2) | 34.8 (5.2) |
| HbA1c, % | 8.2 (0.9) | 8.1% (0.8) | 8.7 (1.51) | 7.9 (0.6) | 8 (0.6) | 8.1 (0.6) |
| Systolic blood pressure, mmHg | 133 (14.5) | 130 (14) | 135.8 (16.96) | 134 (13.3) | 133.3 (14.2) | 132.1 (15.7) |
| Diastolic blood pressure, mmHg | 82 (9.1) | 80 (9) | 76.9 (10.21) | 80 (10.3) | 79.2 (9.3) | 74.7 (10.5) |
| eGFR, ml/min/1.73m^2^ | 97 (17.2) | 93.43 (21.43) | eGFR ≥90: 246 (29.9%) | 102.3 (17.9) | 100.7 (18.6) | 83.8 (21.3) |
| Cardiovascular disease | NA | Coronary artery disease: 26 (6.4%) | Ischemic heart disease: 495 (60.2%) | 34 (13.4%) | 83 (13%) | 163 (100%) |
| Myocardial infarction | NA | NA | 64 (32.1%) | 13 (5.1%) | 32 (5%) | 64 (39.3%) |
| Stroke | NA | NA | Ischemic: 89 (10.8%)  Hemorrhagic: 24 (2.9) | 9 (3.5%) | 23 (3.6%) | 49 (30.1%) |
| Heart failure | NA | NA | 180 (21.9%) | 10 (3.9%) | 21 (3.3%) | 37 (22.7%) |
| Data presented in n (%) or mean (SD). Counts <7 are suppressed to prevent person identification. Abbreviations: Abbreviations: SUSTAIN Semaglutide Unabated Sustainability in Treatment of Type 2 Diabetes, STEP Semaglutide Treatment Effect in People With Obesity, NA not available, BMI body-mass index, HbA1c glycated hemoglobin, eGFR estimated glomerular filtration rate. | | | | | | |

SUPPLEMENTARY FIGURES

| **** |
| --- |
| **Figure S1: Propensity score distributions of semaglutide versus dulaglutide initiators in the full analysis set, before and after weighting.** |

| **** |
| --- |
| **Figure S2: Propensity score distributions of semaglutide versus dulaglutide initiators in the per-protocol analysis set, before and after weighting.** |

| **** |
| --- |
| **Figure S3: Propensity score distributions of semaglutide versus dulaglutide initiators in the early-attrition analysis set, before and after weighting.** |

| **** |
| --- |
| **Figure S4: Convergence diagnostics for multiple imputation by chained equations among once-weekly injectable semaglutide initiators.** |
| Trace plots showing the mean (left column) and standard deviation (right column) of imputed values across 50 iterations for each outcome variable. Each colored line represents one of 100 imputed datasets. Stable, overlapping trajectories indicate successful convergence of the imputation algorithm. |

| **** |
| --- |
| **Figure S5: Convergence diagnostics for multiple imputation by chained equations among once-weekly injectable dulaglutide initiators.** |
| Trace plots showing the mean (left column) and standard deviation (right column) of imputed values across 50 iterations for each outcome variable. Each colored line represents one of 100 imputed datasets. Stable, overlapping trajectories indicate successful convergence of the imputation algorithm. Abbreviations: HbA1c glycated hemoglobin, BP blood pressure, eGFR estimated glomerular filtration rate. |

| **** |
| --- |
| **Figure S6: Distribution comparison of observed versus imputed outcome values among once-weekly injectable semaglutide initiators.** |
| Density plots comparing the distributions of observed (grey) and imputed (red) values for each outcome variable at follow-up. Close alignment between observed and imputed distributions indicates that imputed values are consistent with the observed data and remain within biologically plausible ranges. Abbreviations: HbA1c glycated hemoglobin, BP blood pressure, eGFR estimated glomerular filtration rate. |

| **** |
| --- |
| **Figure S7: Distribution comparison of observed versus imputed outcome values among once-weekly injectable dulaglutide initiators.** |
| Density plots comparing the distributions of observed (grey) and imputed (red) values for each outcome variable at follow-up. Close alignment between observed and imputed distributions indicates that imputed values are consistent with the observed data and remain within biologically plausible ranges. Abbreviations: HbA1c glycated hemoglobin, BP blood pressure, eGFR estimated glomerular filtration rate. |

# SUPPLEMENTARY REFERENCES

1. Pratley, R. E. *et al.* Semaglutide versus dulaglutide once weekly in patients with type 2 diabetes (SUSTAIN 7): a randomised, open-label, phase 3b trial. *Lancet Diabetes Endocrinol* **6**, 275–286 (2018).

2. Davies, M. *et al.* Semaglutide 2·4 mg once a week in adults with overweight or obesity, and type 2 diabetes (STEP 2): a randomised, double-blind, double-dummy, placebo-controlled, phase 3 trial. *The Lancet* **397**, 971–984 (2021).

3. Marso, S. P. *et al.* Semaglutide and Cardiovascular Outcomes in Patients with Type 2 Diabetes. *N Engl J Med* **375**, 1834–1844 (2016).

4. Gerstein, H. C. *et al.* Dulaglutide and cardiovascular outcomes in type 2 diabetes (REWIND): a double-blind, randomised placebo-controlled trial. *The Lancet* **394**, 121–130 (2019).

5. National Institute for Health and Care Excellence. Type 2 diabetes in adults: management | Guidance | NICE. https://www.nice.org.uk/guidance/ng28 (2022). (accessed 07-Sept-2025).

6. Lund, J. L., Richardson, D. B. & Stürmer, T. The Active Comparator, New User Study Design in Pharmacoepidemiology: Historical Foundations and Contemporary Application. *Current Epidemiology Reports 2015 2:4* **2**, 221–228 (2015).

7. Rubin, D. B. *Multiple Imputation for Nonresponse in Surveys*. (John Wiley & Sons, Inc., 1987).

8. Van Buuren, S. Multiple imputation of discrete and continuous data by fully conditional specification. *Stat Methods Med Res* **16**, 219–242 (2007).

9. van Buuren, S. & Groothuis-Oudshoorn, K. mice: Multivariate Imputation by Chained Equations in R. *J Stat Softw* **45**, 1–67 (2011).

10. Bidulka, P. *et al.* Comparative effectiveness of second line oral antidiabetic treatments among people with type 2 diabetes mellitus: emulation of a target trial using routinely collected health data.

11. CKD-EPI Equations for Glomerular Filtration Rate (GFR). https://www.mdcalc.com/calc/3939/ckd-epi-equations-glomerular-filtration-rate-gfr. (accessed 07-Sept-2025).

12. QOF 2023-24 | NHS Digital. https://qof.digital.nhs.uk/. (accessed 07-Sept-2025).

13. Wilkinson, S. *et al.* Comparative effects of sulphonylureas, dipeptidyl peptidase-4 inhibitors and sodium-glucose co-transporter-2 inhibitors added to metformin monotherapy: a propensity-score matched cohort study in UK primary care. *Diabetes Obes Metab* **22**, 847–856 (2020).

14. Bidulka, P. *et al.* Comparative effectiveness of second line oral antidiabetic treatments among people with type 2 diabetes mellitus: emulation of a target trial using routinely collected health data. *BMJ* **385**, (2024).

15. NHS Health Research Authority - Derby Research Ethics Committee. IQVIA Medical Research Data - NHS Health Research Authority. https://www.hra.nhs.uk/planning-and-improving-research/application-summaries/research-summaries/iqvia-medical-research-data/ (2023). (accessed 07-Sept-2025).

16. Yucel Karakaya, S. P. & Unal, I. Balance diagnostics in propensity score analysis following multiple imputation: A new method. *Pharm Stat* **23**, 763–777 (2024).
